# Progressive muscle relaxation in pandemic times: bolstering medical student resilience through IPRMP and Gagne's model

**DOI:** 10.3389/fpsyg.2024.1240791

**Published:** 2024-03-13

**Authors:** Bhavana Nair, Sara Khan, Nerissa Naidoo, Shirin Jannati, Balamohan Shivani, Yajnavalka Banerjee

**Affiliations:** ^1^College of Medicine and Health Sciences, Mohammed Bin Rashid University of Medicine and Health Sciences (MBRU, Dubai Health), Dubai, United Arab Emirates; ^2^Centre for Medical Education, University of Dundee, Dundee, United Kingdom

**Keywords:** medical education, COVID-19 pandemic, distance learning, Progressive Muscle Relaxation (PMR), competency-based medical curricula (CBMC), Gagne's Nine Events of Instruction, thematic analysis, Bourdieu's Theory of Practice

## Abstract

**Background:**

Medical education, already demanding, has been further strained by the COVID-19 pandemic's challenges and the shift to distance learning. This context underscores the need for effective stress reduction techniques in competency-based medical curricula (CBMC).

**Objective:**

We assessed the feasibility and benefits of integrating a Progressive Muscle Relaxation (PMR) module—a known effective stress-reducing technique—into a time-restricted CBMC, particularly given such modules often find placement as elective rather than mandatory.

**Methods:**

Adapting Gagne's nine events of instruction, a 2-h PMR program was designed and implemented during the pandemic. Twenty participants were engaged on a first-come, first-served basis, ensuring adherence to social distancing measures. Feedback was continuously gathered, leading to two post-program focus group sessions. Qualitative data underwent thematic analysis following Braun and Clarke's approach, with study quality maintained by the Standards for Reporting Qualitative Research (SRQR). To gauge adaptability, we aligned the program with various learning outcomes frameworks and explored its fit within CBMC using Bourdieu's Theory of Practice.

**Results:**

The pilot PMR program was well-received and effectively incorporated into our CBMC. Our analysis revealed five central themes tied to PMR's impact: Self-control, Self-realization, Liberation, Awareness, and Interpersonal relationships. Feedback indicated the program's capacity to mitigate stress during the pandemic. The SRQR confirmed the study's alignment with qualitative research standards. Further, the PMR program's contents resonated with principal domains of learning outcomes, and its integration into CBMC was supported by Bourdieu's Theory. These observations led us to propose the Integrative Psychological Resilience Model in Medical Practice (IPRMP), a model that captures the intricate interplay between the identified psychological constructs.

**Conclusion:**

This research showcases an innovative, theory-guided approach to embed a wellbeing program within CBMC, accentuating PMR's role in fostering resilience among medical students. Our PMR model offers a feasible, cost-effective strategy suitable for global adoption in medical institutions. By instilling resilience and advanced stress-management techniques, PMR ensures that upcoming healthcare professionals are better equipped to manage crises like pandemics efficiently.

## 1 Introduction

Medical education, demanding both in its intensity and scope, requires students to display not only intellectual prowess but also resilience to meet the diverse healthcare needs of populations (Rees and Wass, 1993). Furthermore, the inherent rigor of the curriculum and extended clinical practices cause medical students to grapple with heightened levels of mental and physical stress, often more than peers in other academic fields (Tennant et al., 2007; Rotenstein et al., 2018; Afshar et al., 2022; Slimmen et al., 2022). The advent of the COVID-19 pandemic only added to this stress, triggering a widespread shift to distance learning, and prompting medical institutions to re-evaluate traditional teaching methods (Khalil et al., 2020; Afshan et al., 2022; Altaf et al., 2022).

In light of this, the post-pandemic era emphasizes the need for resilience and emotional intelligence within the healthcare profession (Chen et al., 2021; Kwon, 2023). Medical professionals are now tasked with adapting to the evolving demands of this time (Pollock et al., 2020; Di Giuseppe et al., 2021; Jia et al., 2022; Blake et al., 2023). Thus, it becomes imperative for medical educators to weave stress-reduction techniques and resilience-building strategies into the fabric of competency-based medical curricula (CBMC), aiming to safeguard the mental wellbeing of future doctors (Slavin et al., 2014; Edmonds et al., 2023; Lister et al., 2023).

Prioritizing these strategies within core curricula—instead of consigning them to elective courses or infrequent workshops—takes a holistic stance on medical students' mental health needs. This all-encompassing approach recognizes resilience and emotional intelligence as crucial components of comprehensive medical training, preparing students to effectively navigate challenges, be it future pandemics or other crises (Banerjee et al., 2019a; Shuo et al., 2022; Bursky et al., 2023; Brandao et al., 2024).

Proven stress-alleviating methods, ranging from mindfulness practices (Chmielewski et al., 2021; Harrison et al., 2024; Yosep et al., 2024) and physical activity (Schultchen et al., 2019; Hachenberger et al., 2023) to art therapy (Shukla et al., 2022) and counseling (Callus et al., 2020), aid in cultivating coping skills and maintaining students' mental equilibrium. Rooted in psychology, these interventions promote self-awareness, self-regulation, and effective interpersonal interactions, paving the way for a mentally resilient healthcare workforce (Keng et al., 2011; Heinrich and O'Connell, 2024; Kajee et al., 2024).

Incorporating such programs directly into CBMC is critical. Elective stress-relief courses often report lower participation, attracting only a subset of students (Brami et al., 2023). A more encompassing approach, as argued by Warnecke et al. (2011), is to arm medical students, the future healthcare providers, with proven strategies to manage their stress. The goal of this study is to pinpoint a method that effectively addresses stress and boosts resilience and emotional intelligence in undergraduate medical education, ensuring its seamless and cost-effective inclusion into standard CBMC.

### 1.1 Needs assessment and identification of the stress reduction technique to integrate

Following a needs-assessment that employed the nominal group technique—a structured method for group brainstorming that prioritizes ideas (Naidoo et al., 2021)—we discerned that mindfulness-based stress reduction (MBSR) stands out as the foremost stress mitigation approach in medical education. MBSR, a structured program that combines mindfulness meditation and yoga, has been effectively used to reduce distress and bolster psychological resilience among undergraduate medical students. While an exhaustive review of MBSR's role in enhancing resilience in medical education is presented below, it's worth noting that its application has predominantly been through supplementary workshops and programs rather than being seamlessly integrated into the main medical curriculum.

MBSR, a psychotherapeutic intervention, has demonstrated efficacy in mitigating anxiety, depression, and chronic pain (Valluri et al., 2024). Subsequently, MBSR has been extensively incorporated into medical education, offering both students and healthcare professionals a proven strategy for stress reduction and promoting mental health resilience (Selic-Zupancic et al., 2023). Building upon this, Supplementary Table 1 presents a comprehensive review of the empirical studies that delineate the utilization and effectiveness of MBSR within medical education for students and practitioners alike. In fact, MBSR is increasingly recognized as a salient adjunct in medical education, given its robust potential to attenuate stress and augment learning outcomes. Theoretical frameworks, such as the *Cognitive Load Theory (CLT)*, suggest that stress can impede cognitive functions critical for learning by adding extraneous load (Heer et al., 2021); thus, reducing stress through MBSR can optimize intrinsic and germane cognitive load, enhancing learning efficiency. This theory posits that learners have a limited capacity in their working memory, and stress, acting as an extraneous load, can overwhelm this capacity, inhibiting the processing and retention of new information. By mitigating stress, MBSR may thus facilitate a more efficient cognitive processing, allowing for improved comprehension and consolidation of complex medical curricula. Moreover, the integration of MBSR in medical training aligns with the principles of the *Affective Filter Hypothesis* (Ma, 2022), which underscores the influence of emotional states on learning. High levels of stress are posited to raise the affective filter, creating a psychological barrier to language acquisition and learning. MBSR, by promoting relaxation and emotional regulation, can lower this filter, thereby enhancing learners' receptivity to new information and fostering a more conducive environment for learning. Furthermore, the *Self-Determination Theory (SDT)* (Manzano-Sanchez, 2023), which emphasizes the role of autonomy, competence, and relatedness in motivation, suggests that the stress reduction from MBSR may enhance these psychological needs, thus fostering greater intrinsic motivation among medical students. This intrinsic motivation is crucial for deep learning and mastery of the complex skills required in the medical field. Additionally, the principles of *Experiential Learning Theory (ELT)* align with MBSR's emphasis on awareness and reflective practice (Kong, 2021). ELT posits that learning is a process whereby knowledge is created through the transformation of experience. MBSR facilitates this process by enabling students to approach clinical experiences with greater mindfulness, potentially leading to deeper reflection, critical analysis, and assimilation of learning experiences. Therefore, by diminishing stress, MBSR may enhance medical education through the lens of multiple learning theories. It alleviates cognitive overload (CLT), lowers affective filters (Affective Filter Hypothesis), supports intrinsic motivation (SDT), and fosters experiential learning (ELT). These theoretical implications are further substantiated by empirical evidence as delineated in Supplementary Table 1, underscoring MBSR's role in not only fostering psychological wellbeing but also in potentiating the learning process for medical students and professionals.

However, we acknowledged that the implementation and integration of MBSR within a Competency-Based Medical Education (CBME) system confronts numerous barriers. These include the limited availability of skilled instructors, significant financial and resource needs, and time constraints such as the fixed duration of courses, the variable pace of student learning, and the substantial time needed for competency assessment and administration. Additionally, there are challenges related to the complexities of integrating a new method into an established curriculum, resistance to change from those accustomed to traditional methods, and considerable dropout rates. These obstacles highlight the need for a well-managed implementation strategy and adaptive time management skills among learners.

Also, the multifaceted influence of religion on medical education in the Middle East encompasses a confluence of cultural, social, and epistemological factors, engendering unique pedagogical paradigms (Elbarazi et al., 2017; Tayeb et al., 2023). The prevailing Islamic ethos in the region imbues medical curricula with nuanced ethical considerations and axiological precepts, fostering an integrative approach to biopsychosocial models of health and disease (Fekih-Romdhane et al., 2023). The intricate interplay between religiosity and pedagogical methodologies necessitates the examination of contextual factors, such as prevailing sociocultural ethos and the assimilation of traditional and religious values into academic frameworks. Considering these complexities, the implementation of MBSR as a technique to augment resilience and reduce stress in undergraduate medical education may be deemed unsuitable due to its historical and epistemological roots in Buddhist philosophy, specifically the Vipassana tradition (Sharf, 2015; Sanivarapu, 2016). Although MBSR, a secularized intervention paradigm developed by Dr. Jon Kabat-Zinn (Noonan, 2014), has been meticulously extricated from its religious origins to ensure broader applicability in contemporary psychological and clinical domains, its intrinsic linkage to Buddhist underpinnings might engender discordance within the context of the region's predominant Islamic ethos. Hence, the operationalization of MBSR as an efficacious therapeutic modality within the scientific milieu warrants cautious deliberation, considering the potential incongruity with the Middle Eastern medical education landscape, to preserve its secular orientation and universal accessibility, while concurrently respecting and accommodating the region's religious and cultural sensitivities.

Furthermore, the implementation of MBSR as a therapeutic modality in the context of the Middle East may be met with trepidation, given the prevailing sociocultural landscape wherein mental health issues are frequently stigmatized and underreported (see Appendix 1). MBSR, an intervention paradigm predicated on cultivating non-judgmental awareness, present-moment attentiveness, and metacognitive aptitude, has demonstrated considerable efficacy in ameliorating psychological distress and bolstering resilience across diverse populations. However, the potential correlation between MBSR practice and mental health challenges, particularly in a region characterized by religious and cultural sensitivities, necessitates circumspect deliberation when contemplating its integration into medical curricula.

To circumvent these barriers and to ensure student participation and compliance, it is imperative to identify alternative therapeutic techniques that coalesce with the region's unique milieu, while maintaining a robust evidence base of efficacy in enhancing psychological wellbeing and resilience. Such an approach would enable the development of a culturally congruent pedagogical paradigm, fostering greater receptivity and engagement among medical students.

### 1.2 Progressive muscle relaxation (PMR): a culturally congruent and efficacious alternative for enhancing resilience and reducing stress

Upon careful review and examination of the literature, PMR emerges as a superior technique for stress reduction and resilience enhancement within the context of undergraduate medical education (Burleson et al., 2023; Tating et al., 2023). The technique, developed by Edmund Jacobson in the 1920s, operates on the premise that psychological stress is intrinsically linked to muscular tension; hence, by systematically tensing and relaxing distinct muscle groups, PMR fosters a state of profound physical and mental relaxation (Toussaint et al., 2021).

Both PMR and MBSR exert salutary effects by mediating the relaxation response, which serves as a counter-regulatory process to stress-induced responses governed by the hypothalamic-pituitary-adrenal (HPA) axis (Thakur et al., 2023). The HPA axis, an integral component of the neuroendocrine system, plays a pivotal role in coordinating the body's response to stress (Leistner and Menke, 2020). Initiated by the hypothalamus's secretion of corticotropin-releasing hormone (CRH), the axis stimulates the pituitary gland to release adrenocorticotropic hormone (ACTH), culminating in the adrenal glands' production of cortisol, a potent stress hormone. Under chronic stress conditions, persistent activation of the HPA axis and subsequent hypercortisolemia can lead to deleterious effects, including immunosuppression, metabolic dysregulation, and neuropsychiatric disorders (James et al., 2023; Rusch et al., 2023).

**Figure 1:**
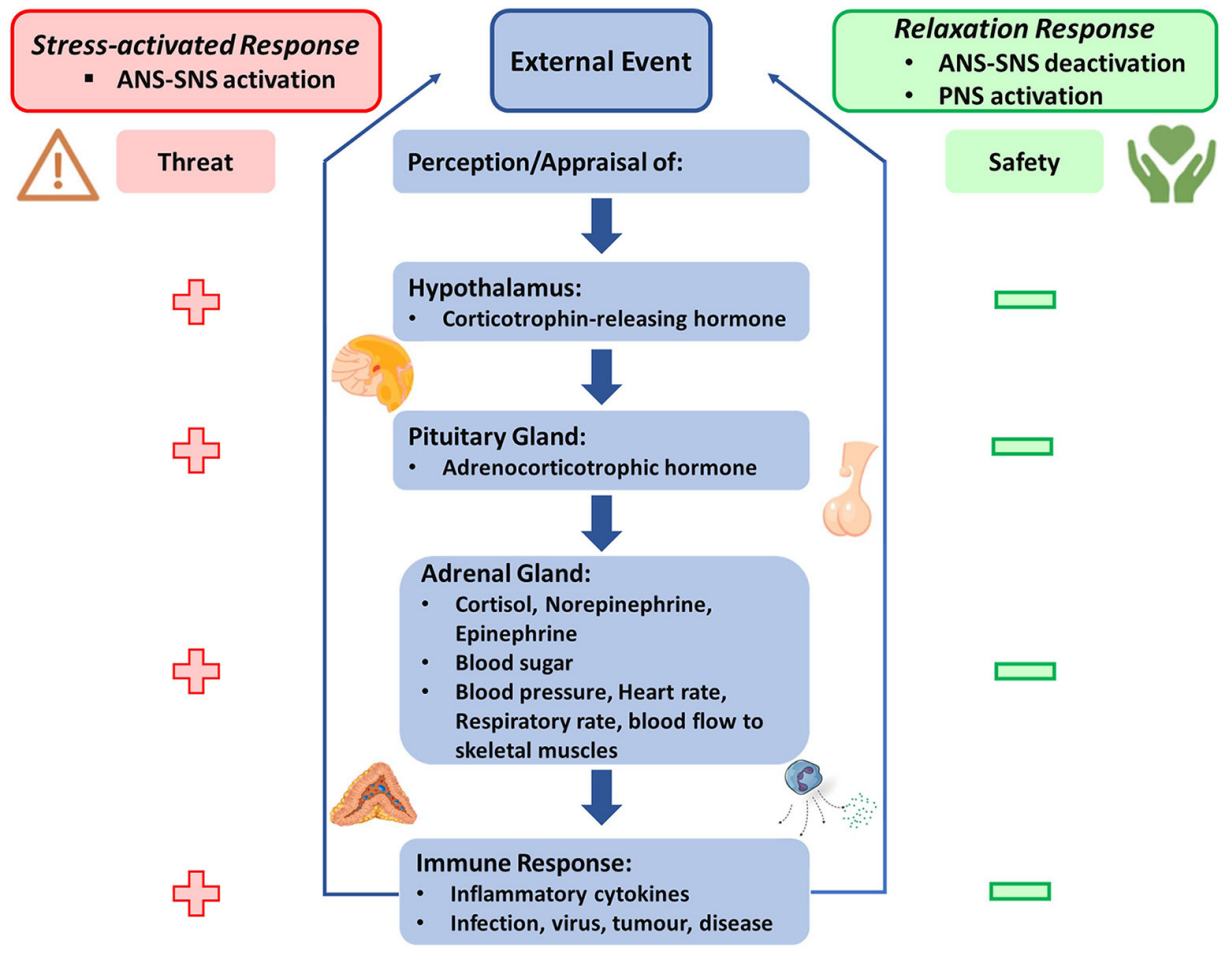
Schematic representation of the hypothalamic-pituitary-adrenal (HPA) axis modulation by the relaxation response elicited through techniques such as PMR and MBSR [note: under stress, the HPA axis activates, leading to the secretion of corticotropin-releasing hormone (CRH) from the hypothalamus, which stimulates the pituitary gland to release adrenocorticotropic hormone (ACTH). ACTH triggers the adrenal glands to release cortisol, a stress hormone. Chronic stress can result in continuous HPA axis activation and elevated cortisol levels, leading to negative health effects. PMR and MBSR stimulate the relaxation response, potentially enhancing inhibitory feedback mechanisms and fostering resilience to stressors. This response attenuates HPA axis hyperactivity and excessive cortisol secretion, promoting a state of neuroendocrine equilibrium and enhanced physiological homeostasis].

As shown in Figure 1, by invoking a relaxation response, both PMR and MBSR likely temper the HPA axis hyperactivity, potentially mitigating these adverse outcomes. This regulation may occur through the enhancement of inhibitory feedback mechanisms or by fostering resilience to stressors, thereby attenuating HPA axis overactivity and excessive cortisol secretion. Supporting this assertion, a randomized controlled trial involving older adults with stress disorders and cognitive complaints revealed noteworthy improvements following a mindfulness-based stress reduction (MBSR) intervention (ClinicalTrials.gov identifier: NCT01693874) (Wetherell et al., 2017). Participants demonstrated enhanced memory performance and improved clinical outcomes, such as reduced excessive worry and depression, which may imply the enhancement of inhibitory feedback mechanisms or resilience to stressors. Importantly, the MBSR group showed a more significant decrease in peak salivary cortisol, a robust indicator of HPA axis activity, especially in those with high baseline cortisol. These findings corroborate the potential of mindfulness interventions like MBSR in attenuating HPA axis hyperactivity, mitigating excessive cortisol secretion, and fostering neuroendocrine equilibrium. Thus, the study substantiates the beneficial role of mindfulness techniques in enhancing physiological homeostasis and overall wellbeing.

Furthermore, PMR's role in medical education can also be theoretically underpinned by the *Information Processing Theory* (Xiong and Proctor, 2018), which emphasizes the sequential and systematic nature of processing stimuli. Stress and muscular tension can disrupt this process, leading to inefficient encoding and storage of information. PMR, by reducing muscle tension and consequently stress, may thus promote the more effective operation of these cognitive processes, allowing for enhanced retention and recall of medical knowledge. Additionally, the *Transactional Model of Stress and Coping* provides a framework for understanding how PMR can aid medical students in managing the stressors endemic to their rigorous training (Obbarius et al., 2021). By providing a means to actively cope with stress through physiological self-regulation, PMR can prevent the appraisal of a stressor as overwhelming, thereby fostering more adaptive learning behaviors and preventing burnout. Likewise, drawing from the *Yerkes-Dodson Law* (Yerkes and Dodson, 1908), which elucidates a curvilinear relationship between arousal and performance, PMR can be seen as a tool to modulate arousal to an optimal level, enhancing performance and learning. This law suggests that there is an optimal level of arousal for peak performance, and excess stress can move an individual away from this peak. PMR helps maintain arousal at this optimal level, particularly beneficial in the high-stakes environment of medical training. Therefore, PMR, through its physiologically grounding techniques, aligns with several psychological and neurobiological theories to potentially mirror the aforementioned stress-mitigating and learning-enhancing effects of MBSR in medical education.

Moreover, PMR posits several key advantages over MBSR. Firstly, its simplicity and ease of learning render it an accessible method for medical students with limited experience in stress-reduction techniques. By providing clear instructions and focusing on tangible physical sensations, PMR eliminates barriers to entry and offers a straightforward approach to relaxation.

Secondly, PMR's immediate effects are particularly valuable in the high-pressure environment of undergraduate medical education. In the face of demanding academic and clinical responsibilities, students can quickly employ PMR as a practical coping mechanism, achieving rapid stress relief without sacrificing valuable study or clinical time.

Thirdly, PMR's limited time commitment makes it especially suitable for the hectic schedules of medical students. A typical PMR session takes a mere 10–20 min to complete, thereby facilitating seamless incorporation into students' daily routines without encroaching on their academic or personal obligations.

In the context of medical education, PMR has been shown to be beneficial. A study conducted on nursing students showed that PMR was effective in reducing test anxiety (Zargarzadeh and Shirazi, 2014). Another study found that a course introducing Autogenic Training (AT) and PMR led to a significant reduction of burnout and anxiety within the participating group of medical students (Wild et al., 2014). Furthermore, a study on nursing students taking their initial clinical training found that PMR improved emotional and physical health (Alhawatmeh et al., 2022). PMR has also been found to foster resilience in healthcare professionals. A study on nurses caring for COVID-19 patients found that PMR significantly diminished stress and anxiety levels after intervention (Ganjeali et al., 2022).

The practice of PMR does necessitate a quiet and comfortable space. However, this requirement can be reasonably accommodated within a medical education setting. Institutions can designate dedicated spaces for relaxation exercises or encourage students to practice PMR during breaks or in their dormitories. Furthermore, while PMR's long-term benefits may be somewhat circumscribed compared to those of MBSR, the immediacy of its effects compensates for this limitation, providing students with an invaluable tool for managing acute stressors. Considering the discernible advantages—notably its straightforward application, swift onset of benefits, and relatively low time investment—we adopted PMR as the stress reduction technique of choice within our medical student curriculum.

## 2 Methods

### 2.1 Study landscape and context

**Figure 2:**
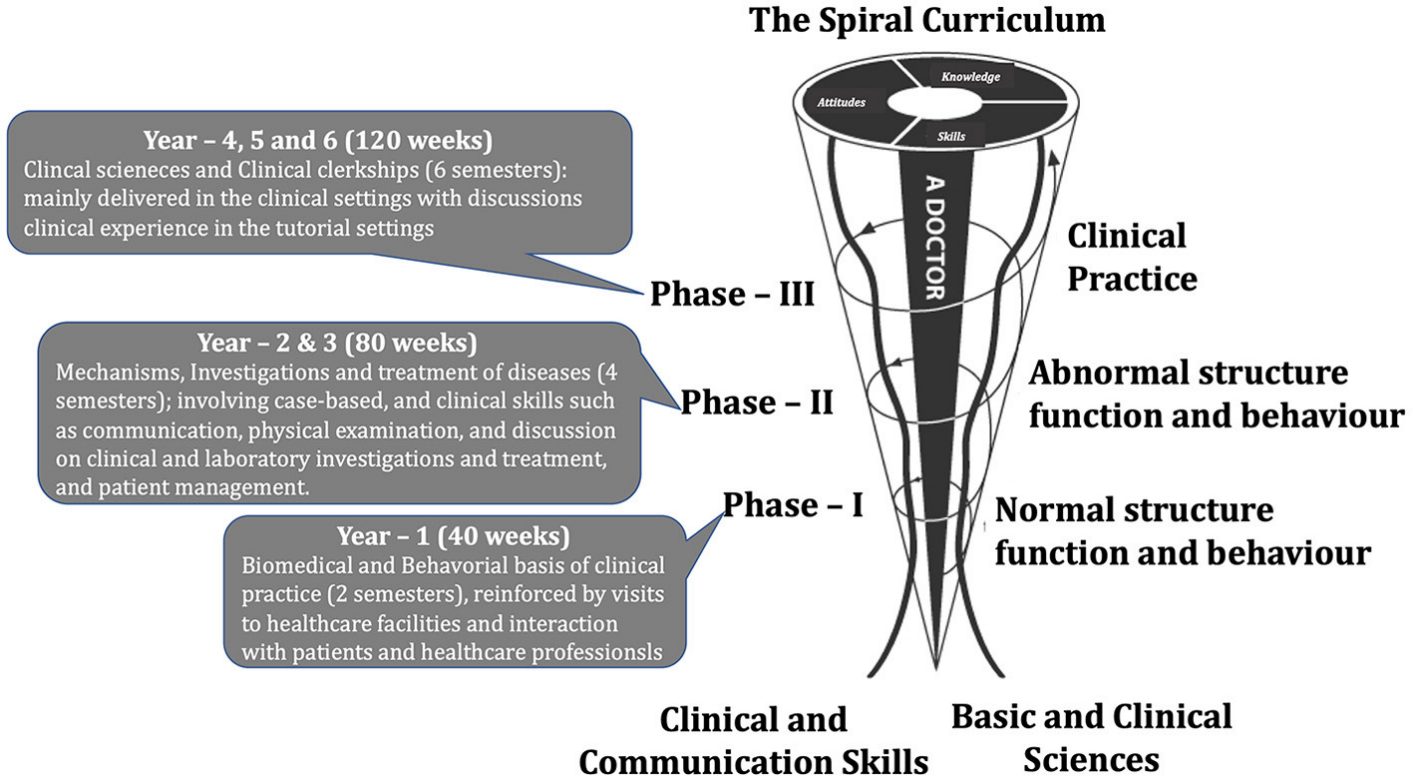
Depiction of the 6-year undergraduate medical pedagogical schema at Mohammed Bin Rashid University of Medicine and Health Sciences (MBRU). The curriculum is categorized into three successive phases. Each phase represents an integral component of a “spiral curriculum”, characterized by iterative exploration of topics, with escalating complexity and in-depth understanding in subsequent cycles.

The CBMC at Mohammed Bin Rashid University of Medicine and Health Sciences (MBRU) features a three-part structure. Each phase is closely linked and builds upon the one before it. This creates a spiral architecture where students revisit subject matter iteratively. With each cycle, they build on the foundational concepts previously introduced (Figure 2) (Banerjee et al., 2018; Naidoo et al., 2021). MBRU's medical school is home to a diverse body of students. These individuals come from more than 19 different countries and have been educated in 20 distinct high school curricula. Of the student demographic, females make up roughly 74%—that's 191 out of 258 students.

### 2.2 Participant recruitment and eligibility criteria

For our study, participant recruitment focused on meeting set inclusion criteria. We required that participants be enrolled in MBRU's undergraduate MBBS curriculum. Participation needed to be voluntary, with individuals giving verbal consent before the commencement of the program. Additionally, we requested that prospective participants disclose any significant neuropsychological conditions they might have. This recruitment period extended over 2 weeks and was facilitated by an electronic flier. This flier was carefully tailored to reflect the institution's sociocultural ethos and was circulated to all student cohorts via institutional email. We recruited a total of 20 participants on a first-come, first-served basis to engage in the PMR program. The group of 20 ensured a roughly even representation from each of the three phases of the MBBS curriculum (Figure 2). In adherence to institutional social distancing protocols mandated by the COVID-19 pandemic, the number of participants was intentionally limited to 20.

### 2.3 Instructional design and rationale: selecting Gagne's instructional design framework for developing PMR program

In developing the PMR program within our medical curriculum, we selected Gagne's Nine Events of Instruction as our foundational instructional design model (Tambi et al., 2018). This choice is substantiated by Gagne's alignment with behavioral learning principles, offering a structured yet flexible approach crucial for the nuanced acquisition of PMR skills. Our confidence in Gagne's model is bolstered by previous successful implementations, as detailed by Tambi et al. (2018), and Naidoo et al. (2020), which showcased the model's utility in managing the cognitive load during the learning process—a core tenet supported by Cognitive Load Theory.

Moreover, Gagne's model provides a systematic framework that enhances learning by building on the students' existing knowledge base, thus facilitating deeper understanding. It also encourages active engagement with the learning material, a key aspect of the constructivist approach, which is essential in the practice of PMR. Each step in Gagne's model serves as a precursor to the next, fostering a scaffolded learning experience that reinforces previous knowledge while introducing new concepts—this incremental approach is echoed in the hierarchical nature of learning suggested by Bloom's Taxonomy.

Furthermore, the PMR program development under Gagne's guidance leverages his model's metacognitive element, prompting students to reflect upon and regulate their learning strategies. This aspect draws from Flavell's Metacognitive Theory, which is instrumental for students to develop self-regulated learning competencies, especially pertinent in the self-awareness centric practice of PMR.

Our decision to use Gagne's model over others also considers the diversity of learning styles in medical education. Gagne's approach, with its multisensory engagement, is compatible with Mayer's Cognitive Theory of Multimedia Learning (Castanelli, 2023), ensuring that the pedagogical strategy encompasses visual, auditory, and kinesthetic elements. This aligns with contemporary understandings of effective learning, where information retention is maximized through the engagement of multiple senses.

Likewise, the motivation and emotional dimensions of learning are not sidelined in Gagne's model; it integrates these through its attention to arousing student interest and maintaining attention—a principle reflected in Keller's ARCS Model of Motivational Design (Luo et al., 2022). This is crucial in the context of medical education, where the cultivation of intrinsic motivation can significantly impact students' learning efficacy. Furthermore, in medical education, Gagne's model demonstrates distinctive strengths, enhancing learning outcomes by breaking down complex medical concepts into digestible components, emphasizing prerequisite knowledge, and promoting active learning through practice and feedback integration (Azar et al., 2021). These aspects contribute to the development of clinical reasoning and decision-making skills (Buscombe, 2013), culminating in proficient and confident medical practitioners, as evidenced by the successful implementation of the PMR program and our previous experiences with the model.

Building on the established benefits of Gagne's model in enhancing medical education, it becomes imperative to contrast it with other instructional design frameworks to further elucidate its suitability for the structured learning pathways required in PMR training. When delineating the merits of Gagne's instructional design model in the context of PMR training, it is instructive to draw a comparative analysis with another well-regarded framework in educational theory—the Bruner's Theory of Instruction (Biddinger, 1993). Both models emphasize the significance of structuring material in a manner conducive to learning, yet they diverge in their approach to the sequence and complexity of content delivery.

Gagne's model is systematic, proposing nine definitive steps that facilitate progressive learning. This methodical structure is particularly advantageous for PMR instruction, where, as elaborated above, each skill set builds directly upon the preceding one, ensuring a clear trajectory from novice to mastery within the medical student's learning pathway. The hierarchical nature of Gagne's model corresponds with the incremental complexity of medical procedures, allowing students to establish a solid foundation before advancing to more intricate aspects of PMR.

Conversely, Bruner's Theory posits that learning can happen in any order as long as it is in a context that makes sense to the learner. This spiraling curriculum suggests that students revisit topics repeatedly over time with increasing complexity, which, while beneficial for reinforcing learning, may not provide the structured scaffold necessary for the sequential mastery required in PMR techniques. Gagne's approach, therefore, edges out Bruner's in the context of PMR due to its linear progression, which aligns better with the step-by-step nature of learning relaxation techniques.

Moreover, Gagne's focus on external conditions of learning, such as providing cues and facilitating performance through guidance, is well-suited to the hands-on, demonstrative nature of PMR training. It ensures that learners are not only cognitively engaged but also practically involved in the learning process. In contrast, Bruner's model, with its strong emphasis on discovery learning, encourages learners to construct their knowledge through exploration and inquiry, an approach that may not align as closely with the methodical skill acquisition required in PMR.

In summary, while both Gagne and Bruner provide robust frameworks for educational instruction, Gagne's model offers a structured, sequenced approach that is particularly aligned with the methodical nature of PMR learning in medical education. This structured approach is crucial for ensuring that medical students acquire the PMR techniques in a manner that promotes confidence, competence, and ultimately, the capacity to apply these skills effectively in their future clinical practice.

### 2.4 PMR program implementation

The 20 participants recruited for the PMR program arrived at the study site 30 min before the program's initiation. On arrival they were greeted by a trained instructor (BN) with over a decade of PMR practice experience. After providing informed consent, participants attended PMR sessions held in the morning between 9:00 and 11:00 a.m. during the fall semester. They were instructed to abstain from alcohol, nicotine, and caffeine consumption 12 h before participation to ensure that the results were not confounded by the acute physiological and psychological effects of these substances, which could potentially alter cognitive performance, mood, or biometric readings. The PMR program site was thoroughly disinfected, and participants were seated following COVID-19 social distancing protocols. Classical piano recitals, such as Chopin's Nocturnes, Debussy's Clair de Lune, and Satie's Gymnopédies, served as background music during the sessions to create a soothing and controlled auditory environment, potentially facilitating relaxation and concentration while also ensuring a consistent and pleasant stimulus across all experimental conditions.

Before the program began, BN supplied a script detailing PMR's overview and proper practice techniques (refer to Supplementary material) to promote compliance when participants practiced independently. The program comprised five sessions (2 h per session) over 5 weeks, with participants encouraged to practice PMR daily, ideally at day's end, to promote the development of a relaxation routine, enhance the potential for better sleep quality, and provide a consistent time frame that aligns with the natural winding down of the body's circadian rhythm.

### 2.5 Focus group methodology

Upon the successful completion of the PMR program, a methodological approach was employed to gather in-depth feedback, which involved conducting two focus group sessions, each engaging 10 participants (Protudjer et al., 2023). These sessions were meticulously designed within the scaffolding of social constructionist principles, affirming that understanding is co-created through social interaction—a concept well-suited to the dynamics of focus groups (Clarke, 1999). The facilitated discussions within the focus groups were deeply rooted in symbolic interactionism, which acknowledges the significance of shared symbols and meanings derived from the PMR program (Then et al., 2014). The reciprocal communication encouraged by this framework enhanced the exchange of individual perceptions, resonating with the theories of group behavior that underscore the importance of interactive dialogue in learning and reflection (Rodriguez et al., 2020). Employing communication theory as a guiding construct, the focus groups were structured to promote an expressive and insightful exchange of experiences, enriching the qualitative data with detailed narratives (Clarke, 1999). This methodological choice allowed for a detailed exploration of the participants' communicative processes and the shared construction of their experiences with the PMR program. By adhering to a pragmatic approach within these discussions, the sessions served as a conduit for elucidating the experiential knowledge of participants, reflecting the pragmatic values of actionable and experience-based knowledge (Then et al., 2014). The rich qualitative data garnered from the focus group dialogues provided a critical lens through which the program's impact could be assessed. In essence, these focus group sessions were a deliberate application of psychological and educational theories to capture the cognitive and affective responses of medical students to the PMR program. Through the strategic use of these theoretical frameworks, the focus groups emerged as an invaluable tool for evaluating the program's effectiveness and pinpointing potent areas for further educational refinement.

An investigator possessing substantial proficiency in socio-behavioral research domains, and who maintained an external position to the PMR program's developmental procedure, undertook the role of moderator for these sessions. Participation in these sessions was entirely voluntary and predicated on the attainment of verbal consent from each participant prior to the initiation of the focus group proceedings. The nature of data procurement during this phase was predominantly exploratory, aiming to gather a broad range of information and insights without a predefined hypothesis, allowing for the identification of patterns, relationships, and phenomena (Konopka et al., 2018), thereby fostering an environment conducive to encouraging participants to elucidate their thoughts, feelings, and impressions concerning their individual experiences in the context of the PMR program. The moderator posed broad, generative queries to the group. This approach was taken to stimulate and catalyze participant responses. The ultimate goal was to cultivate a robust dialogue. By doing so, it was intended to maximize the diversity and depth of the discourse among participants. Each interactive focus group dialogue was designed to span an approximate duration of 1 h. This timeframe was chosen to allow ample opportunity for discussion without causing fatigue, ensuring a focused and engaged participation throughout.

### 2.6 Data analysis frameworks

#### 2.6.1 Assessment of emergent themes from focus group sessions: employing grounded theory for theoretical framework development

The participant-generated perceptions, capturing their subjective experiences and insights, were meticulously transcribed verbatim. These transcripts became the foundational data for analysis. Adhering to grounded theory methodology (Glaser and Strauss, 1967), two coders, steeped in the discipline of qualitative research, independently engaged in the analytical process. Grounded theory, a rigorous qualitative research method that underscores inductive reasoning, was particularly apt for our exploration. It eschews starting with a theoretical framework, instead privileging the data to guide theory development.

Furthering the process of analysis, the coders immersed themselves in the transcripts, allowing the data to speak. With a keen eye for patterns and nuances, they embarked on the journey of open coding, labeling phenomena without preconceptions. This initial phase was followed by axial coding, where identified categories were related to subcategories, hypotheses formed, and connections established, providing a scaffold for the emerging theoretical structure. The final stage, selective coding, involved weaving the disparate thematic strands into a cohesive theoretical tapestry, elucidating the core phenomena underpinning participant experiences with the PMR program.

Throughout this meticulous analytical expedition, coders maintained a reflexive stance, aware of the interpretive nature of their role. The grounded theory analysis was not only a method but a methodology, embodying a set of principles and practices that advocate for the credibility and dependability of the findings. The interactive process of coding, categorization, and comparison rendered a dynamic and comprehensive understanding of the data, culminating in a grounded, substantive theory that offered both descriptive and explanatory power. This in-depth exploration illuminated the psychological landscape of the participants, providing valuable insights into their engagement with PMR and adding a rich layer to the existing body of knowledge within the psychological community.

#### 2.6.2 Thematic analysis and synthesis: independent coding and collaborative refinement in grounded theory

In the nascent stage of coding, a pivotal step was taken to ensure objectivity: one member of the research team, deliberately chosen for their lack of involvement in the program's development and dissemination, undertook an independent examination of the data. Utilizing content analysis, this researcher systematically sifted through the transcribed perceptions, meticulously pinpointing properties that signified key themes and concepts resonating among the study participants. This methodical detachment was critical, serving to mitigate bias and enhance the integrity of the data interpretation.

Parallel to this, a second coder with extensive experience in qualitative research embarked on an independent coding journey. This researcher's seasoned expertise provided a profound layer of analysis to the transcribed perceptions. The juxtaposition of a fresh perspective with seasoned analytical acumen ensured a comprehensive exploration of the data.

The subsequent phase was marked by an iterative, collaborative effort between the coders. Multiple meetings were convened, serving as fertile ground for discourse and reconciliation of any disparities in their analytical perspectives. It was within these iterative discussions that the themes began to be refined and finalized.

To further solidify and articulate the convergence of themes, memoranda were meticulously composed. These written reflections were not mere annotations; they were interpretive passages that illuminated the shared experiences and perceptions of the participants post-PMR program. These memoranda acted as a narrative rationale, providing transparency to the analytical process, and encapsulating the essence of the emergent themes. This rigorous approach not only fortified the validity of the identified themes but also enriched the depth and breadth of the theoretical insights gleaned from the participant data.

#### 2.6.3 Systematic coding and theme identification using Braun and Clarke thematic analysis framework

Utilizing the Braun and Clarke thematic analysis framework, we conducted observational coding to examine the data (Braun and Clarke, 2014; Ayre and McCaffery, 2022; Das, 2022). A key advantage of utilizing Braun and Clarke's thematic analysis within the context of medical education and psychological research is its agnostic stance toward theoretical and epistemological underpinnings. Unlike other analytic frameworks such as phenomenology, narrative analysis etc., that often require adherence to specific theoretical lineages or presuppose certain epistemological commitments, Braun and Clarke's methodology offers researchers the latitude to engage with the data in a more unrestricted manner. This flexibility is paramount in multidisciplinary fields where various schools of thought converge, necessitating an approach that is not bound by the constraints of a single theoretical orthodoxy. Also, Braun and Clarke's approach entails a meticulous and systematic engagement with the dataset, mandating an iterative process of sifting through each data item—whether it be interview-transcripts, observation notes, or other qualitative materials—with an assiduous eye for detail. This enables the identification of salient patterns that may not be immediately apparent, thus permitting a nuanced analysis that transcends mere surface-level inspection. Furthermore, through its structured yet adaptable framework, Braun and Clarke's thematic analysis allows for a nuanced dissection and interpretation of complex phenomena, which is critical in the realm of medical education and psychology where interpretive precision is as valuable as empirical observation. The approach facilitates the extraction of themes that are not only descriptive but also carry interpretive heft, offering a comprehensive insight into the psychological and behavioral dynamics of study participants. By affording the opportunity to unearth and articulate both overt and latent themes within the data, Braun and Clarke's method is particularly advantageous for delineating the intricacies of participant experiences in a way that is both methodologically rigorous and richly informative, thereby contributing to the advancement of knowledge in these complex fields.

#### 2.6.4 Thematic analysis of focus group data

As indicated above, for the coding process we adopted Braun and Clarke's thematic analysis framework, which unfolds over six distinct phases. Each phase, as applied to our study, is delineated below to illustrate our methodological approach.

1. **Familiarization with the Data:** Initially, our research team diligently reviewed the interview-transcripts from the focus group sessions. This immersive engagement with the data allowed us to identify preliminary patterns and garner an overarching sense of the participants' shared experiences with the PMR program.
2. **Generating Initial Codes**: We then proceeded to generate concise, pertinent codes that captured the essence of the participants' feedback. This was achieved through an inductive coding process, which remained grounded in the actual data rather than preconceived theories, thus ensuring an authentic representation of participant narratives.
3. **Searching for Themes**: Subsequently, the initial codes were examined and clustered into potential themes. This step was guided by a blend of psychological learning theories and social interaction principles, considering the data within the context of educational experiences and the collective learning environment fostered by the PMR program.
4. **Reviewing Themes**: Each emergent theme was rigorously reviewed and refined to ascertain its validity across the data set. The themes were critiqued in light of constructivist theories, which recognize knowledge as a construct developed through individual experiences and social interactions, thus reinforcing the relevance and resonance of the themes with the participants' experiences.
5. **Defining and Naming Themes:** The defined themes were then named and further elaborated upon, with each theme capturing a unique aspect of the students' journey through the PMR program. This phase harnessed the principles of experiential learning theory, acknowledging the personal transformation through direct experience that is integral to adult learning.
6. **Producing the Final Report**: Finally, we meticulously composed a report that articulated the themes and interwove participant quotations to exemplify and validate our findings. The report not only summarized the thematic insights but also contextualized them within the broader discourse of stress management in medical education.

Throughout this analytical journey, we maintained a reflexive stance, aware of our potential biases as researchers and educators. By applying Braun and Clarke's framework with rigor and sensitivity, we distilled the essence of the medical students' experiences with the PMR program, laying the groundwork for future instructional enhancements and providing an evidence-based approach to stress reduction in the high-pressure environment of medical education.

### 2.7 Quality appraisal and framework alignment

#### 2.7.1 Ensuring rigor and trustworthiness: quality appraisal using O'Brien's SRQR

In this pioneering investigation, we assessed the study's quality using O'Brien's Standards for Reporting Qualitative Research (SRQR) framework to ensure the rigor and trustworthiness of our findings (O'brien et al., 2014). The SRQR is a comprehensive framework that offers guidelines for reporting qualitative research, enhancing the transparency, consistency, and comprehensibility of the reported findings. The SRQR consists of a checklist of 21 items that are considered essential for a high-quality qualitative research report (Table 2). The goal of the SRQR is to provide a framework for authors to comprehensively report their qualitative research methods and findings, thus enabling readers to critically appraise and interpret the research's validity and reliability. This standardization also helps to make the research more accessible to a wider audience and to facilitate the inclusion of qualitative studies in systematic reviews.

The choice of employing O'Brien's SRQR over other frameworks, such as the Consolidated Criteria for Reporting Qualitative Research (COREQ) (Tong et al., 2007) or the Qualitative Research Review Guidelines (RATS) (Anderson, 2010), stems from its robust and detailed criteria, specifically tailored to address the unique characteristics and requirements of qualitative research. While COREQ focuses primarily on reporting interview and focus group studies, and RATS is primarily concerned with the appraisal of manuscripts submitted to BioMed Central journals, both of these alternative frameworks may not be as comprehensive or relevant to the current study, given their narrower scope or context-specific focus.

Evaluating the quality of this study is crucial, as it strengthens the credibility and validity of its outcomes. This process ensures that the results are reliable and significant. Additionally, applying O'Brien's SRQR for quality assessment aids in replicating and extending this study in future research. Such efforts contribute to a deeper understanding of the PMR program's effectiveness and possible enhancements.

#### 2.7.2 Alignment of PMR program outcomes with competency frameworks in medical education and clinical training

A competency framework is a structured model that outlines the specific abilities, knowledge, skills, and behaviors required for effective performance within a professional role or organization (Zumstein-Shaha and Grace, 2023). It serves as a foundational guide for both educational curricula and professional development, ensuring that individuals are equipped to meet the demands and challenges of their work. The importance of competency frameworks in medical education (Palermo et al., 2022), clinical practice, and patient care lies in their ability to define the essential knowledge, skills, and attitudes that healthcare professionals must possess to provide safe and effective care. Alignment with recognized competency frameworks ensures that educational programs are tailored to address the needs of the medical community and foster the development of well-rounded practitioners.

**Figure 3:**
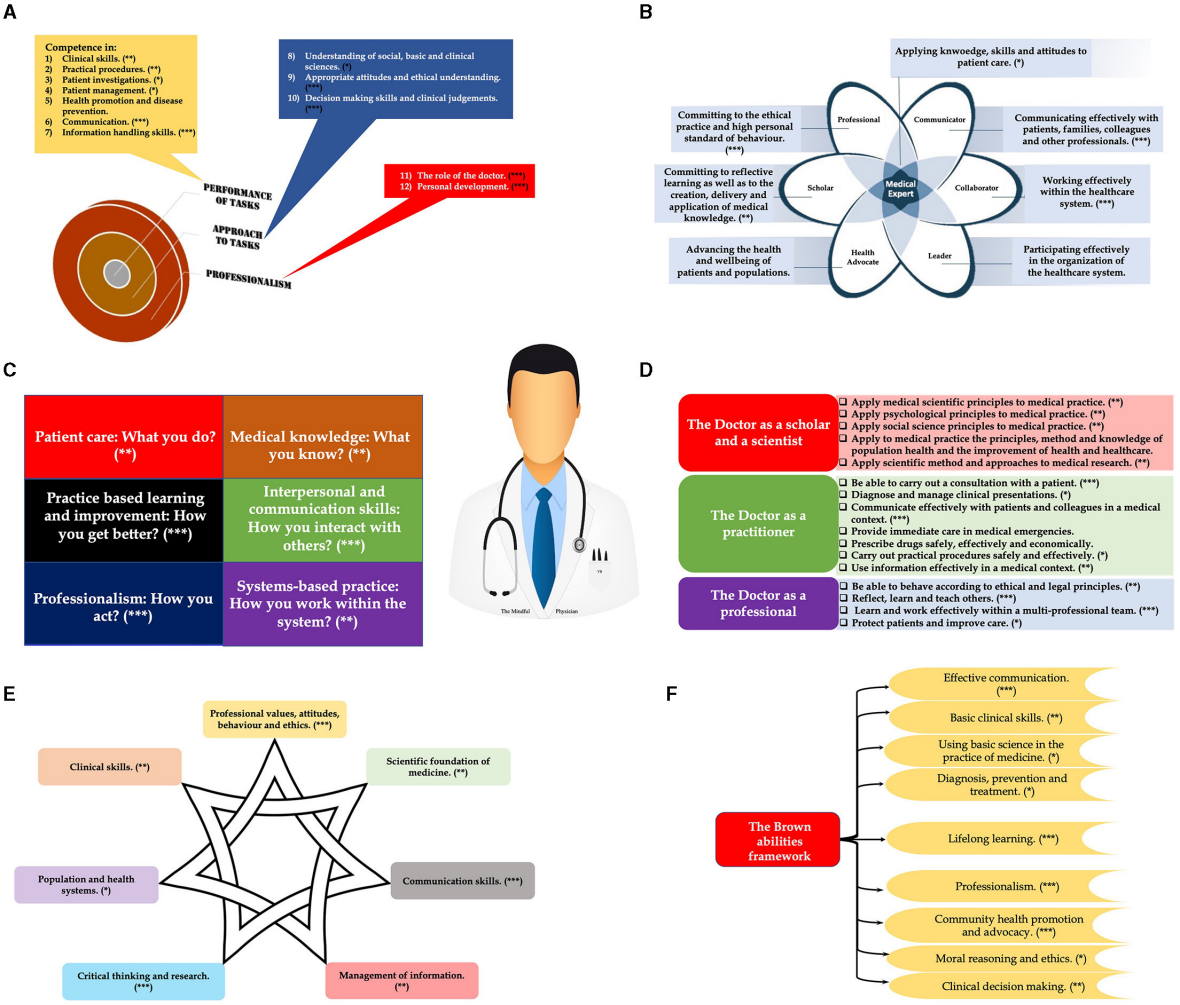
Alignment of Progressive Muscle Relaxation (PMR) with Key Domains of Various Competency Frameworks. This figure illustrates the congruence of PMR, through its influence on self-control, self-realization, liberation, awareness, and interpersonal relationships, with several pivotal domains of: **(A)** the Scottish Doctor Framework; **(B)** the CanMEDS Physician Competency Framework; **(C)** the Accreditation Council for Graduate Medical Education (ACGME) Competency Framework; **(D)** the General Medical Council (GMC) UK Competency Framework; **(E)** the Global Minimum Essential Requirements (GMER) Competency Framework; and **(F)** the Brown Abilities Competency Framework. PMR, by augmenting these key traits, aligns well with the competencies outlined in these frameworks, thereby emphasizing its potential contribution to holistic medical education and practice (***contributes significantly to the competence or domain; **contributes mostly to the competence or domain; *contributes to some extent to the competence or domain).

In this study, we assessed the versatility and relevance of the PMR program (aimed at increasing mindfulness, reducing stress, and augmenting resilience) by aligning the program's outcomes with multiple established competency frameworks (see a to f below) (Figure 3). Conducting this alignment step is crucial for the current study as it demonstrates the applicability of the PMR program across various contexts and jurisdictions, reinforcing its potential to contribute to the holistic development of medical professionals.

a) The Scottish Doctor Framework (Ellaway et al., 2007) (Figure 3A) outlines the outcomes and standards for undergraduate medical education in Scotland. It emphasizes the importance of resilience as a key attribute for medical students to cope with the demanding nature of their studies and clinical training, fostering their ability to adapt to various situations and manage stress effectively. This framework's significance extends beyond academic rigor; it is instrumental in preparing future physicians to thrive in a fast-paced, often unpredictable healthcare landscape, ultimately enhancing the resilience and sustainability of the medical profession itself.
b) The CanMEDS Physician Competency Framework (Thoma et al., 2023) (Figure 3B), developed by the Royal College of Physicians and Surgeons of Canada, focuses on the essential competencies expected of medical graduates. Resilience is recognized as a vital aspect of the “Professional” role, enabling physicians to maintain their wellbeing and perform effectively under challenging conditions, ultimately contributing to better patient care. Incorporating tenets of Canadian healthcare, the framework underscores the importance of resilience not only for individual practitioners but also for ensuring a sustainable, responsive healthcare system that aligns with Canada's values of equitable and accessible patient care.
c) The Accreditation Council for Graduate Medical Education (ACGME) (Nasca et al., 2012) (Figure 3C), establishes standards for graduate medical education in the United States. Resilience is seen as an essential component in the “Professionalism” and “Systems-Based Practice” domains, supporting physicians in managing stress, maintaining wellbeing, and effectively adapting to changing clinical environments and systems. This emphasis on resilience is crucial in enabling healthcare professionals to navigate the complexities of the U.S. healthcare system, which demands not only clinical expertise but also the ability to operate within and contribute to the improvement of healthcare delivery and patient safety.
d) The General Medical Council (GMC) UK Competency Framework (Southgate et al., 2001) (Figure 3D) sets the standards for medical education and practice in the UK. Resilience is highlighted as an essential component within the framework, as it enables healthcare professionals to cope with the challenges of their profession, leading to improved patient care and safety. This focus on resilience facilitates not only personal wellbeing but also ensures a sustainable and responsive healthcare system, which is essential for the delivery of high-quality patient care and safety across the UK's diverse healthcare settings.
e) The Global Minimum Essential Requirements (GMER) Competency Framework (Core Committee, 2002) (Figure 3E), aims to provide a set of core competencies that all medical graduates should possess, regardless of their geographical location. Resilience is considered an essential aspect of this framework, ensuring that healthcare professionals are prepared to face the diverse challenges of global medical practice. This emphasis on resilience underscores the importance of adaptable, robust medical practitioners who can sustain the rigors of healthcare delivery across varied and often unpredictable environments, ultimately fostering a more resilient global healthcare workforce.
f) The Brown Abilities Competency Framework is a comprehensive framework (Batt et al., 2021) (Figure 3F) encompassing the essential competencies required for medical professionals. Resilience is integrated into various domains of the framework, emphasizing its importance in cultivating well-rounded, adaptive practitioners capable of providing quality patient care. The framework's incorporation of resilience accentuates the necessity for medical practitioners to develop robust personal resources, enabling them to deliver high-quality patient care in the face of clinical challenges and stressors.

Drawing upon principles from learning, psychological, and social theories, it becomes evident how the PMR program aligns with esteemed competency frameworks to foster critical competencies in psychology and medical education. Through the lens of social constructivism, the program encourages collaborative learning and reflection, thus enhancing effective communication and empathy. Cognitive theory's emphasis on mental processes underpins the development of critical thinking skills, while the principles of adaptive learning promote flexibility and adaptability in complex clinical scenarios. This multidimensional approach not only nurtures the individual's psychological resilience but also equips future healthcare professionals with a holistic skill set indispensable for superior patient care and professional excellence.

#### 2.7.3 Theoretical perspectives: Bourdieu's Theory of Practice

The integration of PMR into the undergraduate medical curriculum is examined through the theoretical framework of Pierre Bourdieu's Theory of Practice (Rhynas, 2005). Bourdieu's sociological theory provides a tripartite construct—*habitus, capital, and field*—which serves as a methodological tool for analyzing how the PMR program may shape medical students' professional development and practice.

In medical education, the concept of ***habitus*** is pivotal. It refers to the ingrained dispositions of medical professionals, shaped by their unique social and cultural experiences. For example, a physician's approach to patient care often reflects the values and practices absorbed during their training and practice environment.

***Capital***, in this context, includes resources like knowledge, professional networks, and reputation. These assets enable medical professionals to navigate and excel in healthcare settings. A surgeon's technical expertise (cultural capital) and connections within the medical community (social capital) are key examples.

Lastly, the ***field*** in medical education represents the various environments where professionals interact and compete. This could be a hospital, a clinic, or an academic institution. Here, individuals strive to accumulate and leverage their capital. For example, an academic researcher competes for recognition and funding within the scientific community, a distinct field with its own rules and rewards.

Applying Bourdieu's Theory of Practice to the integration of the PMR program in undergraduate medical education allowed us to assess the ways in which this program can influence students' habitus, enhance their capital, and ultimately improve their performance within the field of medicine.

#### 2.7.4 Rationale for choosing Bourdieu's Theory of Practice

1. **Alignment with Medical Education Context**: Bourdieu's Theory of Practice, with its emphasis on habitus, capital, and field, provides a comprehensive framework to understand how medical students assimilate and apply the PMR program in their professional development. The theory's focus on the interplay between individual dispositions (habitus), resources (capital), and the social environment (field) aligns well with the dynamics of medical education and professional practice.
2. **Habitus and Professional Identity Formation**: in medical education, habitus can be seen as the ingrained attitudes and behaviors that students develop. This concept is crucial in understanding how students internalize the skills and ethos of the PMR program, shaping their professional identity and practice.
3. **Capital—**Acquiring and Utilizing Resources: Bourdieu's concept of capital, encompassing knowledge, skills, and professional networks, is particularly relevant for medical students. The PMR program equips them with unique skills (cultural capital) and potentially expands their professional networks and reputation (social capital).
4. **Field—**Understanding the Medical Profession: the field in Bourdieu's theory represents the various settings in which medical professionals operate. By applying this concept, we can analyze how the PMR program prepares students to navigate and excel in different medical fields, from hospitals to research institutions.

#### 2.7.5 Comparison with other theories

**1. Vygotsky's Sociocultural Theory (Vasileva and Balyasnikova**, **2019****):**

- ***Limitation*:** Vygotsky's theory emphasizes the role of social interaction and cultural context in learning. While it could offer insights into the collaborative aspects of learning PMR, it lacks the comprehensive framework provided by Bourdieu in terms of professional identity formation and the utilization of educational experiences in various professional contexts.

**2. Kolb's Experiential Learning Theory (Kong**, **2021****):**

- ***Limitation:*** Kolb's theory, focusing on the learning cycle of experience, reflection, conceptualization, and experimentation, could explain how students internalize and apply PMR skills. However, it does not address the broader social and professional dimensions of medical training that Bourdieu's theory encapsulates.

**3. Bandura's Social Learning Theory (Galanaki and Malafantis**, **2022****):**

- ***Limitation:*** Bandura's theory, which emphasizes learning through observation and modeling, could be relevant for understanding how students learn PMR techniques by observing instructors. However, it does not sufficiently account for the complex interplay of personal dispositions, social structures, and resources in professional settings, which is critical in medical education.

**4. Mezirow's Transformative Learning Theory (Eschenbacher and Fleming**, **2020****):**

- ***Limitation:*** Mezirow's theory focuses on how individuals change their perspectives through critical reflection, which could be applicable to learning PMR. However, it primarily addresses personal transformation rather than the professional development and integration into the medical field, which is a key aspect of Bourdieu's theory.

#### 2.7.6 Conclusion

Bourdieu's Theory of Practice was chosen for its comprehensive ability to analyze and interpret the integration of the PMR program into medical education. It addresses not only the individual learning and adaptation processes but also the broader social and professional contexts in which these skills are applied. This makes it more suitable compared to other theories which may focus more narrowly on individual learning processes or lack the depth in addressing the professional application in the field of medicine.

## 3 Results

### 3.1 Implementation of Gagne's 9-steps: tailoring the PMR program for medical education

The steps of the designed PMR program were tailored and aligned with Gagne's 9-steps of instructional design (see Table 1). An elaboration of the specific procedural steps is detailed in the following sections.

**Table 1:** Steps of PMR program steps aligned to Gagne's model.

**Step**	**Key event (allocated time)**	**PMR program steps**
1	Gain attention (5 min)	Participants will be provided with an audio cue (i.e., relaxing music) to gather around the PMR facilitator.
2	Inform participant of the objective (10 min)	The participants will be provided with a handout of supplementary information describing the objective of the PMR session, along with the step-by-step guide plan.
3	Stimulate recall of prior learning (25 min)	A YouTube video, circulated earlier to the participants, will be rescreened during which the facilitator will elaborate on the benefits of PMR and the rationale behind individual steps. YouTube video: (https://www.youtube.com/watch?v=86HUcX8ZtAk)
4	Present content material (40 min)	Using the handout circulated to the participants in Step 2, the facilitator will demonstrate the individual steps of PMR. In this step, participants will be invited by the facilitator to clarify any doubts/questions regarding the individual steps of PMR.
5	Provide learning guidance (10 min)	The PMR facilitator will elaborate on the rationale behind each step; however, the focus will also be directed to the specific dos and don'ts pertaining to each step of the program. Case in point, the participant will be informed that if she/he experiences any pain or discomfort at any of the targeted muscle groups, the participant should feel free to omit that step.
6	Elicit performance (30 min)	Participants, with cues from the PMR facilitator, will perform the individual steps of the activity, details of which were circulated earlier in step 2 (Supplementary material).
7	Provide informative feedback (15 min)	The PMR facilitator will informally collect reflections from the participants using a framework in line with that of Pendleton's feedback model that is: a) Check that the participants want and are ready for feedback. b) Invite the participants to comment on the Supplementary material. c) Allow participants to state what was done well. d) Allow the observer/s to state what was done well. e) Encourage participants to state what could be improved. f) Encourage participants to state how it could be improved. g) Ensure that an action plan for improvement is devised.
8	Assess performance (15 min)	In the presence of the PMR facilitator, the participants will review the PMR video, introduced earlier in Step 3, to evaluate their performance against what was shown in the video.
9	Enhance retention and transfer (5 min)	The PMR facilitator will provide the participants with additional resources on the benefits of PMR, will urge them to practice PMR at home and workplace at their convenience, and attend the PMR programs that are organized in the University. Additionally, the participants will be encouraged to reflect on the effect of PMR on their professional and social domains and record their reflections through journaling.

#### 3.1.1 Step 1: capture students' attention

The instructor implemented the “pattern interrupt phenomenon” (to capture students' attention). An auditory cue signaled participants to gather around the PMR facilitator.

#### 3.1.2 Step 2: communicate learning objectives

Additional materials on PMR, provided a week earlier, were reviewed and the main goals of the course were explained by the facilitator. The facilitator also went over a detailed guide on how to perform PMR exercises, and students were informed about the specific learning goals they were expected to achieve by the end of the program.

#### 3.1.3 Step 3: stimulate recall of prior learning

A previously shared YouTube video, demonstrating the PMR technique and explaining its benefits and the reasoning behind each step, was revisited for a more comprehensive understanding. This approach was designed to leverage social learning theory, promoting observation and imitation as a means of reinforcing the learning process.

#### 3.1.4 Step 4: present content material

The facilitator meticulously exhibited each maneuver of the PMR technique as delineated in the previously distributed handout, catering to diverse learning preferences including visual, auditory, and kinesthetic modalities. Throughout this demonstration, participants were actively invited to articulate inquiries or confusions pertaining to the procedure, ensuring a comprehensive understanding of the technique.

#### 3.1.5 Step 5: offer learning guidance

The facilitator elucidated the underlying principles and intentions of each PMR step, highlighting essential guidelines and cautions. Participants were instructed, for instance, to feel at liberty to bypass any step that induced discomfort or pain in the associated muscle groups, thus prioritizing personal safety and comfort.

#### 3.1.6 Step 6: elicit performance

During this step, students actively consolidated their knowledge by engaging in the PMR exercises, dedicating ample time to practice. They were methodically led through the sequence of tensing and then releasing tension in each muscle group, starting from the head, and moving toward the toes. Concurrently, they were coached to monitor their breath, fostering a tranquil and relaxed state of being.

#### 3.1.7 Step 7: provide informative feedback

Employing Pendleton's feedback model (Hardavella et al., 2017), students engaged in peer feedback within their designated groups. The instructor visited each group, providing individual assistance and immediate feedback. This reflective stage aimed to refine students' understanding, enhance self-awareness, and provide a clear trajectory for improvement.

#### 3.1.8 Step 8: assess performance

In the presence of the PMR facilitator, participants rewatched the PMR video from Step 3. This review focused on comparing their own performance with the demonstrated technique. This step fostered critical thinking and self-awareness, aiding in the enhancement of stress reduction skills through the PMR method.

#### 3.1.9 Step 9: enhance retention and transfer

After each practice and reflection session, the PMR facilitator offered extra materials detailing the advantages of PMR. Participants were encouraged to practice both at home and in their workplace. Information about future PMR programs at the university was shared. The facilitator then revisited the learning objectives, addressed any lingering questions, and facilitated discussions on action plans.

### 3.2 Qualitative analysis

The qualitative analysis entailed identifying and interpreting themes that emerged from the data. This process involved carefully examining the qualitative data to uncover patterns and meanings. Five key themes were identified, as detailed below:

#### 3.2.1 Self-control

The theme of ‘self-control' was notably evident, underscoring the participants' proficiency in self-regulation. This involved intentional governance over cognitive processes, affective experiences, and behavioral actions, situated within the scope of their psychological operations.

A participant summarized this concept by stating, (*Response 1*) “*It has helped me stay in the now. I am more conscious of where I am in my thoughts—if I am either in the past or the future, I am aware of bringing it back to what I am experiencing in this moment*.” This observation highlights a heightened mindfulness and deliberate focus on directing their thoughts to the present, exemplifying improved self-regulation.

Another participant reflected on their personal transformation, noting, (*Response 2*) “*For the past few weeks I feel I have changed as a person. I don't get as angry as I used to before. I think I've become more patient*.” This individual's ability to control their emotional states, as evidenced by their decreased anger and increased patience, further underlines the influence of the PMR program on participants' self-control capabilities.

The theme of self-control was also reflected in the participants' behavioral responses, as demonstrated by a participant's comment, (*Response 3*) “*The exercise has helped me sleep better. I used to have too many thoughts that would keep me awake*.” This feedback underscores the program's role in bolstering the participants' capacity to manage intrusive thoughts, contributing to better sleep habits and showcasing enhanced mechanisms of self-regulation.

#### 3.2.2 Self-realization

The theme of “Self-realization” emerged strongly, reflecting participants' insights and understanding of the impacts brought about by the PMR program. This underscores the complex interplay of thoughts and feelings shaping their recognition and evaluation of the program's effects on their psychological health. The narratives afford a profound introspective medium through which we can explore the students' psychosomatic evolution engendered by the curriculum. The incorporation and enactment of educational frameworks within the PMR program are distinctly reflected in the students' phenomenological accounts. These accounts illustrate the significant impact of experiential learning on the process of medical education.

A participant succinctly articulated this cognitive shift, stating, (*Response 1*) “*I didn't realize how tense my muscles are till I did this exercise*.” This remark illuminates the participant's emergent self-awareness of their somatic condition, exemplifying the role of the PMR program in cultivating an integrated understanding of the mind-body connection. Another participant's struggle with focus—(*Response 2*) “*I was comfortable initially but somewhere in the middle, I couldn't focus, and it made me want to finish the exercise*”; this observation highlights the introspective journey toward self-awareness, delineating the cognitive obstacles encountered in sustaining concentration, and acknowledging the necessity for cognitive regulation when confronted with discomfort.

The powerful assertion, (*Response 3*) “*The difference between tension and relaxation made me understand how much stress I have in my body*,” exemplifies an improved somatic realization. The commentary reveals how the PMR program aids in deepening the comprehension of the physical expressions of stress, emphasizing the profound effect of self-awareness within the psychosocial framework of medical training.

#### 3.2.3 Liberation

The theme of 'Liberation' stands out, capturing the students' self-reflective insights that express a deep release from mental constraints after participating in the PMR program. This theme speaks to the psychological release and the heightened sense of freedom they discover on this journey, underscoring the impactful nature of hands-on learning in the intricate world of medical studies.

This concept showcases the freeing effect of the PMR exercise, releasing the participants from fixed ways of thinking and mental inflexibility, leading to a new willingness to embrace experiences. It highlights how the PMR practice is instrumental in promoting mental agility and encouraging a capacity to adapt, which is crucial in the challenging and rigorous educational environment of medical training.

The 'Liberation' theme is vividly embodied in a participant's reflection, (*Response 1)* “*I enjoyed it completely because for once I felt I had nothing to hold onto*.” This quote succinctly captures the essence of liberation—a profound realization of cognitive liberty that marks a significant shift from usual mental limitations. This liberation highlights the pivotal role of the PMR exercise in enhancing cognitive agility and fostering a sense of existential freedom, critical in the intricate psychosocial and educational landscape of medical training.

#### 3.2.4 Awareness

In the context of medical education, the theme of 'Awareness' emerges distinctly through the narratives of students engaging with the PMR. This theme emphasizes the students' increased awareness of their mind-body interplay, a direct effect of the PMR intervention. It underlines the significance of such awareness amidst the intricate web of cognitive, emotional, and physical experiences that medical students navigate. This enhanced awareness is critical for medical students, illustrating how the PMR program not only deepens their understanding of their own psychophysiological states but also serves to cultivate essential metacognitive abilities and a mindful presence. The role of PMR in fostering such skills is crucial in guiding students through a journey of self-exploration and optimal health, which is indispensable within the complex psychosocial and educational framework they encounter.

A participant's statement, (*Response 1*) “*I do the body scan every day and if there's any pain anywhere in my body, I tense and relax just that part of my body and it really helps me relax*,” exemplifies this heightened awareness. This narrative showcases the participants' newfound ability to identify and regulate their bodily sensations. This skill is indicative of their developed psychosomatic mindfulness and self-regulation abilities. Another participant's reflection, (*Response 2*) “*Every time, I am angry, I take deep breaths and try and ground myself. I am aware of the pain in my body and use the muscle relaxation exercise to calm myself down*,” underlines this theme of 'Awareness'. This account highlights how participants use cognitive and physiological self-regulation techniques to control their emotions. Cognitive self-regulation involves the conscious manipulation of thoughts to influence feelings and behaviors. Physiological self-regulation refers to managing physical responses, such as breathing or heart rate, to maintain emotional balance. This process demonstrates the participants' metacognitive awareness—their ability to think about and understand their own thought processes. It also shows their skill in applying learned techniques for emotional stability. The narrative underlines the transformative power of the PMR exercise in developing self-awareness and cognitive resilience. In the complex psychological and educational environment of medical education, these skills are crucial. Cognitive resilience refers to the ability to mentally recover and adapt in the face of stress or adversity. The PMR exercise, by fostering this resilience, plays a significant role in enhancing the psychological wellbeing and professional competence of medical students and practitioners.

#### 3.2.5 Interpersonal relationships

The 'interpersonal relationships' theme emerges as a central concept, illustrating profound shifts in how participants perceive and engage in personal interactions following the PMR exercise. This theme encapsulates changes in relational dynamics and perspective-taking, emphasizing enhanced understanding and adaptation in social exchanges. Key psychological concepts defined within this context include:

1. **Interpersonal modulation**: this term refers to the ability to adjust and adapt behaviors and responses during social interactions. The PMR intervention appears to have enhanced this skill, enabling participants to navigate personal interactions more effectively.
2. **Emotional intelligence**: this is the capacity to understand and manage one's own emotions and to empathize with the emotions of others. The psychosomatic attunement developed through PMR has bolstered this aspect, allowing for better emotional awareness and control.
3. **Relational cognition**: this involves cognitive processes that help in understanding and relating to others. The PMR exercise enhanced participants' ability to comprehend and respond to the emotional states and needs of others, fostering deeper empathy.
4. **Affective resonance**: this is the ability to emotionally connect and attune with others. Through PMR, participants developed a more nuanced understanding of the emotional layers within interactions, improving their emotional resonance in social contexts.
5. **Perspective-taking**: this skill involves seeing situations from others' viewpoints. The PMR intervention has enriched this ability, making participants more adept at understanding diverse perspectives, an essential trait in medical academia and practice.

This theme underscores the PMR intervention's instrumental role in cultivating these vital skills, crucial for navigating the intricate social and emotional terrain of medical education and clinical practice. A participant's reflection, (*Response 1*) “*My relationship with my mom is so much better. When she tells me something I try and not hold onto what is saying if it's not to my liking*,” exemplified this theme of 'Interpersonal Relationships'. This account highlights a deliberate change in how they interact with others, particularly in managing their emotional reactions and understanding different viewpoints in personal communication. This shift indicates improved interpersonal modulation, where the participant shows greater control over their emotional responses during interactions. It also reflects enhanced perspective-taking ability, demonstrating their skill in considering their mother's perspective, even when it differs from their own. These changes are indicative of the broader development of emotional intelligence and affective resonance, key outcomes fostered by the PMR intervention.

Another participant shared, (*Response 2*) “*I don't like my dad too much even now. But I am able to tolerate him without anger filling up in me every time he speaks to me*,” encapsulates the transformative influence of the PMR exercise on the participant's interpersonal relations. This narrative signifies a developed ability to regulate emotional reactions in social interactions, indicating a growth in emotional resilience. The participant's improved tolerance toward their father, despite enduring negative feelings, demonstrates a significant advancement in managing their emotional responses. This change is a clear manifestation of the participant's enhanced emotional intelligence and interpersonal modulation, key outcomes fostered by the PMR intervention. It reflects their ability to navigate challenging emotional scenarios with greater composure and understanding, a vital skill in personal and professional contexts.

These narratives underscore the remarkable potential of the PMR exercise in elevating emotional intelligence, nurturing empathic understanding, and refining interpersonal relationships. Such advancements are instrumental in the comprehensive personal and professional growth of individuals within the demanding environment of medical education. The enhancement in emotional intelligence is evident in the participants' ability to understand and manage their emotions more effectively. The development of empathic understanding is seen in their increased capacity to appreciate and resonate with others' feelings. Moreover, the improvement in interpersonal relationships is reflected in their evolved communication and interaction skills. Collectively, these outcomes highlight how the PMR exercise significantly contributes to equipping medical professionals with the emotional and social competencies necessary for their challenging field.

#### 3.2.6 Theoretical integration and implications

Following the identification of key themes through Braun and Clarke's thematic analysis, it is imperative to integrate these findings within established psychological and educational theoretical frameworks. This integration not only validates our findings but also enhances their applicability in the broader context of medical education and psychological resilience. The themes identified—Self-Control, Self-Realization, Liberation, Awareness, and Interpersonal Relationships—resonate profoundly with several core psychological theories.

##### 3.2.6.1 Self-control and Bandura's self-efficacy theory

The theme of Self-Control aligns closely with Albert Bandura's theory of self-efficacy (Bandura, 2001). According to Bandura, self-efficacy is the belief in one's capabilities to organize and execute the courses of action required to manage prospective situations. The participants' demonstrated proficiency in self-regulation, as evidenced by their enhanced mindfulness and emotional control, echoes Bandura's concept of personal agency. The PMR program, by fostering a heightened sense of control over cognitive processes and affective experiences, appears to bolster the students' belief in their ability to effectively navigate stressful situations, a cornerstone of psychological resilience.

##### 3.2.6.2 Self-realization and Maslow's hierarchy of needs

The theme of Self-Realization can be intricately linked to Abraham Maslow's hierarchy of needs (Hale et al., 2019), specifically the apex of self-actualization. Maslow posited that self-actualization is the realization of one's potential and the most profound level of psychological development. The participants' increased awareness of their somatic conditions and psychological states, as indicated in their reflections, mirrors this journey toward self-actualization. The PMR program seems to facilitate this introspective journey, aiding students in uncovering and embracing their full potential.

##### 3.2.6.3 Liberation and concepts of psychological freedom

Liberation, as a theme, reflects concepts central to positive psychology (Montiel et al., 2021), particularly those concerning psychological freedom and autonomy. This theme underscores the participants' experiences of cognitive liberty and adaptability, key elements in fostering a resilient mindset. The reported feelings of mental emancipation align with the psychological principle that freedom from mental constraints is essential for wellbeing and effective learning, especially in high-stress environments like medical training.

##### 3.2.6.4 Awareness and metacognitive processes

The theme of Awareness ties in with the concept of metacognition found in educational psychology (Flavell, 1979). Metacognition refers to the awareness and understanding of one's own thought processes. The participants' ability to conduct body scans and regulate their emotional states through PMR practices demonstrates an enhanced level of metacognitive awareness. This heightened awareness is crucial in medical education, where understanding and managing one's cognitive and emotional processes can significantly impact learning efficiency and professional competence.

##### 3.2.6.5 Interpersonal relationships and emotional intelligence

Finally, the theme of Interpersonal Relationships can be linked to the theories of emotional intelligence and social learning (Tang and He, 2023). The participants' reflections on improved relational dynamics and empathic engagement indicate an enhancement in emotional intelligence—the ability to perceive, assess, and manage one's own emotions and understand the emotions of others. This development is vital in the context of medical education, where effective communication and empathetic patient care are paramount.

In conclusion, the integration of our thematic findings with these theoretical frameworks provides a deeper understanding of how PMR contributes to enhancing resilience in medical students. This theoretical grounding not only strengthens the validity of our findings but also underscores the relevance and applicability of PMR in cultivating psychological resilience and emotional intelligence in the demanding field of medical education.

#### 3.2.7 Practical implications of identified themes on student resilience during the COVID-19 pandemic

The identified themes—self-control, self-realization, liberation, awareness, and interpersonal relationships—have profound implications for medical students' resilience, especially under the extraordinary circumstances of the COVID-19 pandemic.

##### 3.2.7.1 Self-control in pandemic stress management

Enhanced self-regulation skills were vital for students during high-stress periods of the pandemic (Lu et al., 2022; Xu et al., 2022). The ability to manage intense emotions and stress through PMR techniques was a critical factor in maintaining mental wellbeing.

##### 3.2.7.2 Awareness and early burnout detection

Increased awareness, both mental and physical, gained importance during COVID-19 (Shi et al., 2020). This heightened sensitivity to stress and exhaustion signs allowed students to apply PMR methods proactively, preventing burnout in a high-pressure environment.

##### 3.2.7.3 Interpersonal relationships and empathetic patient care

The development of empathetic communication skills, as fostered by PMR, became crucial in-patient care during the pandemic (Stevens et al., 2020; Faghihi et al., 2024). Improved emotional understanding and communication aided in better connecting with and supporting patients experiencing fear and isolation.

##### 3.2.7.4 Liberation and cognitive flexibility

The theme of liberation, indicating mental adaptability, was reflected in students' ability to navigate the constantly evolving pandemic situation (Besser et al., 2022; Lassri, 2023). The cognitive flexibility fostered by PMR was key in adapting to changing protocols and uncertainties.

In summary, these themes underscore the significant role of the PMR program in bolstering resilience, particularly in addressing the unique challenges posed by the COVID-19 pandemic, thereby affirming its relevance and effectiveness in medical education.

### 3.3 Adherence to the SRQR

Our study closely adhered to the SRQR as proposed by O'Brien et al. (Table 2). The title and abstract were concise and descriptive, presenting a comprehensive overview of our study. The introduction appropriately addressed the problem and outlined the purpose of our study. The methods section demonstrated our adoption of a grounded theory approach and considered researcher characteristics and reflexivity. The sampling strategy was clearly described, and ethical issues pertaining to human subjects were effectively managed. Our data collection methods and analysis demonstrated depth and rigor, and we ensured the trustworthiness of the study through established strategies. Our findings were synthesized and interpreted thoughtfully, maintaining robust links to empirical data. The discussion was comprehensive, integrating our findings with prior work and considering the limitations of our study. Conflicts of interest were appropriately managed, and funding sources were disclosed. Our compliance with the SRQR (Table 2) lends credence to the quality of our work and provides a solid foundation for its acceptance in the scientific community. In adhering to the SRQR guidelines, our study not only aligns with recognized research standards but also enhances its credibility and reliability. This adherence ensures that our research methods and findings are both transparent and rigorous, making a significant contribution to the field and providing a trustworthy reference for future studies.

**Table 2:** Standards for reporting qualitative research by O'Brien et al.

**Standards for Reporting Qualitative Research by O'Brien et al**.	**Description of the standard as defined by O'Brien et al**.	**Alignment of the present study with the standards**	**Comments**
**Title and Abstract**			
S1. Title	• Concise description of the nature and topic of the study • Identifying the study as qualitative or indicating the approach (e.g., ethnography, grounded theory) or data collection methods (e.g., interview, focus group) is recommended	**√**	• Our title attests to the standard as we have identified the instructional design model, as well as the topic of the study. • Further, since we had fewer number of samples, as well as all our participants were all female, we have categorized our study as a proof-of-concept study.
S2. Abstract	• Summary of key elements of the study using the abstract format of the intended publication; typically includes background, purpose, methods, results, and conclusions	**√**	• We have a structured abstract describing the background, methods, purpose, results, and conclusions, where we have summarized the key elements of the study.
**Introduction**			
S3. Problem formulation	• Description and significance of the problem/phenomenon studied; review of relevant theory and empirical work; problem statement	**√**	• In our study, we have identified the detrimental effects of stress on medical education (i.e., problem). • We have also identified that stress can exert more detrimental effects in medical students in the Middle East due to the stigma associated with mental health (i.e., elaboration of the regional problem). • We have identified the method(s) which have been implemented in medical school for attenuating stress and improving mental wellbeing through a literature review (i.e., review of relevant studies to improve mental wellbeing). • Identification of MBSR as the most implemented technique to counter stress in medical schools, concurrently identifying the fact that implementation of MBSR requires significant training and resources (i.e., problem statement highlighting the issue addressed in this study).
S4. Purpose or research question	• Purpose of the study and specific objectives or questions	**√**	• We have delineated the purpose which is to identify and implement a technique that will replicate the benefits of MBSR without putting a constraint on resources and time.
**Methods**			
S5. Qualitative approach and research paradigm	• Qualitative approach (e.g., ethnography, grounded theory, case study, phenomenology, narrative research) and guiding theory if appropriate; identifying the research paradigm (e.g., postpositivist, constructivist/interpretivist) is also recommended; rationale	**√**	• Our research is guided by the precepts of grounded theory.
S6. Researcher characteristics and reflexivity	• Researchers' characteristics that may influence the research, including personal attributes, qualifications/experience, relationship with participants, assumptions, and/or presuppositions; potential or actual interaction between researchers' characteristics and the research questions, approach, methods, results, and/or transferability	**√**	• We have identified the job description of all the researchers involved in this study. We have ensured that is no existence of power differential between the researchers and the participants. • We ensured that qualified/trained personnel were involved in the design and implementation of the PMR technique.
S7. Context	• Setting/site and salient contextual factors; rationale	**√**	• Our study has identified the study landscape alluding to the curriculum architecture of our undergraduate medical program. • For the PMR sessions, we have described the setting in detail such that the technique and its benefits can be replicated in any geographical location.
S8. Sampling strategy	• How and why research participants, documents, or events were selected; criteria for deciding when no further sampling was necessary (e.g., sampling saturation); rationale	^*^	• As this was a proof-of-concept study, we did not perform any statistical power calculation nor avail a guided sampling strategy. • Participant recruitment was on a first come first serve basis and was capped at 20, adhering to mandated COVID-19 protocols.
S9. Ethical issues pertaining to human subjects	• Documentation of approval by an appropriate ethics review board and participant consent, or explanation for lack thereof; other confidentiality and data security issues	**√**	• Verbal consent was obtained from all the 20 participants. • No ethical clearance was required for this study as this was part of quality improvement of the disseminated curriculum.
S10. Data collection methods	• Types of data collected; details of data collection procedures including (as appropriate) start and stop dates of data collection and analysis, iterative process, triangulation of sources/methods, and modification of procedures in response to evolving study findings; rationale	**√**	• Needs assessment, using guided literature survey, was pursued to identify the method that has been employed in medical education to augment students' mental wellbeing (Supplementary Table 1) • Participant recruitment was on a first-come-first-serve basis. All recruited participants, undertaking PMR, were included in two 60-min focus group sessions (i.e., 10 participants in each session). • Data obtained from the participants was analyzed following thematic analysis strategy in line with Braun and Clarke's framework. • The study architecture was assessed in line with *O'Brien's SRQR checklist*.
S11. Data collection instruments and technologies	• Description of instruments (e.g., interview guides, questionnaires) and devices (e.g., audio recorders) used for data collection; if/how the instrument(s) changed over the course of the study	**√**	• Interview structure was blueprinted according to the McKinsey 7S framework.
S12. Units of study	• Number and relevant characteristics of participants, documents, or events included in the study; level of participation (could be reported in results)	**√**	• Twenty undergraduate medical students were recruited was on a first-come-first-serve basis. As this was a proof-of-concept study, we did not perform any statistical power calculation nor avail a guided sampling strategy.
S13. Data processing	• Methods for processing data prior to and during analysis, including transcription, data entry, data management and security, verification of data integrity, data coding, and anonymization/deidentification of excerpts	**√**	• Manual transcription of focus group interviews was independently performed by two investigators (BN, NN) to minimize subjectivity
S14. Data analysis	• Process by which inferences, themes, etc., were identified and developed, including the researchers involved in data analysis; usually references a specific paradigm or approach; rationale	**√**	• Focus group discussions were transcribed verbatim and transcripts were subjected to interpretive qualitative data analysis, following a Grounded theory approach. • Data analysis was predominantly inductive and consisted of thematic analysis based on the six steps identified by Braun and Clarke's framework (i.e., familiarization; coding; generating themes; reviewing themes; defining and naming themes; and writing up). • A list of codes was developed and reviewed by the research team until consensus was reached. • As coding progressed, new codes were generated; and existing codes refined. • Once the coding of data was completed, connection between the codes led to the identification of thematic categories and ultimately to the development of an overriding explanation.
S15. Techniques to enhance trustworthiness	• Techniques to enhance trustworthiness and credibility of data analysis (e.g., member checking, audit trail, triangulation); rationale	**√**	• The following strategies were employed to increase trustworthiness of data in line with the guidelines of Lincoln and Guba (1985). Naturalistic inquiry. sage: (1) The facilitator who conducted the focus group sessions performed the data analysis; (2) The coding and themes were checked and refined by multiple members in the research team; (3) Data collection and analysis were performed simultaneously; (4) Each aspect of data collection and analysis were guided by well-established frameworks implemented in health professions and medical education.
**Results/findings**			
S16. Synthesis and interpretation	• Main findings (e.g., interpretations, inferences, and themes); might include development of a theory or model, or integration with prior research or theory	**√**	• Interpretation and analysis were pursued in line with Braun and Clarke's framework. • Based on the obtained data analysis, it was agreed upon by the research team that a change management model is essential for the implementation of PMR in medical education. In line, Mento's change management model was adopted based on prior experience of the research team in this domain.
S17. Links to empirical data	• Evidence (e.g., quotes, field notes, text excerpts, photographs) to substantiate analytic findings	**√**	Relevant quotes categorized under different themes have been presented in the current manuscript
**Discussion**			
S18. Integration with prior work, implications, transferability, and contribution(s) to the field	• Short summary of main findings; explanation of how findings and conclusions connect to, support, elaborate on, or challenge conclusions of earlier scholarship; discussion of scope of application/generalizability; identification of unique contribution(s) to scholarship in a discipline or field	**√**	• Summary of key findings has been presented highlighting the importance of the present proof-of-concept study. • One of the key challenges, as identified in the introduction, is adherence to the technique, requiring its integration within the medical curriculum. • To facilitate its integration into undergraduate medical curriculum, we have employed Gagne's instructional design model and Mento's change management model. • To our knowledge, this is the first study integrating PMR in undergraduate medical education using the above-validated frameworks.
S19. Limitations	• Trustworthiness and limitations of findings	**√**	The aspect of trustworthiness has been addressed in S15. Limitations with regard to the number and gender of participants has been discussed extensively under Limitations of the Study in the present manuscript.
**Other**			
S20. Conflicts of interest	Potential sources of influence or perceived influence on study conduct and conclusions; how these were managed	**√**	None of the researchers participating in this study declared any conflict of interest. This has been indicated in line with journal requirements.
S21. Funding	Sources of funding and other support; role of funders in data collection, interpretation, and reporting	**√**	Funding was obtained from office of Provost Mohammed Bin Rashid University of Medicine and Health Sciences (MBRU, Dubai Health) to defray the necessary expenses associated with the conduct and dissemination of the present study. This has been indicated in manuscript under ‘Funding'.

## 4 Discussion

This section aims to explain and interpret the thematic findings from our qualitative investigation of the PMR intervention within the context of CBME. We explore themes such as self-control, self-realization, liberation, awareness, and interpersonal relationships to understand the PMR intervention's multifaceted impact. Our goal is to assess its effectiveness using various theoretical frameworks, pedagogical paradigms, and sociocultural theories.

From a pedagogical perspective, the PMR intervention's interaction with CBME principles highlights the integration of cognitive, affective, and psychomotor learning domains. We also use a sociocultural theoretical lens to examine the relationship between individuals' psychological states and the broader social structure in medical education.

Incorporating transformative learning theory (Youssef, 2022), this discussion aims to illuminate how PMR fosters critical self-reflection, cognitive flexibility, and affective regulation among learners. This process aids in developing skilled, empathetic, and resilient healthcare professionals. Additionally, we apply Bourdieu's “Theory of Practice” to explore the intervention's influence on habitus, capital, and field within the sociocultural context of medical education.

Furthermore, using Mento's Change Management Model, we propose a structured approach for integrating PMR into the CBME curriculum. This strategy encompasses changes at both the instructional level and the broader educational philosophy. Our aim is to cultivate a resilient, compassionate, and skilled medical community.

### 4.1 Theme: self-control

#### 4.1.1 Autonomy and volition: the interplay of self-control and medical education

Mindfulness-based training interventions like PMR emphasize the importance of being fully present in the moment, a deliberate and non-judgmental act (Ludwig and Kabat-Zinn, 2008). These methods provide a practical realignment, especially when internal disturbances shift focus, aiming to sidestep immediate demands (Agee et al., 2009; Pintimalli et al., 2023). Similarly, in our study, students reflected this cognitive shift. As illustrated in ***Response 1***, they recognized their thoughts drifting between past and future, yet they learned to control this mental meandering and develop a consistent awareness of the present, thanks to PMR exercises.

PMR encourages a detached connection with challenging experiences, increasing mindfulness by highlighting the mind's tendency to dwell on factors beyond control (Matsumoto and Smith, 2001; Toussaint et al., 2021). It enhances focused attention (Wimmer et al., 2020; Phan et al., 2022), thereby strengthening the ability to employ attention-regulation strategies effectively. In medical education, for instance, this skill is critical in high-stress environments like emergency rooms, where maintaining focus amidst chaos can be pivotal.

***Response 1*** also hints at a propensity for rumination, a counterproductive process of continuously pondering over past difficulties, their causes, and effects (Li et al., 2022). This often involves a negative self-assessment due to persistent focus on past distresses. PMR, by centering attention on the present, helps break free from these ruminative loops. It cultivates an openness to the current experience, reducing self-critical judgments (Shapiro et al., 2006; Brami et al., 2023). In healthcare settings, this ability to shift from ruminative thinking to present-moment awareness can be invaluable, aiding healthcare professionals in managing stress and maintaining clarity of thought during critical patient care.

In conclusion, Incorporating PMR into medical training transcends mere cognitive benefits, significantly bolstering *autonomy* and *volition* among medical practitioners. By fostering a clear, focused, and present-centered mindset, PMR empowers medical students and professionals to make independent, informed decisions—akin to a doctor autonomously diagnosing and treating patients while considering their unique contexts. This development of autonomy is crucial for effective and personalized patient care. Simultaneously, PMR supports volition, the drive to act intentionally according to one's professional values. For example, a medical student displaying volition might actively seek challenging cases or additional training to enhance their skills. Thus, through PMR, individuals in the medical field are equipped not only with refined cognitive abilities but also with the self-direction and motivation (*volition*) essential for excelling in the complex and dynamic landscape of healthcare. This comprehensive empowerment ensures that healthcare professionals are skilled, autonomous, and motivated, striving for excellence in patient care.

#### 4.1.2 Regulation of emotional responses: role of PMR in behavioral modifications

***Response 2*** highlights PMR's transformative effect on individuals' emotional states. Empirical studies, like those by Ye et al. (2021), confirm PMR's efficacy in reducing anger and aggression. Often, heightened anxiety is linked to '*anger-in, anger-out expression*,' a pattern that can be diminished through relaxation techniques like PMR (Seyedi Chegeni et al., 2018; Icel and Basogul, 2021). PMR not only addresses the outward manifestations of anger but also targets the underlying mental states, promoting balanced behaviors (Icel and Basogul, 2021). This effect is particularly crucial in medical settings, where students frequently face high-pressure situations.

Challenging clinical scenarios, such as complex diagnostic puzzles, high-stakes surgeries, difficult patient interactions, or emotionally charged end-of-life conversations, demand a steady emotional state. Here, equanimity acts as a shield against emotional distress and cognitive overload, protecting learners' mental wellbeing and enabling sound, evidence-based decisions. It also supports maintaining a therapeutic alliance with patients and caregivers and ensures productive interactions with the healthcare team.

Emotional intelligence in medical education, as outlined by Cherry et al. (2014), is integral to navigating the intricate landscape of medical practice. Comprising self-awareness, self-regulation, motivation, empathy, and social skills, it's pivotal for resilience, burnout mitigation, empathy enhancement, and delivering patient-centered care (Johnson, 2015). Cultivating emotional intelligence and equanimity in medical students is not just an additional aim but a fundamental aspect of CBME.

Furthermore, unregulated anger can lead to adverse psychosocial outcomes like anxiety, depression, or violence, potentially jeopardizing patient safety (Walsh et al., 2018; De Bles et al., 2019). Hence, integrating PMR into medical education fosters not only the emotional regulation crucial for personal wellbeing but also the autonomy and volition necessary for professional excellence. By enabling future healthcare professionals to master their emotional responses, PMR empowers them to navigate the complex emotional landscapes of healthcare with greater confidence and competence. This mastery is essential for delivering high-quality, empathetic patient care, highlighting PMR's indispensable role in shaping resilient, emotionally intelligent medical practitioners.

#### 4.1.3 The sleep-thought Nexus: PMR's influence on sleep quality

***Response 3*** highlights the complex link between intrusive thoughts and impaired sleep quality. PMR, which is based on achieving muscle relaxation after tension, fosters a '*cultivation of muscle sense'* (Toussaint et al., 2021). This deep relaxation is linked to reduced brain electrical activity, helping to alleviate symptoms of insomnia (Wolkove et al., 2007; Kobayashi and Koitabashi, 2016). Research supports PMR's effectiveness in improving sleep quality (Harorani et al., 2020; Liu et al., 2020), likely due to its role in increasing melatonin and serotonin production (Stacchiotti et al., 2020). These hormones are critical for natural healing processes during deep sleep, which can reduce mental health issues related to poor sleep (Kanady et al., 2018; Lin et al., 2022).

Long-term effects of disturbed sleep, such as chronic fatigue, impaired cognitive function, and increased susceptibility to mental health disorders like anxiety and depression, are well-documented (Institute of Medicine Committee on Sleep Research, 2006). In medical education and healthcare settings, where professionals often face sleep disruptions due to irregular shifts and high-stress environments, PMR can be a valuable tool. By enhancing sleep quality, PMR not only addresses immediate restfulness but also contributes to long-term wellbeing, helping healthcare professionals maintain focus, decision-making capabilities, and emotional stability.

While ***Response 3*** underscores the direct benefit of improved sleep, it also connects to the broader theme of stress reduction and enhanced attention, as outlined in *Response 1*. Good sleep is crucial for reducing stress and increasing focus, aligning with Step 9 of Gagne's instructional design model (Table 1). It also resonates with the key domains of the learning outcomes frameworks in medical education.

Incorporating PMR into medical training goes beyond immediate relaxation benefits. It aids in developing long-term strategies for managing sleep disturbances, thereby enhancing overall wellbeing and professional efficacy. For medical students and professionals, mastering this technique can lead to improved cognitive function, emotional regulation, and better patient care. Ultimately, PMR not only fosters immediate relaxation and sleep improvement but also contributes to the long-term autonomy and volition of healthcare professionals, essential for navigating the demanding landscape of medical practice.

#### 4.1.4 Fostering self-control in medical education: integrating with competency-based frameworks

*Self-control*, defined as the dynamic interaction of cognitive, emotional, and behavioral regulation underpinned by metacognitive processes (Panadero, 2017), is essential in medical education. It enables adaptive control across various medical competencies, aligning with outcomes defined in Competency-Based Medical Education (CBME) frameworks (Figure 3).

The Accreditation Council for Graduate Medical Education (ACGME) in the United States outlines six core competencies: patient care, medical knowledge, practice-based learning and improvement, interpersonal and communication skills, professionalism, and systems-based practice (Nasca et al., 2012). Self-regulation is foundational to these competencies. For example, practice-based learning and improvement involve self-evaluation, goal setting, and engagement in enhancement strategies, aligning with Zimmerman's self-regulation model phases (Zimmerman, 1986): forethought, performance, and self-reflection (Panadero, 2017).

Similarly, the CanMEDS Physician Competency Framework by the Royal College of Physicians and Surgeons of Canada details seven roles for physicians: medical expert, communicator, collaborator, leader, health advocate, scholar, and professional (Thoma et al., 2023). Each role requires sophisticated self-control, enabling medical students to modulate their cognitive, emotional, and behavioral responses in various contexts. For instance, as a 'medical expert', a physician needs to manage cognitive complexities and apply critical thinking in clinical decisions. The 'communicator' role demands emotional intelligence for empathetic patient interactions and effective communication. The 'collaborator' role requires behavioral flexibility to work in teams and resolve conflicts. The other roles further elaborate on aspects like strategic thinking, ethical practice, lifelong learning, and health advocacy. CanMEDS roles thus necessitate refined self-control, essential for navigating the complexities of medical practice (Thoma et al., 2023).

The Scottish Doctor Framework and the GMC UK Competency Framework also emphasize self-control in clinical skills, knowledge, attitudes, professionalism, and relationship management (Simpson et al., 2002; Al-Shakarchi et al., 2019). The Brown Abilities Competency Framework highlights managing one's learning, patient care, and teamwork in healthcare settings (University, 2021).

Integrating PMR exercises into medical education enhances self-control, facilitating the achievement of these competencies. Students in this study reported improved thought, emotion, and behavior control through PMR, enhancing their academic and professional performance. In conclusion, self-control aligns with CBME competencies and is critical for developing adaptive, competent, and resilient physicians. By fostering self-control, medical education can meet contemporary healthcare demands, enhancing students' autonomy and volition in their professional journey.

#### 4.1.5 Pedagogical shift during COVID-19: the pivotal role of self-control and the potential benefits of PMR in the post-pandemic landscape

The COVID-19 pandemic catalyzed a significant shift in educational methodologies, necessitating a rapid transition from traditional, in-person teaching to digital and blended learning approaches. This shift underscored the importance of self-control in the educational context. According to Bandura's social cognitive theory, self-control is a pivotal aspect of self-regulated learning, enabling students to effectively manage their cognitive, emotional, and behavioral processes in pursuit of academic goals (Bandura, 2001).

In the realm of digital learning, characterized by numerous distractions, ambiguous tasks, and often, social isolation, self-control emerges as a crucial skill. It empowers students to maintain focus, navigate uncertain situations, manage stress, and adhere to a disciplined study regimen (Alt and Naamati-Schneider, 2021). Additionally, aligning with Vygotsky's sociocultural theory, self-control enhances learners' ability to engage in co-regulated and socially mediated regulation in online collaborative settings. This enhancement is crucial for fostering effective communication, resolving conflicts, and engaging in collective problem-solving (Van Der Veer, 2021).

The development of self-control, as facilitated through PMR, is not only vital in addressing immediate challenges brought about by the pandemic but also in preparing students for the evolving landscape of education post-pandemic. As educational frameworks increasingly integrate technology and hybrid models, students will continuously face new challenges and opportunities. The self-control skills honed through PMR are instrumental in fostering adaptability, resilience, and ongoing personal and academic development in these dynamic educational environments.

Therefore, PMR's role extends beyond mitigating the immediate psychological impacts of the pandemic; it equips learners with the necessary tools to excel in the increasingly complex, diverse, and uncertain future of education. This capacity for self-control, nurtured through PMR, contributes significantly to the broader field of medicine and healthcare, particularly in preparing future healthcare professionals who must navigate an ever-changing landscape of medical technology, patient interaction, and healthcare management (Callus et al., 2020).

In summary, the integration of self-control strategies like PMR in educational curricula not only responds to the immediate needs of the pandemic but also strategically prepares learners for the future challenges and advancements in both education and healthcare sectors.

#### 4.1.6 Self-control in medical education: a Bourdieusian perspective and its broader implications

Bourdieu's Theory of Practice offers a compelling framework for understanding the role of self-control in medical education and the professional development of medical students. Through Bourdieu's lenses of habitus, capital, and field, we see a dynamic interplay between internal dispositions, available resources, and external contexts (Alecu et al., 2022). Within this framework, self-control—encompassing cognitive, emotional, and behavioral aspects—emerges as a pivotal component of the habitus. This is the complex web of ingrained dispositions and capabilities shaped by an individual's experiences and social context.

In the context of medical training, self-control, enhanced through practices like PMR, is critical. It cultivates an adaptable *habitus*, equipping future medical professionals to adeptly navigate the diverse and demanding aspects of the healthcare field with composure and proficiency. Regular practice of self-control enriches the '*cultural capital'* of medical students, broadening their skills and granting them an advantage in the competitive landscape of the medical profession.

This enhanced resilience is crucial in addressing the inherent stressors of the *medical field*. It improves performance under high-pressure situations and aids in the intricate management of clinical cases and interpersonal relations. Consequently, this process significantly influences their professional trajectory, molding their developing physician identity to align with the highest ethical and professional standards. Such a foundation is imperative for a successful medical career in the post-COVID era and beyond, where adaptability and resilience are increasingly valued.

The implications of this integrated approach to self-control in medical education extend beyond individual practitioners. By fostering these skills, medical education can contribute to a healthcare workforce better equipped to handle the complexities of modern medicine. This includes navigating evolving medical technologies, addressing patient needs in diverse settings, and managing the challenges of healthcare systems. In essence, embedding self-control within the medical curriculum, viewed through a Bourdieusian lens, not only enriches the individual learner but also bolsters the broader field of healthcare by preparing competent, adaptable, and resilient professionals.

In summary, Bourdieu's Theory of Practice provides a valuable theoretical perspective to understand and enhance the role of self-control in medical education. This approach not only benefits individual learners but also significantly contributes to the advancement and efficacy of the healthcare sector as a whole.

### 4.2 Theme: self-realization

#### 4.2.1 Enhancing stress awareness through psycho-physiological understanding in medical education

The intricate relationship between psychological experiences and physiological responses is a fundamental aspect of human behavior, particularly evident in stress-related responses. Research has consistently shown that stress manifests in various physiological symptoms, such as increased heart rate, elevated blood pressure, and cortisol production (Toussaint et al., 2021). This psycho-physiological link reflects our innate reactions to perceived stressors and is well-documented in the literature (Mcewen and Gianaros, 2010; Hao and Farah, 2023).

In the context of medical education, where students often face intense academic pressures, understanding this relationship is crucial. The identification of muscular tension serves as an indicator of stress levels, providing a tangible measure of the body's response to stress. This recognition is not only a practical demonstration of the contrast between tensed and relaxed muscle states but also represents a deeper, self-reflective insight, a core component of the PMR (Progressive Muscle Relaxation) technique. This awareness aligns with Steps 6 and 9 of Gagne's instructional design model, which emphasize the importance of feedback and enhancing the learning process through the application of acquired skills (Table 1).

The significance of this psycho-physiological convergence in medical education extends beyond individual stress management. By cultivating an awareness of stress responses and learning techniques like PMR to manage them, medical students can develop crucial skills for their future professional roles. Stress management is not only vital for personal wellbeing but also has implications for patient care. Physicians who are adept at managing their stress can maintain a higher level of focus, make more accurate diagnoses, and provide better patient care. Moreover, stress awareness and management skills are essential for navigating the often-high-pressure environment of healthcare settings, contributing to a more effective and empathetic healthcare workforce.

In summary, the psycho-physiological understanding of stress, particularly within the demanding environment of medical training, is vital. Integrating stress awareness and management techniques into the medical curriculum not only benefits the individual learner but also enhances the overall quality of healthcare delivery by preparing future physicians who are well-equipped to handle personal and professional stressors.

#### 4.2.2 Integrating fluid awareness and mindfulness in medical education: addressing the challenges and enhancing resilience

The concept of fluid awareness, as emphasized in the work of Kabat-Zinn, plays a pivotal role in the understanding and practice of mindfulness (Ludwig and Kabat-Zinn, 2008; Brami et al., 2023). This notion pertains to the evolving nature of perception and consciousness, particularly in the context of mindfulness exercises like PMR. The PMR exercise facilitates a transition in students' perspectives, allowing them to momentarily become attuned to subtle changes in their physical and mental states. This aspect of mindfulness underscores the experiential learning elements of PMR, reinforcing the idea that students can develop an acute awareness of their bodily and mental processes.

However, achieving this level of fluid awareness requires disengaging from entrenched patterns of thoughts and emotions, a process that often presents initial challenges. Cultivating a stable and mindful state of being is a skill that demands both consistent practice and time (Fortney and Taylor, 2010; Demarzo et al., 2014). The discomfort or difficulty expressed by students, as indicated in ***Response 2***, is indicative of the initial struggles often encountered in adhering to mindful practices. Such experiences are a natural part of the learning process, especially when new techniques push individuals out of their comfort zones (Fortney and Taylor, 2010).

The relevance of fluid awareness and the challenges associated with its development extend beyond the personal benefits of mindfulness. In the field of medicine and healthcare, the ability to maintain a mindful state has significant implications. For medical professionals, developing mindfulness can enhance their ability to remain present and focused, leading to improved decision-making and patient interactions (Liu et al., 2022). Furthermore, the skills acquired through mindfulness practices can be instrumental in managing the high levels of stress and emotional demands characteristic of medical careers. This not only benefits the wellbeing of healthcare providers but also positively impacts patient care and the healthcare system as a whole.

In summary, incorporating the concept of fluid awareness into medical education, particularly through mindfulness practices like PMR, not only enriches the personal development of medical students but also prepares them for the multifaceted challenges of their future professional roles. This integration of mindfulness into the curriculum is crucial for cultivating a healthcare workforce capable of managing personal stress, maintaining focus under pressure, and delivering compassionate, patient-centered care.

#### 4.2.3 Exploring the interplay between stress, physical relaxation, and mental wellbeing in medical education

***Response 3*** illuminates a significant realization among medical students: the intricate connection between stress, its physical manifestations, and the role of Progressive Muscle Relaxation (PMR) in mitigating these effects. This insight aligns with Steps 6 and 9 of Gagne's instructional design model (Table 1), underscoring the comprehensive nature of wellbeing. It suggests that physical relaxation, facilitated by PMR, is not just an end but a foundational step toward achieving mental stability (Fortney and Taylor, 2010).

For medical students, who often face a barrage of clinical and academic challenges, understanding and leveraging this relationship is vital. It equips them with a practical and accessible tool for managing stress. PMR, being a low-cost and self-administered technique, offers an invaluable resource for students to effectively navigate the pressures inherent in their demanding educational and future professional environments (Gallego-Gomez et al., 2020; Liang et al., 2020).

The significance of this finding extends to the broader field of medicine and healthcare. In a profession where stress is a constant and can adversely affect both practitioners and patient care, the ability to manage stress through physical relaxation techniques is crucial. The practice of PMR and similar techniques can lead to improved concentration, decision-making, and emotional resilience for medical professionals. This, in turn, has the potential to enhance patient interactions and outcomes, as healthcare providers who are mentally stable and less stressed are more likely to be attentive and empathetic in their patient care (Ford et al., 2023).

In summary, embedding the understanding and practice of stress management through physical relaxation techniques like PMR into medical education not only benefits the individual learner but also contributes to the cultivation of a healthcare workforce that is better prepared to handle the rigors of the profession. This proactive approach to stress management is essential in fostering a resilient, effective, and compassionate medical community.

#### 4.2.4 Enhancing medical competencies through self-realization via PMR

The journey of self-realization facilitated by PMR exemplifies several competencies outlined in international medical education frameworks (Figure 3). The ACGME emphasizes the importance of compassionate, appropriate, and effective patient care (Nasca et al., 2012). PMR, by promoting deep self-awareness, inherently enhances empathy, a cornerstone of patient-centered care (Robinson et al., 2023). This understanding of the self is crucial in developing the sensitivity and attentiveness required for effective patient interactions.

Furthermore, self-realization through PMR aligns with the 'Scholar and Educator' competency of the CanMEDS Framework (Thoma et al., 2023). It fosters the ability to critically engage with, assimilate, and apply new knowledge, underpinning the culture of lifelong learning that is essential in medicine (Teunissen and Dornan, 2008). This aspect of PMR encourages continuous personal and professional growth, a key attribute in the ever-evolving field of healthcare.

Drawing from social learning theory (Mukhalalati et al., 2022), PMR can be integrated into the 'Professional' competency of the CanMEDS Framework and the Brown Abilities Competency Framework (Batt et al., 2021). Both frameworks stress adherence to ethical standards and a commitment to self-care and the wellbeing of others. By enhancing self-awareness and mindfulness, PMR contributes to ethical decision-making and effective self-care practices (Sevinc and Lazar, 2019; Feruglio et al., 2022).

The GMC UK (Southgate et al., 2001) and the Scottish Doctor Framework (Ellaway et al., 2007) also align with the benefits of PMR. By fostering a deeper understanding of one's emotional and physical states, PMR equips medical practitioners with enhanced communication skills, essential for building rapport and trust with patients. This aligns with the 'Interpersonal and Communication Skills' competency of the ACGME and the 'Communicator' role in the CanMEDS Framework.

Bourdieu's Theory of Practice (Rhynas, 2005) further highlights the importance of understanding one's habitus—personal history, social, and cultural context—enhanced through the self-awareness that PMR promotes. This deeper self-knowledge informs and improves doctor-patient interactions, fostering an environment of mutual respect and understanding.

Incorporating PMR into medical education and practice thus cultivates a wide range of competencies, benefiting not only the individual practitioner but also enhancing the quality of patient care and contributing positively to the broader medical community. This integration underscores the multifaceted impact of mindfulness practices in medical training, preparing practitioners who are not only technically proficient but also emotionally intelligent and ethically grounded.

#### 4.2.5 Advancing medical education through self-realization in the COVID-19 era and beyond

The COVID-19 pandemic has undeniably catalyzed a rapid transformation in medical education, shifting from traditional in-person methods to remote and digital learning environments. While this change was initially a response to an unprecedented crisis, it has inadvertently created an opportunity to rethink and modernize medical education. Integrating progressive practices like PMR into this new pedagogical landscape aligns with key psychological and educational theories, including Bandura's social learning theory (Horsburgh and Ippolito, 2018), cognitive load theory (Van Merrienboer and Sweller, 2010; Young et al., 2014), and Maslow's hierarchy of needs (Hale et al., 2019).

Utilizing PMR as a pedagogical tool embodies the essence of Bandura's social learning theory (Bandura, 2001). Medical students not only learn the relaxation technique itself but also develop cognitive skills to recognize and manage their bodily and emotional responses to stress. This process fosters self-efficacy and resilience (Liu et al., 2021), qualities that are increasingly important given the heightened demands and uncertainties faced by healthcare professionals during and after the pandemic.

In terms of cognitive load theory, which emphasizes the need to manage the volume of information and tasks that learners are exposed to (Van Merrienboer and Sweller, 2010; Young et al., 2014), PMR plays a vital role. By aiding in the management of mental workload, PMR enhances cognitive processing capabilities and academic performance, making it a valuable addition to medical education.

Moreover, from the perspective of Maslow's hierarchy of needs, the self-awareness and personal growth achieved through PMR practice address higher-level psychological needs such as esteem and self-actualization (Hale et al., 2019). These needs are critical in shaping effective physician-patient interactions and contributing to comprehensive patient care.

Looking ahead to the post-COVID era, the need for self-aware and resilient physicians is more pressing than ever (Sovold et al., 2021). The evolving nature of medical practice, coupled with its inherent challenges, demands a forward-thinking approach in medical education. PMR, with its emphasis on self-realization, offers a relevant and progressive strategy to prepare future physicians for the complexities of their profession. By integrating PMR into the curriculum, medical education can adapt to the dynamic requirements of the global health landscape, equipping future healthcare professionals with the skills to navigate their careers effectively and compassionately.

#### 4.2.6 Enhancing medical education and career development through self-realization: insights from Bourdieu's Theory of Practice

Bourdieu's Theory of Practice provides a robust theoretical framework for understanding the profound impact of self-realization on medical education and career trajectories (Rhynas, 2005). When applied to medical students and healthcare professionals, self-realization, particularly as cultivated through PMR, significantly influences the various forms of capital recognized by Bourdieu, enriching both the educational experience and professional development.

Consider a medical student who engages in PMR. This practice not only initiates cognitive metacognition and emotional regulation but also enriches their cultural capital. It develops their cognitive structures and academic skills, aligning with Vygotsky's concept of the Zone of Proximal Development (Esteban-Guitart, 2018). This enhanced cognitive capacity is crucial in navigating intricate medical concepts, honing problem-solving skills, and advancing clinical reasoning abilities—skills that are fundamental to medical practice. For instance, a student who has developed robust self-awareness through PMR may approach complex patient cases with greater clarity and insight, facilitating better diagnostic reasoning and patient care.

Additionally, the role of self-realization in augmenting social capital is evident in the realm of medical education and practice. By fostering emotional intelligence and empathy, PMR aids medical students and professionals in building effective interpersonal relationships and professional networks. These skills are vital in a healthcare setting where teamwork, collaboration, and communication are paramount (Rosen et al., 2018; Keller et al., 2024). A healthcare professional who is emotionally intelligent and empathetic is more adept at establishing rapport with patients, understanding their needs, and collaborating effectively with colleagues.

Moreover, self-realization contributes to the development of symbolic capital, which is reflected in the prestige and recognition one receives within the medical field. A deep sense of professional identity, cultivated through self-awareness and reflection, is highly valued in medicine (Sarraf-Yazdi et al., 2021; Wan et al., 2024). Medical professionals who demonstrate a strong sense of self, adherence to professional standards, and commitment to ethical practices are often held in high regard and are more likely to advance in their careers.

In summary, the interplay of self-realization with the forms of capital outlined by Bourdieu provides a nuanced perspective on its benefits for medical education and career paths. PMR, as a tool for fostering self-realization, enhances the complexity and depth of medical training, thereby facilitating a transformative impact on the careers and lives of future medical professionals. This holistic approach to education not only prepares students for the technical aspects of medicine but also equips them with the emotional and cognitive skills necessary to excel in the rapidly evolving field of healthcare.

### 4.3 Theme: liberation

#### 4.3.1 The intersection of emotional emergence and somatic experiences in medical students

##### 4.3.1.1 Emotional emergence through the practice of PMR

The theme of “liberation,” as captured in Response 1, illustrates the concept of voluntary relinquishment of control and the emotional ramifications stemming from such a deliberate release. This notion is well-supported in the literature (Merluzzi and Philip, 2017). In the context of this study, Response 1 vividly portrays the sense of unburdened freedom that medical students experience through the practice of PMR. This technique provides a brief respite from the relentless demands of their rigorous academic schedules and the pressures of performance. For instance, a medical student, overwhelmed by back-to-back classes, hospital rotations, and academic responsibilities, might find PMR a valuable tool for attaining a momentary sense of release, easing the mental and emotional load associated with their demanding routine (Waqas et al., 2015; Thun-Hohenstein et al., 2021).

##### 4.3.1.2 Somatic manifestations in the stressful journey of medical education

The journey through medical education is fraught with various stressors, including concerns about accumulating debt, financial uncertainties, the drive for success, and anxiety about future prospects (Linn and Zeppa, 1984; Santen et al., 2010; Chang et al., 2012; Hill et al., 2018; Moldt et al., 2022; Li et al., 2023; Muaddi et al., 2023). These constant sources of stress lead to somatic complaints and a cumulative physiological and psychological toll on students as they strive to balance their personal lives with academic commitments (Behere et al., 2011; Picton, 2021; Avila-Carrasco et al., 2022). Notably, medical students often begin their academic pursuits with psychological profiles comparable to, or even stronger than, their non-medical peers. However, the intense demands of their studies can significantly heighten their distress levels (Brazeau et al., 2014; Dyrbye et al., 2014; Pavlinac Dodig et al., 2023; Ramachandran et al., 2023; Zuniga et al., 2023).

These instances underscore the necessity and relevance of incorporating practices like PMR into medical education. By doing so, institutions can provide students with valuable tools for managing the unique challenges they face, thereby contributing positively to their overall wellbeing and effectiveness as future healthcare professionals. The integration of such practices not only supports students in managing the immediate stressors of their education but also prepares them for the enduring demands of a career in healthcare, promoting long-term resilience and professional success (Edmonds et al., 2023).

#### 4.3.2 Integrating PMR in medical education: enhancing learning and wellbeing

##### 4.3.2.1 The therapeutic potential of PMR in accordance with Gagne's instructional design model

In the high-pressure environment of medical education, interventions like Progressive Muscle Relaxation (PMR) hold significant potential. They offer an effective means for students to relinquish control over worries beyond their influence, thereby fostering a sense of calmness, mental peace, and ultimately, enjoyment. This emotional shift, particularly highlighted in Response 1, aligns with Steps 6 and 9 of Gagne's instructional design model (Table 1). These steps emphasize the importance of feedback and self-analysis in the learning process, demonstrating how students reflect on the impact of PMR on their personal and academic lives. For instance, a medical student might use PMR to manage the anxiety of upcoming exams or clinical rotations, finding that this practice not only helps in immediate stress relief but also contributes to a more profound sense of enjoyment and fulfillment in their studies.

##### 4.3.2.2 Personal development and self-care: evolving priorities in medical education

Historically, the culture of medical training has often placed minimal emphasis on self-care and personal development. However, recent global challenges, such as the COVID-19 pandemic, have catalyzed a positive shift toward integrating mindful practices and nurturing activities into the medical curriculum (Dyrbye and Shanafelt, 2012; Cairns and Murray, 2015; Dobkin and Hassed, 2016; Williams et al., 2022). This paradigm shift acknowledges the critical role of self-care in shaping a well-rounded medical practitioner. Practices like PMR can further encourage students to question the excessive merging of their personal and professional identities. By promoting self-realization, PMR helps students recognize the transient nature of such identity constructs (Fulton, 2005). For example, a medical resident grappling with the stress of long shifts might find that PMR not only provides immediate stress relief but also fosters a healthier perspective on balancing personal identity with professional responsibilities.

These sections highlight the profound impact that integrating PMR into medical education can have on students' learning experiences and overall wellbeing. By addressing both the immediate stressors of medical training and fostering a culture that values self-care and personal growth, PMR can play a vital role in shaping more resilient, mindful, and emotionally intelligent future healthcare professionals.

##### 4.3.2.3 Enhancing medical education through volitional liberation and self-control: a framework-aligned approach

The concept of “volitional liberation,” closely associated with self-regulation, plays a pivotal role in medical education, referring to the management of personal responses—emotions, thoughts, behaviors, and impulses—to attain desired goals (Peters et al., 2011). In this study, 'liberation' is interpreted as the students' active choice to regulate their mental, emotional, and behavioral patterns, a skill sharpened by the insights gained from PMR exercises. The student narratives reveal an awakening to new abilities for change, previously considered unattainable, underscoring the empowering influence of PMR.

Professionalism, a cornerstone of medical education, is consistently highlighted across various competency frameworks, including the ACGME, Scottish Doctor Framework, CanMEDS Physician Competency Framework, GMC UK Competency Framework, and Brown Abilities Competency Framework (Figure 3). These frameworks evaluate professionalism through observable behaviors rather than just attitudinal dispositions, focusing on confidentiality, integrity, punctuality, adept management of patient and peer relationships, and adherence to ethical and legal standards (Rees and Knight, 2007).

The cultivation of volitional liberation, intricately linked with self-control and self-regulation, is essential for exhibiting these professional behaviors and is crucial for effective functioning in diverse medical settings (Faisal, 2023). A stronger sense of liberation is associated with improved academic performance, better mental health, and more effective interpersonal relationships (Tangney et al., 2004). Professional accountability is deeply connected to individual liberation (Baumann et al., 2014), a trait vital for medical professionals. Furthermore, medical students are encouraged to develop autonomy, confidence, a propensity for lifelong learning, and intrinsic motivation (Kusurkar and Croiset, 2015; Ganotice et al., 2023).

Incorporating liberation-focused practices like mindfulness and PMR exercises into medical curricula could significantly enhance a wide range of competencies (Dysart and Harden, 2022). For example, these practices could foster communication skills, enabling doctors to empathize with patients, develop critical thinking for better diagnostic abilities, cultivate ethical decision-making for complex situations, and promote self-directed learning for continuous education. Additionally, nurturing a sense of liberation can better prepare students for professional success, equipping them to provide high-quality patient care and meet the standards set by these competency frameworks. This integrated approach, which simultaneously fosters personal growth and skill development, can create a more enriched and holistic medical education environment, addressing both the psychosocial needs of medical students and the required learning outcomes in medical education.

##### 4.3.2.4 PMR as a catalyst for resilience and adaptation in medical education during and beyond the COVID-19 era

The COVID-19 pandemic has undeniably transformed medical education, amplifying existing stressors and compelling a shift to new learning methods. In this context, the role of PMR and its potential for fostering liberation becomes increasingly pertinent. Utilizing psychological theories, we can dissect how PMR-induced liberation enhances medical education, equipping future doctors to better handle the challenges of their profession.

Firstly, Cognitive Load Theory highlights the limitations of our working memory, which juggles new and stored information (Leppink and Van Den Heuvel, 2015; Fredericks et al., 2021). The relentless influx of information in medical education, intensified by pandemic-related stressors, risks overwhelming this system, impeding learning, and risking cognitive burnout. PMR, by alleviating cognitive and emotional stressors, may reduce extraneous cognitive load. This could improve learning efficiency and optimize cognitive resource utilization (Debue and Van De Leemput, 2014; Ghanbari et al., 2020; Kastaun et al., 2021). For instance, a medical student grappling with complex diagnostic protocols could find that regular PMR practice helps manage the cognitive overload, making learning more manageable and effective.

Secondly, Self-Determination Theory posits that fulfilling basic psychological needs—autonomy, competence, and relatedness—is essential for optimal functioning and wellbeing (Ntoumanis et al., 2021). PMR can be instrumental in satisfying these needs. By offering relief from overwhelming stress and promoting mindful engagement with personal experiences, PMR can heighten autonomy, enhancing students' control over their emotional and cognitive states. The tranquility and mastery gained through PMR boost feelings of competence, while shared experiences of stress and relaxation among peers foster a sense of relatedness, nurturing personal and academic growth. A medical student might use PMR to gain a greater sense of control and confidence in clinical settings, thereby improving both their learning experience and interpersonal interactions.

Thirdly, PMR encourages a mindful approach toward personal and academic experiences, creating a psychological environment conducive to resilience and adaptive coping. This liberation from unrelenting academic pressures and self-imposed expectations can lead to better emotional regulation, mental clarity, and cognitive flexibility—crucial skills for navigating the medical profession in the intricate post-COVID landscape.

In conclusion, PMR-induced liberation holds significant potential for enhancing medical education by reducing cognitive overload, fostering self-determination, and promoting mindfulness. By equipping future doctors with psychological resilience, emotional regulation, and cognitive adaptability, PMR can be a pivotal tool in preparing them for the dynamic challenges of their evolving professional field.

##### 4.3.2.5 Embracing liberation in medical education: a Bourdieu-inspired transformation

The concept of liberation within medical education, both as an emergent phenomenon and an instructional objective, holds considerable significance. It addresses the strenuous demands and persistent stressors faced by medical students, such as relentless academic commitments and rigorous performance pressures (Linn and Zeppa, 1984; Santen et al., 2010; Chang et al., 2012; Hill et al., 2018). Pierre Bourdieu's theoretical framework, particularly his concepts of habitus, field, and symbolic capital, offers profound insights into this complex dynamic (Schinkel, 2015; Nairn and Pinnock, 2017).

In Bourdieu's framework, habitus represents the deeply ingrained dispositions, thought patterns, and behaviors developed over time, influencing an individual's perceptions and actions within their social field (Turnbull et al., 2019; Quaye and Pomeroy, 2022). In the hierarchical and competitive landscape of medical education, students' habitus evolves to reflect resilience, diligence, and an unceasing drive for knowledge and clinical expertise (Brazeau et al., 2014; Dyrbye et al., 2014). However, this constant striving often leads to psychological and physiological strain (Behere et al., 2011; Picton, 2021), highlighting the necessity for liberation—a release from these entrenched patterns—to preserve students' wellbeing and mental strength (Waqas et al., 2015).

Practices like PMR offer a vital counterbalance to these habitual tendencies, promoting mental tranquility, enjoyment, and a sense of release from unrelenting academic demands (Fulton, 2005). This demonstrates the potential for medical students to transcend the constraints of their field, leading to a transformation of their habitus (Stokes and McLevey, 2016).

Additionally, Bourdieu's concept of symbolic capital, which refers to the prestige or recognition within a specific field, plays a crucial role in shaping an individual's status (Gilleard, 2020). In medical education, symbolic capital is often linked to academic achievements, research contributions, or clinical prowess. While these aspects are essential for professional growth, the cultivation of liberation—the ability to let go of uncontrollable concerns—may represent a critical yet often overlooked form of symbolic capital. Acquiring this form of capital can result in a positive shift in students' habitus, fostering personal growth, psychological wellbeing, and resilience against the inherent stressors of their field (Dyrbye and Shanafelt, 2012; Cairns and Murray, 2015; Dobkin and Hassed, 2016).

In summary, viewing the concept of liberation through Bourdieu's theoretical lens enhances our understanding of the transformational dynamics in medical education. It underscores the importance of integrating practices that promote liberation, aiming not only for academic excellence but also for the psychological and emotional wellbeing of future medical professionals (Bourdieu, 1990). This holistic approach is crucial in preparing medical students for the multifaceted challenges of their future careers, ensuring they are well-equipped both intellectually and emotionally.

### 4.4 Theme: awareness

#### 4.4.1 Somatic observance via PMR

***Response 1*** within this thematic exploration underscores the crucial aspect of somatic awareness that is inherent to the PMR technique. This process involves directing focused attention to physical sensations in various parts of the body. Engaging regularly in this detailed somatic scanning, combined with a committed effort toward achieving relaxation, plays a significant role in preemptively addressing secondary symptoms commonly associated with stress, such as fatigue and pain (Hussain and Said, 2019; Izgu et al., 2020).

The findings align with the concept that the frequent practice of PMR exercises can cultivate an adaptive response to both physical and psychological challenges. This adaptability functions as an effective mechanism for maintaining holistic health (Selmi et al., 2023). For instance, a medical student or healthcare professional, constantly subjected to high-stress environments, may find that regular PMR sessions help in mitigating the onset of stress-related symptoms. Through PMR, they develop a heightened awareness of their bodily responses to stress, enabling them to manage these symptoms more effectively.

Moreover, this enhanced somatic awareness can lead to better overall health management. By becoming more attuned to the signals their body sends in response to stress, individuals can take proactive steps to address these symptoms early on, preventing the exacerbation of stress-related conditions. This preventive approach is particularly beneficial in professions like medicine, where long hours and high-pressure situations are common.

In conclusion, the practice of PMR and its emphasis on somatic awareness can be a valuable tool in the arsenal of medical students and healthcare professionals. It not only aids in stress management but also contributes to the development of a more adaptive response to physical and psychological demands, promoting holistic wellbeing. This approach is especially pertinent in the demanding field of healthcare, where managing one's health is crucial for both personal wellbeing and the delivery of high-quality patient care.

#### 4.4.2 Managing anger through PMR: enhancing mindfulness and emotional regulation in medical education

***Response 2*** in this study focuses on managing the emotion of anger and its physical manifestations, a critical aspect of emotional intelligence in medical training. Empirical research, including work by Kwak et al. (2020), has demonstrated the effectiveness of PMR in not only alleviating symptoms of anger but also addressing conditions arising from its prolonged repression. In the context of medical education, one of the key goals of PMR is to enhance students' ability to remain present in the current moment, thereby fostering both mental and physical relaxation.

This response indicates a significant improvement in students' awareness, prompting them to actively pursue relaxation. This aligns with the Buddhist principle that the body is the primary site for developing mindfulness (Analayo, 2020). The application of PMR in this setting underscores its potential to cultivate embodied mindfulness, enabling students to experience grounding and centeredness (Analayo, 2020).

For example, a medical student encountering high levels of stress and frustration during their studies or clinical rotations might use PMR as a strategy to manage these challenging emotions. By engaging in deep breathing and focusing on bodily sensations, the student can initiate relaxation, effectively reducing feelings of anger. This approach, rooted in increased mindfulness awareness, demonstrates the practical application of PMR in mitigating emotional reactions (Matsumoto and Smith, 2001).

The significance of this finding extends beyond the individual benefits to the broader field of medicine and healthcare. Emotional regulation, particularly in managing anger, is crucial for medical professionals who often face high-pressure situations and complex patient interactions. The ability to remain composed and make decisions without the interference of strong emotions like anger is essential for effective patient care and professional conduct.

In summary, incorporating PMR into medical education can be a powerful tool for developing emotional regulation skills, particularly in managing anger. This practice not only benefits the personal wellbeing of medical students but also enhances their professional competencies, preparing them to navigate the emotional complexities of the healthcare environment effectively.

#### 4.4.3 Enhancing self-awareness and competency in medical education through PMR

***Responses 1 and 2*** under the theme of “Awareness” collectively indicate a significant improvement in students' introspective and emotional acuity, a result of consistent practice of the Progressive Muscle Relaxation (PMR) exercise. This practice not only cultivates an enhanced self-awareness but also fosters critical self-reflection, essential in the development of medical students. Such introspection is pivotal for cultivating vigilance toward evidence-based decision-making and personal error recognition (Epstein, 1999), aspects that are crucial in the clinical setting.

The ability to engage in compassionate and humane patient interactions is deeply connected to the recognition and acknowledgment of one's internal emotional and cognitive states (Elder et al., 2007). In medical practice, this enhanced perceptual capacity is invaluable, particularly during the clerkship years of undergraduate medical education, where competency is a primary focus (Benbassat and Baumal, 2007). For instance, a medical student who has developed greater emotional acuity through PMR might be more attuned to patients' needs and more capable of empathetic engagement, thereby improving the quality of patient care.

Furthermore, ***Responses 1 and 2*** highlight the effective implementation of PMR exercises, aligning with the guidance of Gagne's Instructional Design Model's fifth step. This step involves providing learning guidance, which in the context of PMR, means instructing students on how to effectively engage in relaxation techniques. These responses also validate Gagne's ninth step, which emphasizes the importance of enhancing learning through the practical application of skills. In the case of PMR, this step is realized as students apply relaxation techniques to manage their internal states, thereby enhancing their emotional and introspective awareness.

The findings from these responses have broader implications for the field of medicine and healthcare. Developing introspective and emotional acuity is crucial for medical professionals, as it directly impacts their ability to make sound decisions, recognize personal biases or errors, and engage empathetically with patients. By incorporating PMR and similar practices into medical education, institutions can nurture a generation of healthcare professionals who are not only technically proficient but also emotionally intelligent and self-aware, attributes that are increasingly recognized as essential in the delivery of high-quality healthcare.

#### 4.4.4 Integrated enhanced awareness in medical education: alignment with learning outcomes frameworks

Recognizing the critical role of awareness in medical education requires a comprehensive understanding from various theoretical perspectives (Figure 3). Cognitive psychology views awareness as a metacognitive ability involving introspective self-evaluation and regulation (Flavell, 1979). Psychoanalytic theory relates it to the recognition of unconscious emotions and experiences (Freud et al., 1961). Social constructivism interprets it as an understanding of one's sociocultural environment (Vygotsky and Cole, 1978), while humanistic psychology sees it as holistic self-understanding.

Enhanced awareness could significantly impact the six core competencies outlined by the ACGME framework (Nasca et al., 2012). A heightened self-awareness can improve patient care through increased empathy. A comprehensive self-understanding can lead to better communication and professionalism. Awareness of learning strengths and weaknesses aligns with practice-based learning, and understanding one's role in healthcare systems supports systems-based practice.

The Scottish Doctor Framework (Ellaway et al., 2007) identifies key outcomes, including the roles of a scholar, practitioner, and professional. Awareness can contribute to these roles by helping identify personal biases in scientific interpretation, recognizing gaps in skills or knowledge, and enhancing decision-making through an understanding of personal values and ethics.

The CanMEDS Framework (Thoma et al., 2023) defines seven roles for competent physicians. Self-awareness contributes significantly to the 'Scholar' role by recognizing learning needs and to the 'Professional' role by deepening the understanding of professional identity. In the 'Health Advocate' role, awareness of sociocultural health determinants is essential.

The GMC UK Competency Framework (Southgate et al., 2001) emphasizes good clinical care, medical practice maintenance, patient relationships, and teamwork. Enhanced self-awareness can improve clinical care by recognizing personal limitations, aid continuous learning for maintaining medical practice, and enhance communication and teamwork skills necessary for effective patient and colleague relationships.

In the Brown Abilities Competency Framework (Batt et al., 2021), awareness plays a key role across various competencies, including learner self-assessment, cognitive integration, clinical judgment, and professionalism. Awareness assists in recognizing personal strengths, weaknesses, and learning needs, understanding cognitive processes and biases, improving clinical decision-making, and aligning with ethical values and principles.

Integrating the enhancement of “awareness” within these competency frameworks could lead to a transformative shift in medical education. It has the potential to develop more insightful, empathetic, and effective medical practitioners, equipped with a comprehensive understanding of themselves, their patients, and the healthcare environment. This holistic approach to medical training emphasizes not only technical expertise but also the personal and interpersonal competencies necessary for successful medical practice.

#### 4.4.5 The enhancement of medical education via awareness through PMR: implications for the COVID era and beyond

In the educational landscape reshaped by the COVID-19 pandemic and its aftermath, the integration of awareness into pedagogical strategies has become essential. The global crisis has significantly altered the learning environment, necessitating the adoption of innovative and adaptable teaching methodologies. Introducing awareness-building techniques such as PMR into the curriculum can profoundly impact learning outcomes.

Firstly, the disruption of traditional learning environments due to COVID-19, with a sudden pivot to remote learning, has highlighted the necessity for learners to possess a strong internal guide to effectively navigate this new landscape. This is where self-awareness plays a pivotal role. Students with enhanced self-awareness are better equipped to regulate their learning pace, identify personal areas for improvement, and adapt strategies for optimal educational outcomes, aligning with the principles of self-regulated learning theory (Panadero, 2017).

Moreover, the pandemic has amplified the levels of stress and anxiety experienced by students. By fostering emotional self-awareness, learners gain deeper insights into their emotional states and develop adaptive coping strategies, in line with Emotional Intelligence Theory (Izzarelli, 2022). This awareness is crucial for maintaining mental wellbeing in the face of unprecedented challenges.

Additionally, according to Vygotsky's Socio-cultural Theory (Vygotsky et al., 1987), learning is inherently a socially mediated process. In the era of digital learning, cultivating social awareness is vital for students to engage effectively in collaborative learning activities, interact with diverse viewpoints, and foster cohesive learning communities.

The existential nature of the pandemic also emphasizes the importance of critical awareness or conscientization (Freire, 1973). This form of awareness encourages learners to critically engage with and understand the socio-political realities that shape their educational experiences, leading to a more comprehensive and holistic learning process.

In conclusion, the integration of various forms of “awareness” into educational strategies during and after the COVID-19 pandemic is not just an enhancement but a fundamental shift in pedagogical approach. By promoting self, emotional, social, and critical awareness, educational institutions can nurture resilient learners adept at navigating the complexities of the changed educational landscape (Fu and Zhang, 2024). This approach sets the foundation for a more adaptive, responsive, and holistic pedagogy in the post-COVID era, preparing students not just academically, but also for the multifaceted challenges of the modern world.

#### 4.4.6 The interplay of Bourdieu's Theory of Practice and awareness

From a sociological perspective, Bourdieu's Theory of Practice offers a nuanced understanding of the interplay between individuals and their social environments, particularly in the context of medical education and career progression (Rhynas, 2005). This theory delineates a dynamic relationship between the individual (the agent) and their social structures (the field), mediated through a complex interplay of 'habitus', 'capital', and 'field'. Applying this framework to the impact of PMR on medical education and practice reveals how it can significantly influence both individual dispositions and broader societal structures.

PMR, by enhancing awareness and control, cultivates a distinctive “habitus” within medical students and practitioners. This newly acquired habitus, characterized by skills in mindfulness, emotional regulation, and stress management, forms an adaptive internal schema. It shapes how individuals interact within the challenging field of medical education and healthcare practice, thereby subtly altering the dynamics of the field itself. For example, a medical student or practitioner who practices PMR may develop a more balanced approach to handling the stresses of their profession, impacting their interactions with colleagues and patients.

Furthermore, the competencies and resilience developed through PMR can be seen as a form of embodied 'capital'. This capital is a valuable resource that not only enhances individual capacities for learning and practice but also possesses the transformative potential to influence social interactions, professional networks, and career trajectories within the medical field. For instance, a physician who has honed their emotional regulation skills through PMR is likely to navigate patient interactions and professional challenges more effectively, enhancing their reputation and standing in the medical community (Guo et al., 2023).

Viewing the integration of PMR in the medical curriculum through Bourdieu's theoretical lens reveals it to be more than just an educational strategy or stress management technique. It emerges as a powerful agent of sociological change, shaping individual dispositions and the larger structures of medical education and practice. By empowering individuals to modify their habitus and capital through PMR, we initiate a cascade of changes that extend to the broader field of medical education and healthcare.

### 4.5 Theme: interpersonal relationships

#### 4.5.1 Shifting perspectives and reinforcing interpersonal relationships: lessons from PMR

PMR has demonstrated significant potential in altering personal perspectives and enhancing self-awareness, a transformation echoed in Response 1 (Liu et al., 2020; Xiao et al., 2020). This practice enables medical practitioners and students to release persistent thoughts and effectively apply these insights into their daily interactions, aligning with the objectives of knowledge utilization and transfer in Gagne's instructional design model (Table 1). These shifts toward greater self-awareness have profound implications for interpersonal relationships within medical settings (Lee et al., 2023).

For instance, consider a medical student who practices PMR regularly. The relaxation and mindfulness cultivated through PMR can lead to an increased sense of empathy and understanding. This enhanced emotional intelligence is crucial in a clinical setting, where interacting with patients requires sensitivity to their emotional and physical needs. A student who is more self-aware is likely to be more attuned to the nuances of patient communication, leading to more effective and compassionate care.

Similarly, in a healthcare team, professionals who engage in PMR might find improvements in their communication with colleagues. The stress management benefits of PMR can result in a calmer demeanor, facilitating clearer and more constructive interactions. This is particularly important in high-stress environments like hospitals, where clear communication and teamwork are essential for patient safety and effective care.

Moreover, PMR can also aid in managing the dynamics of the mentor-mentee relationship in medical education. For educators and mentors, practicing PMR could lead to a more patient and understanding approach toward students, nurturing a more supportive and conducive learning environment (Beck Dallaghan et al., 2022).

In essence, the practice of PMR contributes to the development of key interpersonal skills such as empathy, active listening, and clear communication. These skills are integral to building meaningful connections, whether it be with patients, colleagues, or within the educational context (Kourkouta and Papathanasiou, 2014). By incorporating PMR into the medical curriculum and encouraging its practice among healthcare professionals, there is an opportunity to significantly enhance the quality of interpersonal interactions across various aspects of medical practice. This, in turn, can lead to improved patient outcomes, more cohesive healthcare teams, and a more supportive educational environment (Sharkiya, 2023).

#### 4.5.2 PMR as a tool for emotional regulation and interpersonal relationship enhancement in medical settings

The transformative impact of PMR in emotional management is vividly illustrated in Response 2 (Contrada and Baum, 2010; Andolhe et al., 2015; Cepeda-Lopez et al., 2023). This response highlights a transition from anger to tolerance, showcasing PMR's effectiveness in helping individuals not only recognize but also regulate their emotions. This skill of emotional regulation, a key aspect of emotional intelligence, plays a vital role in maintaining healthy and productive interpersonal relationships, by reducing conflict and promoting understanding.

In the context of medical practice, the ability to control one's emotions is not only beneficial on a personal level but also essential professionally. For healthcare professionals, managing emotions effectively is crucial for building trust and enhancing therapeutic relationships with patients (Van Zanten et al., 2007; Whisman, 2013). For example, a physician who practices PMR might find themselves better equipped to handle emotionally charged situations with patients, responding with empathy and composure rather than frustration or irritation. This can significantly improve the quality of patient care, as patients are more likely to feel heard, understood, and respected in such interactions.

Additionally, emotional regulation fostered through PMR can positively impact teamwork and collaboration within healthcare settings. Medical professionals who can manage their emotions effectively are more likely to contribute to a positive work environment, engage in constructive communication, and resolve conflicts amicably (Louwen et al., 2023). This is particularly important in high-stress situations, where clear and calm communication is essential for patient safety and effective teamwork.

In medical education, teaching students and trainees the practice of PMR can equip them with essential tools for emotional regulation. This preparation is invaluable for future interactions with patients and colleagues, enabling them to navigate the emotional complexities of the medical profession with greater resilience and understanding.

In summary, incorporating PMR into the routine of medical professionals and students offers significant benefits for interpersonal relationships in medical settings. By enhancing emotional regulation, PMR can lead to more empathetic patient interactions, improved teamwork, and an overall more supportive healthcare environment. This approach not only benefits individual practitioners but also contributes to the broader goal of delivering compassionate and effective healthcare.

#### 4.5.3 Enhancing coping mechanisms and interpersonal relationships through PMR in medical education

The efficacy of PMR in improving coping mechanisms is highlighted in both Response 1 and Response 2 (Troxel et al., 2010; Steinberg et al., 2016; Tamminga et al., 2023). PMR facilitates emotion-focused coping, which helps modify emotional reactions to stressors, thus building resilience. This aspect is especially crucial for medical students, who often face continuous stress and fatigue, potentially leading to a decline in their coping abilities over time (Andolhe et al., 2015). By offering a method to alleviate negative emotions such as fear, anger, and tension, PMR not only enhances individual emotional health but also positively impacts the dynamics and quality of interpersonal relationships (Baldacchino and Draper, 2001; Xiao et al., 2020; Seid et al., 2023).

In the context of medical education, PMR's role in fostering effective coping mechanisms can significantly influence peer-learning and collective intelligence building. For example, a medical student who regularly practices PMR might find themselves more capable of engaging in group discussions and collaborative learning activities with a calmer and more focused mindset. This enhanced emotional regulation can lead to more productive interactions and a more conducive learning environment, where ideas are shared and discussed openly and constructively.

Moreover, the practice of PMR can be particularly beneficial in clinical settings, where teamwork is essential. Medical practitioners who are adept at managing their stress and emotions can contribute to a more harmonious and effective team dynamic. In situations where quick decision-making and collaborative efforts are required, the ability to remain composed and clear-headed is invaluable.

Additionally, PMR can aid in developing a culture of collective intelligence within healthcare teams. When individuals are better equipped to manage their stress and emotions, they are more likely to participate actively in discussions, offer insightful contributions, and be open to others' perspectives. This collaborative approach not only enhances the learning experience for medical students but also improves patient care by combining diverse expertise and viewpoints (Kurvers et al., 2023).

In summary, the practice of PMR in medical education extends beyond individual benefits, fostering improved coping mechanisms, and emotional regulation that are crucial for effective interpersonal relationships and collaborative learning. By integrating PMR into the curriculum, medical institutions can cultivate a more resilient, empathetic, and collaborative future healthcare workforce, capable of navigating the challenges of the medical profession with greater ease and effectiveness.

#### 4.5.4 Impact of PMR on interpersonal relationships

The transformative effect of PMR on interpersonal relationships, particularly between medical students, healthcare professionals, and patients, is evident in both responses. As medical students and professionals experience emotional transformation through PMR, their ability to build and maintain relationships, a critical aspect of clinical practice, is significantly enhanced (Zhou et al., 2015; Jassim et al., 2023).

In the context of medical training, such as during residency, ward rounds, or clinical rotations, effective interpersonal skills are paramount. For instance, a medical resident who practices PMR may find themselves better equipped to manage the stresses of patient interactions. This emotional regulation can lead to improved communication with patients, fostering a deeper understanding of their concerns and needs. In turn, this understanding can enhance diagnostic accuracy and the effectiveness of therapeutic instructions, contributing to a stronger patient-provider relationship (Van Zanten et al., 2007).

Moreover, the trust established through these improved interactions creates an environment where patients feel comfortable expressing their emotions and concerns. This openness allows medical professionals to better comprehend the patients' needs and expectations, leading to more personalized and effective care (Whisman, 2013). For example, during patient consultations, a healthcare professional who has honed their emotional intelligence through PMR might more accurately interpret a patient's non-verbal cues and emotional state, facilitating a more empathetic and effective response.

In healthcare settings, the quality of medical service delivery is intimately linked to the quality of interpersonal relationships. Medical professionals who are adept at managing their emotions and who possess strong interpersonal skills are likely to provide higher quality care. This not only benefits patients but also contributes to a more positive work environment for healthcare teams.

In conclusion, the practice of PMR can play a vital role in enhancing the interpersonal skills of medical students and healthcare professionals (Amukugo et al., 2020). By fostering emotional regulation and empathy, PMR enables these individuals to engage more effectively with patients and colleagues. This improvement in interpersonal relationships is crucial for the delivery of high-quality healthcare and for the overall functioning of healthcare systems. By integrating PMR into medical education and ongoing professional development, the healthcare industry can ensure a more empathetic, responsive, and patient-centered approach to care.

#### 4.5.5 Enhancing patient-centered competencies: insights from international medical competency frameworks

PMR significantly contributes to the development of key competencies outlined in various medical education frameworks, including the ACGME, the Scottish Doctor Framework, the CanMEDS Physician Competency Framework, the GMC UK Competency Framework, and the Brown Abilities Competency Framework (Figure 3). Its influence is particularly notable in enhancing interpersonal relationships, especially in patient and community interactions, within both medical education and practice.

In medical education, PMR serves as an essential tool for fostering self-awareness, emotional control, and resilience, aligning with the goals of these frameworks to develop holistic, emotionally intelligent physicians (Farahmand et al., 2022). These skills are critical for personal and professional development, as they enable medical students and professionals to manage stress effectively, understand their emotional responses, and maintain a balanced state of mind. For example, the ACGME framework highlights the importance of personal growth and self-care within its professionalism and personal development competencies, and PMR plays a significant role in nurturing these attributes.

In clinical practice, particularly in patient interactions, PMR's impact on interpersonal relationships is crucial. It enhances physicians' ability to understand and manage their emotions, contributing to improved communication and collaboration with patients. This emotional intelligence is vital for building trust with patients, enhancing therapeutic relationships, and improving the overall quality of patient care. Additionally, frameworks like the Scottish Doctor and Brown Abilities emphasize the importance of effective communication and empathetic understanding in patient care. PMR's role in developing these competencies highlights its broader significance in training physicians to deliver high-quality, empathetic, and patient-centered care.

Furthermore, PMR not only nurtures necessary skills within individuals but also promotes a supportive and empathetic culture within the medical community. The integration of PMR into both medical education and practice fosters the development of physicians who are not only skilled clinicians but also emotionally intelligent professionals. This holistic approach to medical training prepares physicians to manage interpersonal relationships with patients and communities effectively, ensuring a comprehensive approach to healthcare delivery. In summary, incorporating PMR into medical training and practice is essential for cultivating a more resilient, adaptive, and proficient medical community capable of navigating the complexities of modern healthcare.

#### 4.5.6 Interpersonal relationship enhancement through PMR: a pedagogical shift during the COVID-19 era and beyond

The COVID-19 pandemic catalyzed a significant transformation in medical education, driving the shift toward virtual learning platforms and self-directed learning approaches. In this new educational context, the practice of PMR stands out for its focus on enhancing interpersonal relationships, offering a distinct advantage. Anchored in Social Learning Theory (Bandura, 2001), which suggests that learning occurs through observation, imitation, and modeling, PMR emphasizes self-awareness and emotional regulation as key components for coping and stress management. This aligns well with the theory, providing a practical model for students to emulate.

Moreover, effectively applying PMR requires a deep understanding of interpersonal dynamics, which resonates with Vygotsky's Socio-Cultural Theory (Vygotsky et al., 1987). This theory stresses the importance of social context in learning processes. PMR, therefore, opens avenues for medical students to engage in collaborative learning, enhancing their empathy, communication skills, and emotional intelligence—all crucial traits in the medical field.

From a psychological standpoint, Maslow's Hierarchy of Needs (Hale et al., 2019) posits that the psychological wellbeing of individuals depends on satisfying basic physiological and safety needs. Amid the challenges posed by the COVID-19 pandemic, PMR has served as an effective stress management tool, helping to fulfill these foundational needs, and thus creating a conducive environment for both learning and personal development.

As we move into the post-COVID era, the competencies honed through PMR are poised to become even more valuable. The pandemic has underscored the need for physicians to be emotionally intelligent professionals, adept at empathizing with patients and managing their emotions, in addition to being skilled clinicians. PMR plays a crucial role in this shift, preparing future doctors for the evolving demands of the medical profession. It underscores the importance of a more holistic approach to medical education, one that encompasses not only clinical skills but also interpersonal competencies crucial for effective medical practice. This broadened focus is essential for training medical professionals who are well-equipped to meet the challenges and complexities of modern healthcare.

#### 4.5.7 Applying Bourdieu's Theory: benefits of interpersonal relationships developed through PMR for medical students and practitioners

Bourdieu's social theory, centered around the concepts of habitus, capital, and field (Rhynas, 2005), offers an insightful framework for examining how PMR fosters interpersonal relationships, benefiting medical students and practitioners.

The 'habitus,' comprising dispositions and inclinations shaped by social experiences, is crucial in how medical students and practitioners perceive and interact within their environments. PMR, with its emphasis on self-awareness and emotional regulation, can significantly influence the 'habitus.' It fosters increased resilience, emotional stability, and empathy, which are essential for navigating the stressful and emotionally charged 'field' of medical practice and education. For example, a medical student who practices PMR may develop a more empathetic approach to patient care, understanding patient concerns more deeply and responding with greater compassion.

Moreover, PMR enhances the “capital”—the skills and competencies medical students and practitioners possess. In this context, 'capital' includes interpersonal competencies like effective communication, understanding, and empathy, which are invaluable in medical practice. These improved interpersonal skills translate into “social capital”—trust and cooperation that are foundational in both doctor-patient relationships and collegial interactions among healthcare professionals. For instance, a healthcare professional adept in PMR might exhibit better teamwork and collaboration skills, contributing to a more harmonious and efficient working environment.

Finally, the 'field' in Bourdieu's theory represents the various social and professional settings in which medical students and practitioners operate. The effective interpersonal relationships cultivated through PMR practice can positively influence these 'fields.' This is achieved by promoting a supportive and empathetic culture within medical communities and enhancing the overall quality of care in healthcare settings. For instance, in hospital wards or clinics, PMR-trained medical staff can create a more patient-centered environment, leading to improved patient satisfaction and outcomes.

In essence, PMR's role in developing interpersonal relationships extends beyond individual benefits, influencing the broader context of medical education and practice. By shaping the “habitus” toward more empathetic and resilient dispositions, enhancing the 'capital' with vital interpersonal skills, and positively impacting the 'field' of medical practice, PMR contributes to creating a more supportive, empathetic, and effective medical community.

### 4.6 Integrative Psychological Resilience Model in Medical Practice

#### 4.6.1 Framework of the model

**Figure 4:**
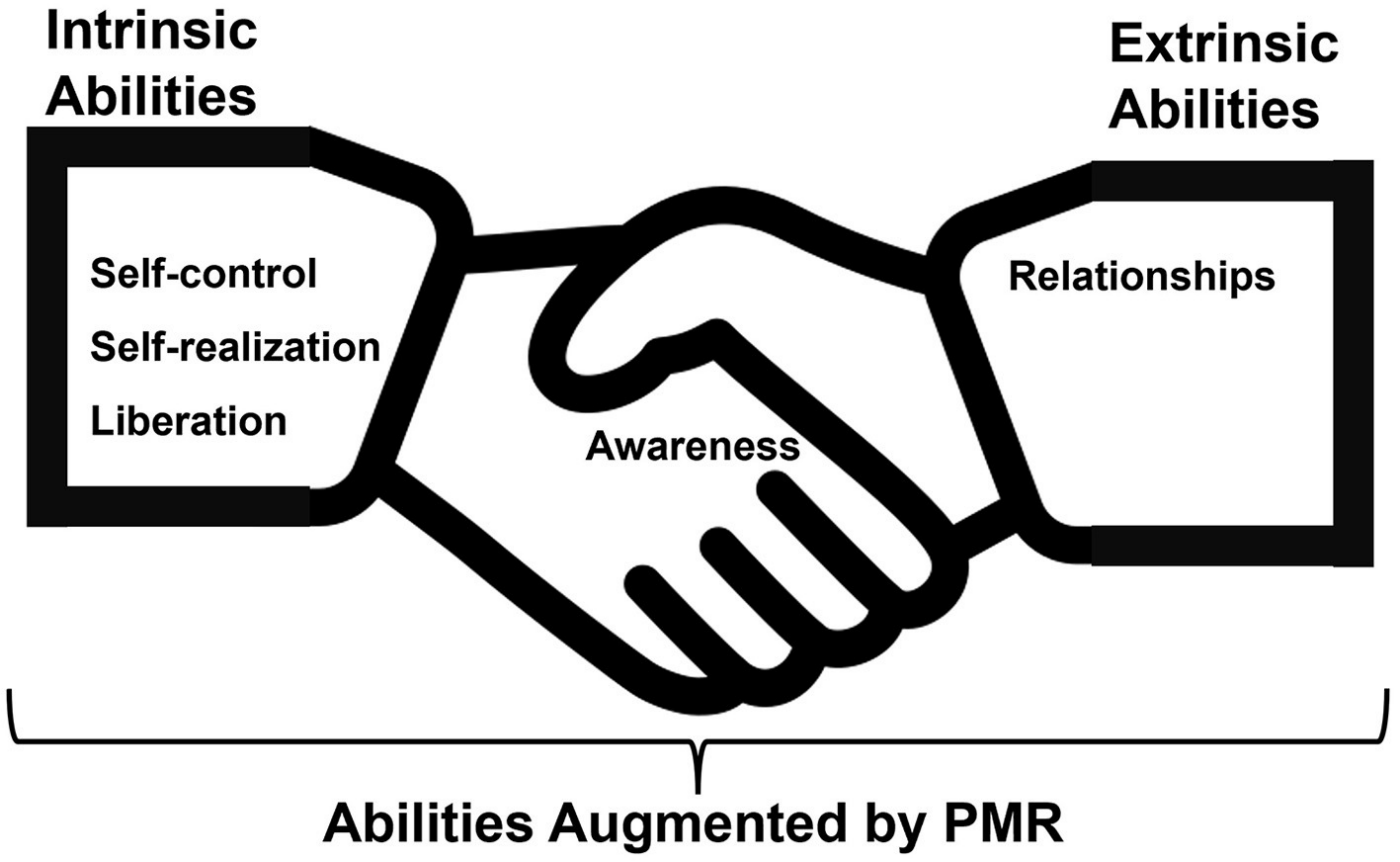
The Integrative Psychological Resilience Model in Medical Practice (IPRMP). The figure illustrates the IPRMP, a novel framework constructed on psychological, learning, and social constructs, including intrinsic abilities such as self-control, self-realization, liberation, awareness, and extrinsic abilities such as interpersonal relationships. PMR, at the heart of the model, functions as a key tool to nurture these traits, enhancing both medical practice and education. The diagram depicts how self-control, rooted in Bandura's Social Cognitive Theory, is augmented through PMR, which strengthens emotional regulation, particularly in stressful medical contexts. The concept of self-realization, influenced by Maslow's Humanistic Theory, signifies understanding and accepting one's abilities, potential, and limitations, and is fostered by the mental clarity afforded by PMR. Liberation, inspired by Cognitive Liberation Theory, signifies the relief from inhibitory beliefs and anxiety, a result of PMR's focus on muscle tension release and relaxation. The model's awareness element is grounded in mindfulness theory and represents a consciousness of one's immediate experience, cultivated through PMR's focus on the physical sensation of relaxation. The model also emphasizes the role of interpersonal relationships, underpinned by Social Interdependence Theory and Bourdieu's social capital concept, showing how reduced stress from PMR facilitates improved interpersonal interactions. The figure highlights the interconnectedness of these components, suggesting how improvements in one area, such as self-control, can lead to enhancements in others, such as self-realization and liberation, subsequently promoting awareness and positively influencing interpersonal relationships. The IPRMP thus provides a comprehensive, integrative approach to bolster resilience and wellbeing in medical professionals through the practice of PMR, promising to reduce burnout, improve patient care quality, and enhance the preparedness of future doctors.

Based on our thematic analysis we propose the Integrative Psychological Resilience Model in Medical Practice (IPRMP) (Figure 4). IPRMP is a novel framework based on an intricate interplay between psychological, learning, and social constructs, specifically self-control, self-realization, liberation, awareness, and interpersonal relationships. It leverages the practice of PMR as a pivotal tool to foster these traits and, in turn, improve medical practice and education. Within the IPRMP, the concept of self-control draws from Bandura's Social Cognitive Theory (Bandura, 2001), where self-efficacy and personal agency are crucial determinants of behavior. PMR can enhance one's self-control, fostering the ability to regulate one's emotional reactions, particularly under stressful medical scenarios.

Self-Realization, on the other hand, refers to an individual's understanding and acceptance of their own abilities, potential, and limitations. Maslow's Humanistic Theory (Hale et al., 2019) underscores the importance of self-realization in reaching one's full potential. PMR aids in attaining self-realization by promoting relaxation, mental clarity, and increased self-awareness, thereby enabling medical practitioners to recognize their strengths and areas for improvement.

Drawing from Cognitive Liberation Theory (McAdam, 2022), liberation in the IPRMP context represents freedom from inhibiting beliefs, anxiety, and self-doubt. PMR, with its focus on muscle tension release and relaxation, could help liberate medical practitioners from the undue psychological burden, leading to enhanced emotional intelligence and wellbeing.

Within the model, awareness is grounded in the mindfulness theory and implies maintaining a moment-to-moment consciousness of one's thoughts, feelings, and surroundings. PMR, through its focus on the physical sensation of relaxation, heightens body awareness, which subsequently amplifies mental mindfulness—an essential trait for precise, empathetic, and effective medical practice.

Drawing on Social Interdependence Theory (Johnson and Johnson, 1989; Shimizu et al., 2020) and Bourdieu's social capital concept (Rhynas, 2005), the model acknowledges the importance of interpersonal relationships within medical settings. PMR's stress-reducing effects may facilitate better interpersonal interactions, fostering collaboration, understanding, and mutual respect among practitioners and patients.

In this model, these traits are not standalone; they are interconnected, influencing, and augmenting each other (Figure 4). For instance, enhanced self-control through PMR can lead to improved self-realization, resulting in liberation from negative mental states. This liberation further cultivates awareness, which positively impacts interpersonal relationships (as indicated in the Figure).

The IPRMP, therefore, offers a multifaceted and integrative approach toward fostering resilience and overall wellbeing in medical practitioners. By leveraging PMR, the model promises to instill crucial psychological traits, mitigate professional burnout, and enhance patient care quality. Simultaneously, its application in medical education can instill these essential skills early, preparing future doctors for the rigors of the profession, and shaping a more empathetic, effective, and resilient medical workforce.

#### 4.6.2 Role of IPRMP in professional development and patient care

The IPRMP, with its focus on PMR and various psychological and social constructs, presents a significant opportunity for enhancing healthcare at various levels. For medical students and healthcare professionals, the IPRMP can serve as a vital tool for personal and professional development.

Medical students, often at the beginning of their stressful and demanding careers, can benefit immensely from the IPRMP framework. By learning and practicing PMR, they can develop stronger self-control, which is crucial for managing the high-pressure situations they will encounter. The self-realization aspect of the model can help them understand their own strengths and limitations, fostering a more grounded and realistic approach to their medical journey. This early integration of the IPRMP in their training can lay a foundation for resilience and emotional intelligence, key attributes for a successful medical career.

For healthcare professionals, the IPRMP offers a way to combat burnout, a prevalent issue in the medical field. The liberation and awareness components can help them break free from mental fatigue and maintain a high level of mindfulness in their practice. This is especially beneficial in enhancing patient care, as a more aware and emotionally intelligent practitioner is likely to be more empathetic and effective in their interactions with patients.

From the perspective of Bourdieu's framework, the IPRMP can be seen as a tool for accumulating cultural and social capital within the healthcare profession. The skills and traits developed through this model—such as improved interpersonal relationships, emotional intelligence, and self-awareness—are valuable forms of capital in the medical field. These skills not only enhance individual practitioners' capability but also contribute to a more collaborative and efficient healthcare system. The model fosters a more supportive and understanding environment, which is crucial for patient care and the overall wellbeing of the medical community.

In essence, the IPRMP has the potential to foster a more resilient, empathetic, and efficient healthcare workforce. By focusing on the holistic development of medical practitioners, it not only benefits the individuals within the profession but also enhances the quality of care provided to patients, contributing to the greater good of the healthcare system as a whole.

### 4.7 Utilizing Mento's change management model to implement PMR in CBMC

Unquestionably, one of the most profound truisms that resonate in the realm of organizational change is captured succinctly by Senge in 'The Fifth Discipline': “We both fear and seek change... People don't resist change. They resist being changed.” (Senge, 1990). This wisdom was instrumental in setting the direction for our journey, as we needed to delineate a robust model or framework to orchestrate the much-needed change in integrating PMR for our CBMC. Addressing Senge's “Universal Challenges” in learning organizations, categorized into the initiating of change, sustaining momentum, and system-wide redesign and rethinking, associated with integrating PMR was the formidable task at hand (Kleiner et al., 1999). Consequently, we focused our attention on four highly-regarded change-management models, namely (1) Schein's Steps of Change (Schein, 2004), (2) Kotter's 8-steps to transform organization (Kotter and Cohen, 2002), (3) Senge's—Challenges of Change (Senge, 1990), and (4) Fullan and Miles' propositions for success (Fullan, 2007).

Using Schein's “unfreezing” as a guiding principle, we critically analyzed the change-management models at our disposal. Kotter's transformative 8-step model emerged as a compelling option due to its capacity to initiate a change of thought or “unfreezing”, create a sense of urgency, build a powerful and guiding coalition, and craft a shared vision. However, on deeper reflection, an omission was perceived in Kotter's model as its underpinnings are firmly rooted in Situational and Contingency Leadership Theories. Contrastingly, an academic institution thrives more effectively under the tutelage of Transactional and Transformational Leadership archetypes. The optimal leadership in such environments is often materialized through an “informal kibitzing” from an expert ensemble or “The Leader Team”. Thus, our search led us to the discovery of Mento's model of change, an intricate tapestry woven with strands from Kotter's model, Jick's model, and GE's model (Fullan, 2007). Although Mento's model may not yet enjoy widespread recognition in the sphere of medical education and healthcare, its successful application in a Fortune 500 defense industry firm speaks volumes of its potential efficacy. Also, we have successfully utilized this model to implement changes in our curriculum previously (Banerjee et al., 2019b; Naidoo et al., 2020). The comprehensive nature of Mento's model, along with its alignment with the leadership style prevalent in academic institutions including MBRU, made it an attractive choice for our study.

Under the guidance of Mento's model, the PMR program can be introduced to faculty and students via a thoughtfully designed and executed faculty development program (Table 3). This program can provide educators with a comprehensive understanding of PMR, its benefits for student wellbeing, and the skills required to teach and practice PMR. Mento's model also emphasizes the need for a step-by-step implementation plan to guide change management (Refer to Table 3 for details). Through this approach, PMR can be systematically and effectively integrated into the CBMC, fostering an educational environment that prioritizes student/healthcare professional mental health. This change, in turn, can potentially lead to improved academic performance, higher student satisfaction, and a more supportive learning environment that equips medical students and healthcare workforce with the skills to manage stress and promote resilience in the post-COVID era.

**Table 3:** Guidance plan showing the activities and timeline corresponding to each step of Mento's Change Model aimed at implementing PMR in Phase I of the MBBS curriculum.

**Step No**.	**Steps of Mento's Model of Change**	**Activity to facilitate/implement the change**	**Timeline**
1	The idea and its context	Preliminary results from this pioneering investigation indicate that PMR augments student wellbeing and resilience. *The idea is to integrate PMR in the phase-I of the MBBS curriculum at MBRU* (Supplementary Table 1)	N/A
2	Define the change initiative	Present to the concerned stakeholders (*student volunteers from phases II and III who will work in collaboration with the PMR instructor and the associated team to disseminate PMR to the Phase I students and ensure compliance*): ⇒ What is PMR? ⇒ Benefits of PMR. ⇒ Successful case-studies of PMR. (*Presentation of observations from this study and those from the literature*)	1 week prior to the commencement of Phase I during the week of student orientation.
3	Evaluate the climate for change	Appraise the necessary resources, prior knowledge of stakeholders and technological know-how required to successfully implement PMR, *through SWOT analysis*.	1 week prior to the commencement of Phase I during the week of student orientation
4	Develop a change plan	Work with the PMR instructor and associated team at MBRU to develop a plan to train the stakeholders regarding strategies to implement PMR in phase I.	1 week prior to the commencement of Phase I during the week of student orientation
5	Find and cultivate a sponsor	Schedule meetings with MBRU academic leadership (**Dean/Associate Deans/Departmental Chairs**) to inform them about the benefits of PMR and the resources required.	6-weeks prior to commencement of Phase I
6	Prepare your target audience	⇒ Organize workshops in collaboration with the PMR team to inform stakeholders about “how” to disseminate PMR. ⇒ Circulate nano-lectures on PMR to stakeholders over WhatsApp.	1 week prior to the commencement of Phase I during the week of student orientation
7	Create the cultural fit	Create linkage between approaches to augment resilience and PMR to inform stakeholders “why” there is a necessity to create a culture of augment resilience in medical students and how PMR can address this objective.	4-weeks prior to commencement of Phase I
8	Develop and choose a leader team	Create an informal “Leader Team” consisting of stakeholders who enthusiastic about implementing PMR in the MBBS curriculum, such that they can guide and encourage the stakeholders to implement PMR.	1–5 weeks into the semester following the commencement of Phase I
9	Create small wins for motivation	Identify the stakeholders who successfully disseminated PMR and request them to present their experiences in this effort to the MBRU academic leadership and other concerned stakeholders.	4–5 weeks into the semester following the commencement of Phase I
10	Constantly and strategically communicate the change	During the whole transformation process: ⇒ Create a “Learning community” such that stakeholders can learn from each other about the benefits of PMR and the need to augment resilience in medical education. ⇒ Try to address hurdles that are faced by stakeholders in their endeavor, by communicating the change process to Sponsors	1–5 weeks into the semester following the commencement of Phase I
11	Measure progress of the change effort	⇒ Evaluate the attitude of stakeholders toward PMR following the transformation initiative using ADKAR (Awareness, Desire, Knowledge, Ability, Reinforcement) framework. ⇒ Assess the performance of the students in Phase – I to identify if PMR was beneficial to cultivate and augment resilience and reduce stress. ⇒ Conduct student feedback to assess the perception of students ⇒ toward PMR	10–12 weeks into the semester prior to conclusion of the first semester of Phase I
12	Integrate lessons learned	Using a reflective-framework conduct an After Action Review to: ⇒ Map the transformation process ⇒ Identify hurdles that further required to be tackled such that PMR can be successfully integrated the following semester.	14 week into the semester prior to conclusion of the first semester of Phase I
	Preparatory time for implementing the transformation		4-weeks
	Time required for implementing/assessing the transformation		5-weeks
	Total study duration (preparation + implementation + assessment)		14-weeks

### 4.8 Practical implementation of PMR in varied medical educational contexts

Building on our successful integration of PMR into CBMC using Mento's Change Management Model, we propose strategies for adapting this approach to various medical educational settings and its implications for curriculum development:

#### 4.8.1 Adaptation in different educational environments

Tailoring the PMR program to different institutional cultures and student demographics is crucial. We suggest using Mento's model as a flexible framework, allowing for contextual adjustments while maintaining the core components of PMR training. Collaboration with interdisciplinary teams can facilitate the customization of content, ensuring relevance and effectiveness across diverse medical education landscapes.

#### 4.8.2 Curriculum development across medical schools

Our findings can inform broader curriculum reforms, advocating for the inclusion of stress management and resilience training. We recommend embedding PMR within existing wellness and professional development modules, leveraging Mento's model to guide the integration process. This approach promotes a holistic educational experience, preparing students to handle the psychological demands of the medical profession.

#### 4.8.3 Faculty development and training

Key to the successful implementation of PMR in different settings is the training of faculty. Development programs based on Mento's model can equip educators with the necessary skills and knowledge to effectively teach PMR techniques, ensuring consistent delivery across various institutions.

#### 4.8.4 Evaluation and iterative improvement

Continuous assessment of the PMR program's impact, guided by change management principles, will enable ongoing refinement. This process should include feedback from students and faculty, allowing for iterative improvements tailored to each unique educational setting.

By extending the application of Mento's Change Management Model and our study's findings, medical educational institutions can effectively integrate PMR into their curricula, fostering a supportive learning environment that prioritizes mental health and resilience.

### 4.9 Strengths of the study

I. *Theoretical Grounding*—Our research employs a theory-driven approach to developing a student wellbeing program, specifically utilizing Gagne's nine events of instruction. This innovative method unveils a theoretical framework for effective stress management within medical education.
II. *Methodological Rigor*: Our research methodology is designed and executed, combining focus groups for instant feedback with Braun and Clarke's thematic analysis for an in-depth examination of qualitative data. The strict compliance with O'Brien's SRQR enhances the reliability and validity of our research outcomes.
III. *Effective PMR Implementation*: In response to the difficulties posed by the COVID-19 pandemic, our study introduces a practical solution using PMR. The feasibility of the program is highlighted by its time-efficient nature, which is especially valuable in the demanding and high-stress context of medical education.
IV. *Reenvisioning Stress Management in Medical Education Amidst COVID-19*: The unique and challenging circumstances of the COVID-19 pandemic have served as an impetus for reimagining our approach to stress management in medical education. Our study's PMR program not only provides an immediate solution but also fosters skills and competencies that will be of continued value in the post-COVID era. As students learn to effectively manage their stress through techniques like PMR, they cultivate resilience and adaptability—traits that will be crucial in navigating the evolving landscape of medical practice. Furthermore, the utility of our study extends its potential benefits to future pandemics or similar exigencies. The ability of the PMR program to equip medical students with coping mechanisms in the face of uncertainty contributes to their mental preparedness, thereby establishing a robust foundation for improved response to any forthcoming health emergencies.
V. *Interdisciplinary Engagement and Long-Term Impact in Medical Education*: Our research bridges various competency frameworks and Bourdieu's Theory of Practice, crafting a multifaceted approach to student wellbeing within the challenges of CBMC. This interdisciplinary framework enriches our study, positioning it at the nexus of medical education, psychology, and instructional theories. It identifies and elaborates on five interconnected themes—Self-control, Self-realization, Liberation, Awareness, and Interpersonal Relationships—providing a comprehensive view of how PMR influences both the personal and professional lives of medical students. Moreover, the study's impact transcends immediate stress relief, cultivating long-term resilience and mental preparedness. In today's world, increasingly faced with the likelihood of future pandemics or similar crises, this aspect of the study is especially crucial, laying the foundation for a more adaptable and psychologically resilient healthcare workforce.
VI. *Global Applicability, Societal Impact, and Practical Efficiency of Our Stress Management Program*: Our study introduces a stress management model that is notable for its effectiveness, cost-efficiency, and ease of implementation, positioning it as an ideal solution across a myriad of settings. Its design facilitates rapid integration into various structures, requiring minimal resources, which underscores its global applicability and adaptability to different cultures and environments. Crucially, the program's time-efficient nature is a significant asset. It is designed for quick learning and practice, making it particularly suitable for high-pressure environments like medical schools and hospitals. This feature is invaluable in situations where time is limited, ensuring stress management can be seamlessly incorporated into busy schedules. In the context of climate change and environmental stressors, such as the intense heatwaves in Europe and severe air pollution in cities like New Delhi, the program proves its worth by offering effective strategies for managing stress and anxiety. These tools are vital for individuals in regions undergoing rapid environmental changes, helping them cope with the psychological impacts of living under extreme conditions. Our study also extends its benefits to specialized sectors, such as healthcare, where it can enhance the resilience and emotional intelligence of professionals in high-stress fields like oncology. This improvement in stress management skills is essential for creating a supportive and empathetic care environment, crucial for patient recovery and wellbeing. The program is equally beneficial in unique and confined work settings like submarines or space stations, providing vital stress management skills crucial for mission success and crew safety.

Overall, the combination of cost-effectiveness, ease of implementation, and time efficiency makes our program a versatile and practical solution. Its adaptability ensures relevance in a broad spectrum of scenarios, from medical education pressures to the societal impacts of environmental stressors and the demands of specialized work environments.

## 5 Limitations and directions for future research

Our study presents pivotal insights into the effectiveness of PMR as a stress management technique within competency-based medical curricula; however, some limitations invite further exploration:

I. *Limited diversity in sample*: the study was conducted exclusively with female students, and personal factors like dietary habits, socio-economic status, and personal health were not assessed. Future studies could aim to include a more diverse sample and evaluate these additional factors to provide a more comprehensive view of the efficacy of PMR across varied demographic and lifestyle profiles.
II. *Genetic and epigenetic factors*: our study did not consider the potential impact of genetic variations, such as those in the CRHR1 (Mahon et al., 2013; Weeger et al., 2020; Zalachoras et al., 2022) and 5-HTTLPR genes (Zhang et al., 2009; Albert et al., 2019), which have been linked to differential stress responses. Similarly, epigenetic modifications like DNA methylation of the NR3C1 gene (Bakusic et al., 2017), could influence stress-resilience and the effectiveness of PMR. Future research should explore these genetic and epigenetic factors, potentially enabling a more personalized approach to stress management in medical education.
III. *Single institution study*: future research should validate our findings across multiple institutions, geographical regions, and diverse socioeconomic contexts to enhance external validity and generalizability.
IV. *Students from different curriculum phases*: participants in our study spanned various stages of their medical education, presenting varied stress management needs. Further research could examine the effectiveness of PMR programs at different stages of medical education, tailoring the intervention according to the specific needs at each stage.
V. *Long-term efficacy and academic performance*: While our study observed positive initial responses to the PMR program, its enduring efficacy remains to be investigated. Longitudinal studies should assess the long-term benefits of PMR, including its potential impact on academic performance, and how it contributes to stress management in the evolving post-COVID era.
VI. *Small sample size*: one key limitation of our study is the relatively small sample size, which may limit the generalizability of our findings. Future studies with larger cohorts are necessary to validate our results and provide more robust evidence on the effectiveness of PMR in medical education.
VII. *Specific context of a single medical school*: the study's context, limited to a single medical school, might have influenced the results due to unique institutional cultures or practices. Future research should aim to replicate the study in various medical schools with different educational environments to confirm the applicability of our findings across diverse educational settings.

Addressing these limitations in future research will augment our understanding of the role and benefits of PMR programs within competency-based medical curricula, thereby offering more nuanced guidance for medical educators and stakeholders.

## 6 Conclusion

Our investigation demonstrates the substantial benefits of integrating a PMR program within CBMC. This theory-guided approach, employing Gagne's nine events of instruction and Bourdieu's Theory of Practice, has yielded positive outcomes across thematic areas of *Self-control, Self-realization, Liberation, Awareness, and Interpersonal relationships*. Furthermore, the study has shown its potential to address the amplified stress medical students face during the COVID-19 pandemic and the unanticipated shift to distance learning.

As the first of its kind, this PMR program, while cost-effective and time-efficient, aligns with key domains of several global learning outcomes frameworks, confirming its applicability across diverse medical education contexts. Our findings underscore the role of PMR in fostering resilience, enhancing mental wellbeing, and equipping medical students with effective coping strategies. This proves particularly crucial considering the post-COVID era, wherein adaptable stress management strategies become indispensable in preparing a resilient healthcare workforce.

Nevertheless, our study also highlights areas of exploration for future research, such as evaluating genetic and epigenetic factors, examining the longitudinal benefits of PMR on academic performance, and adapting PMR interventions to varying student demographics and curriculum phases. Addressing these aspects would provide a more personalized, effective approach to stress management in medical education.

In summary, this research sets a meaningful precedent for the design and implementation of similar wellness initiatives within medical education, advocating for a shift toward a more comprehensive, student-centered approach that considers psychological health as a fundamental component of medical training. As we navigate the evolving challenges of medical education, such an approach is instrumental in shaping a resilient, adaptable, and mentally prepared healthcare workforce, aptly equipped to handle future crises.

## Data availability statement

The original contributions presented in the study are included in the article/Supplementary material, further inquiries can be directed to the corresponding author.

## Ethics statement

Consent was obtained from all individual respondents included in this study. This study was formally determined to be quality improvement, not human subjects research and was therefore not overseen by the IRB. There is no committee reference number to be given.

## Author contributions

In an expert collaboration, YB conceptualized the strategic approach for the dissemination of Progressive Muscle Relaxation (PMR), underpinned by the guiding theory of Gagne's model of instructional design and conceptuliased the study as well as its overarching aspects, from selecting the frameworks to utilize, procuring the requisite funding, to overseeing the final composition of the manuscript. BN took the lead in gathering data, executing qualitative analyses, and drafting the first draft of the manuscript. NN offered an instrumental hand in data analytics, authoring related manuscript sections, and preparation of illustrative figures. The literature review was pursued by YB, NN, and BN and updated by BS. The final version of the table corresponding to literature review was prepared by SJ. The Table in Appendix 1 was initially prepared by YB and NN, updated by BS. The initial bibliography was prepared by YB and was updated and revised by SK. The revised draft of the manuscript was prepared by YB and carefully proof-read by SK. SK also provided essential inputs to YB on improving the organization of the manuscript. This final revised version of the manuscript and the authorship has been endorsed by all authors. All authors contributed to the article and approved the submitted version.
