# Supplementary material for: Progressive muscle relaxation in pandemic times: bolstering medical student resilience through IPRMP and Gagne's model
Source: Front Psychol. 2024 Mar 13;15:1240791. doi: 10.3389/fpsyg.2024.1240791 (PMC10966386; doi:10.3389/fpsyg.2024.1240791)
Supplement: Supplementary file 1 [file Table_1.docx]

**Supplementary Table – 1**

| Year | Country of study | | Population Characteristics | Age Characteristics | Gender | Ethnicity | Mindfulness Practices | Outcome | Reference | |
| --- | --- | --- | --- | --- | --- | --- | --- | --- | --- | --- |
| 2022 | China | Nursing students (n=60) | | Mean age of 19.37 years. | 41 females were included in the study | Not reported | 8-week mindfulness training; 2-hour daily lectures. Administered SCS, EIS, and CAI at T1 (pre-training), T2 (post-training), and T3 (3-month follow-up) | The mindfulness group showed higher scores on SCS, EIS, and CAI than the control group at both T2 and T3, and improvements in emotional intelligence partially mediated the training effects on supportive communication | | *Perspect Psychiatr Care*, *58*(4), 2552-2561. |
| 2021 | Germany | Medical students in their preclinical years (n=143) | | Mean age of 20.4 years | 48.8% female and 51.2% male | Not reported | 6 two-hour MBSR courses using a peer-assisted-learning approach. Initial session: introduction to mindfulness and mindful breathing.  Subsequent sessions: group presentations on mindfulness publications. Post-session 1 feedback requested.  Homework: daily mindfulness practice and mindful activities. Course content: meditation, body-scan, mindful eating, gentle yoga. | Helped students preserve mindfulness and to contain the increase in stress over a period of at least 6 months. It had also helped in scholarly success but it was transient. | | *BMC Med Educ*, *21*(1), 145. |
| 2021 | Spain | Students in psychobiology course (n=143) | | Mean age of 20.28 years | 73.4% female | Not reported | 8 sessions of 2 hours each spread over 16 weeks. Supplementary mindfulness practice recordings provided. MBSR incorporated elements from Segal, Williams, and Teasdale's mindfulness-based cognitive therapy and its adaptation for non-clinical populations by Williams and Penman. | Significant reductions in stress and psy-chopathological symptomatology. However, no clear changes in the burnout scores were observed. | | *Mindfulness (N Y)*, *12*(5), 1138-1147. |
| 2021 | Chile | 4^th^ year medical students (n=123) | | Not reported | 48.8% female and 51.2% male | Not reported | Designed using Kern’s 6-step model. Included 3 live Zoom sessions and 5 self-paced online modules, led by trained tutors. Post-modules, students maintained a 4 two-week journal on emotions, ruminations, relationships, and habits, concluding with a written reflection. | Burn-out in students had decreased from 48% at baseline to 24% after the mindfulness program. Student’s feedback included lower levels of stress and self-blaming along with less traumatic stress reactions together. High resilience and increased use of active coping strategies were the main outcomes. | | *Rev Med Chil*, *149*(6), 846-855. |
| 2020 | Australia | Penultimate year students from clinical school (n=47) | | Mean age is 26.7 years | Male=9 and female=38 | Asian, Caucasian, Latin American and others | 8-week online program measuring meditation frequency/duration. Included mini lectures on compassion, communication, distraction reduction, effective task switching, emotion regulation, and guided mindfulness meditation. | Increased compassion and reduced stress were the main outcomes of the program. Increased self-awareness and emotional regulation were among the other results over a 4 month follow-up period. | | *BMC Med Educ*, *20*(1), 104. |
| 2020 | Brazil | First year medical students (n=141) | | Mean age is 18 years | Almost equal ratio of both genders | White and non-whites | 6 week mindfulness program inspired by Kabat-Zinn that involved conversations about mindfulness, practice of nonjudgmental listening awareness, mindful breathing and breathing exercises, handling thoughts and emotions, handling challenging situations, sitting meditation and awareness. Homework included audio recording for 10 min along with diary writing. | This study showed no significant differences between the control group and students who participated in the mindfulness program in terms of life, anxiety, stress and depression. | | *J Gen Intern Med*, *35*(3), 672-678. |
| 2019 | Norway | 1^st^ year medical (176)and psychology (112) (n = 288) | | Mean age of  24 years. | 219 females | Norwegian | Modified MBSR (7 weeks)  No elements of MBSR were removed, however time spent on exercises, didactic teaching and group reflection were shortened in response to feedback the investigators received from students during focus groups. The required home practice was also shortened to 30 minutes and the day-long retreat was kept as Session 7.  Booster session: During the 4-year period, students in the intervention group were invited to participate in an optional 1.5 hours booster session bi-annually, consisting of mindfulness practices (i.e. body scan, sitting or walking meditation, yoga, group dialogue session). | Intervention group showed significantly better scores on mental distress, mindfulness, avoidance coping and problem-focused coping | | *Mindfulness*, *10*, 1661-1672. |
| 2019 | UK | 5^th^ year medical students (n = 4); no control was used in this study, although the intervention group acted as the control as resilience and outcome measures were completed at baseline and on completion of the mindfulness intervention. | | Not reported | Not reported | Not reported | Mindfulness-based workshop: included overview, introduction to mindfulness practices, related evidence and potential impact of these practices. In the workshop, a mindfulness-based meditation was also carried out and the participants were taught the principles and practices of mindfulness.  Post-workshop intervention: 5 weekly 30-minute sessions of mindfulness training, which included a 15-minute mindfulness exercise. Following sessions included a follow-up meditation practice and a reflection on how mindfulness had been useful. | Resilience was completed using the Brief Resilience Scale (BRS) of Smith. Mindfulness was assessed using the Mindfulness attention awareness scale (MAAS). BRS scores increased from 18.5 to 22 (p = 0.02); MASS scores from a median of 2.5 to 3.73 (p=0.04). | | *Br J Nurs*, *28*(12), 798-802. |
| 2019 | USA | Preclinical, 1^st^ and 2^nd^ year medical students (n = 41; represents 11.4% of the target population).  Intervention and control groups consisted of 20 and 21 individuals, respectively. | | 20.4 years | 32 Females and 9 Males | Not reported | Intervention group underwent an 8-week mindfulness meditation course adapted from MBSR. The program consisted of 7 weekly 1.5-hour sessions, in addition to the introductory session. Each session included guided sitting and walking meditation. Students were also asked to practice mindfulness daily at home.  Both the intervention and control groups underwent an introductory session, consisting of a short didactic component including information on mindfulness in medicine coupled with guided sitting meditation, walking meditation, and mindful yoga. | The Five Facet Mindfulness Questionnaire (FFMQ), the Perceived Stress Scale (PSS), the Personal Health Questionnaire (PHQ-8) for depression, the Jefferson Scale of Empathy (JSE), and a Likert-type educational outcomes survey were used for evaluating the outcome. Mindfulness and awareness were negatively correlated with stress and depression. Students who took the full mindfulness course emerged with a greater familiarity with and willingness to utilize mindfulness modalities. There was no significant difference on wellness outcomes between the introductory-course-only group and the full-mindfulness-course group. | | *Med Sci Educ*, *29*(2), 439-451. |
| 2019 | USA | Preclinical 2^nd^ and 4^th^ year students (n = 45) | | Not reported | not reported | Not reported | Compassion Cultivation Training (CCT) program modelled after Stanford Center for Compassion and Altruism Research and Education’s (CCARE). The CCT program consisted of weekly, 2-hour sessions included pedagogical instruction, guided group meditation, mindfulness training, group discussion, listening and communication exercises, practical exercises related to weekly compassion themes, and 15–30 minutes of daily home meditation. | All students completed a pre/post-test of the Kentucky Inventory of Mindfulness Skills (KIMS), which is a validated, 39-item self-assessment instrument with a Likert-type scale ranging from one (never or very rarely true) to five (almost always or always true). This inventory assessed four different mindfulness facets, including: observing, describing, acting with awareness, and accepting without judgment. | | *BMC Med Educ*, *19*(1), 139. |
| 2019 | USA | Surgical residents (intervention group) and medical residents (control group) (n = 21) | | Not reported | 8 Females and 13 Males | Not reported | Weekly 2-hour modified MBSR classes and 20 minutes of daily home practice during an 8-week period. | The outcomes assessment tools included: Block Ego-Resilience scale, Cognitive and Affective Mindfulness Scale, Short Grit Scale, Perceived Stress Scale, Abbreviated Maslach Burnout Inventory, and 9-item Patient Health Questionnaire (PHQ-9). Mindfulness practices negatively correlated to burnout and positively with working memory, cognitive control and circle-cutting time. Blood oxygen level–dependent functional neuroimaging throughout an emotional regulation undertaking exhibited unique post-intervention activity in the intervention arm, in brain-areas associated with executive function control (dorsolateral prefrontal cortex) and self-awareness (precuneus). | | *JAMA Netw Open*, *2*(5), e194108. |
| 2019 | Australia | 2^nd^ medical students (intervention group, n = 45; control group, n = 45) (n = 90)  Age: Ethnicity: | | 20.19 | Female (67%) | Caucasian (74%) | Intervention group received a mindfulness app (My Student Mindfulness App). The app educates, reminds, prompts, and motivates students to practice mindful eating and stress reduction techniques designed along John Kabatt Zinn’s MBSR program.  Control group received an electronic self-monitoring diary for diet and exercise. The diary also had self-reflection space to think about key barriers one experiences. | As the aim of this study was to test the effectiveness, acceptability, and feasibility of a student-tailored mindfulness app for weight, weight-related behaviors, and stress, outcomes were measured using Perceived Stress Scale; Cognitive and Affective Mindfulness Questionnaire; Mindful Eating Questionnaire; Three Factor Eating Behavior Questionnaire and International Physical Activity Questionnaire. The intervention group had significantly lower stress levels (P=.02), lower emotional eating (P=.02), and uncontrolled eating (P=.02) as well as higher mindfulness (P≤.001) and mindful eating levels overall (P≤.001). | | *JMIR Mhealth Uhealth*, *7*(4), e12210. |
| 2018 | USA | 4 internal medicine physician teams consisting of 1 attending, 1 resident, 2-3 interns, and up to 4 medical students.  Due to the pilot nature of this study, demographic data for the teams (e.g., gender, age) was not collected. | | Not reported | Not reported | Not reported | A facilitated, group-based educational discussion on how mindfulness, as practiced through mindful hand hygiene, may improve clinical care and practices in the hospital setting. | The primary outcome was hand hygiene adherence (percentage) for each patient encounter. Other outcomes were observable mindful moments and mindful attention, measured using the Mindfulness Attention Awareness Scale, from baseline to post-intervention, and qualitative evaluation of the intervention. | | *BMJ Qual Saf*, *27*(10), 799-806. |
| 2018 | Netherlands | 11 Medical students, 15 Nursing students (n = 26) | | 19 – 45 years | Female (19), Male (7) | Dutch and Swedish | 1. Opening ritual: Gathering and focusing by lighting a candle and a short opening meditation  2. Check in: Sharing experiences/homework/feedback  3. Weekly topics (1 or 2 per session), covering: Breathing (various) Meditation (mindfulness/awareness, loving-kindness, walking, eating, concentrative)  Guided imagery (several types)  Biofeedback (autogenic training) Art (emphasis on noncognitive approaches) Yoga and tai chi Music (used in meditation and imagery sessions) Movement (shaking, dancing, exercise) Writing (reflective writing, journals, dialogues)  4. Closing ritual: Reflection on experiences during the session and a short closing meditation | The MB skills course was perceived as a remover of barriers, leading to new personal insights. Participation in the MB skills course made the participants gradually more aware of their own being, their feelings and emotions, and of the importance of taking care for themselves physically and mentally. The experiential sessions created more insight into the body–mind connection. This made the participants aware of the usefulness of incorporating MB practices in their own daily life to cope with stress and the corresponding benefits for their patients. | | *Global Qualitative Nursing Research*, *5*, 1-13. |
| 2018 | USA | 7 first-year psychiatry residents | | Not reported | Female (2), Male (5) | Not reported | The authors developed a course for all seven first-year psychiatry residents at their institution consisting of 1.5-h sessions for eight consecutive weeks. The first four sessions focused on mindfulness, and the last four sessions focused on empathy. | Based on qualitative course feedback, the PGY-1s found the material useful both in their personal lives and in their clinical work. They found the concepts provided a framework for recognizing their own habits and biases. | | *Acad Psychiatry*, *42*(5), 668-673. |
| 2018 | USA | 21 surgical interns in a residency training program at a tertiary academic medical center (intervention group, n = 11; control group, n =10) | | 24 – 31 years old | Female (8), Male (13) | Not reported | MBSR | This was a feasibility study, which shoed that Formal mindfulness-based stress-resilience training is feasible among academic surgery interns who find it to be acceptable and meaningful to their training experience. | | *JAMA Surg*, *153*(10), e182734. |
| 2018 | USA | 30 students during three consecutive Emergency Medicine clerkship rotations at a single, urban, academic institution (n = 30) | | Not reported | Not reported | Not reported | The curriculum included these components: (1) four, once-weekly, 60-minute classroom sessions; (2) prerequisite reading assignments; (3) individual daily meditation practice and journaling; and (4) the development of a personalized wellness plan with the help of a mentor. | Students believed more strongly in the importance of wellness for students and residents (p=0.01). They felt more confident that they could explain to another person how to meditate (p=0.0001) and be mindful (p=0.0001); more confident in their own ability to meditate (p=0.0001) and be mindful (p=0.0001); reported meditating more often (p=0.0001) and practicing mindfulness more often (p=0.0001); and were more likely to recommend meditation (p=0.0001) and mindfulness (p=0.0001) to another person. More importantly however, many of these changes remained sustained at six months later. | | *West J Emerg Med*, *19*(4), 762-766. |
| 2018 | USA | A total of 88 medical students (n = 88)  participants were categorised into intervention and control groups, no information regarding the number in each group was reported. | | 21 – 47 years old | Female (56), Male (32) | Asian/Pacific Islander (22); Black (6); Caucasian (41); Latino (5); Mixed (9); Other (5) | The intervention group was asked to download the smartphone application Headspace, an audio-guided mind- fulness meditation program. Headspace is a monthly digital subscription service that their participants accessed for free using 3-month subscription codes provided by the company for research purposes upon request. Its founder, Andy Puddicombe, developed the program by drawing from his personal experiences with meditation, as well as clinical and translational research that has found mindfulness to aid in stress reduction, improving life satisfaction, increasing focus and modifying self control. The mindfulness training program is structured such that each session lasts 10 min for the first 10 days, 15 min for the next 15 days, and 20 min for all subsequent sessions | Both groups completed baseline questionnaires on Qualtrics, an on- line survey system, including demographics, Perceived Stress Scale (PSS), Five-Facet Mindfulness Questionnaire (FFMQ), General Well-Being Schedule (GWBS). Perceived stress significantly decreased for the intervention group (p < 0.05). General well-being significantly increased for the intervention group compared to the control group from , and the increase was sustained p < 0.05. | | *J Altern Complement Med*, *24*(5), 505-513. |
| 2018 | Norway | 288 Norwegian medical (n = 176) and psychology (n = 112) students (intervention group, n = 144; control group, n =144) (n = 288) | | 24 years | Female (219), Male (69) | Norwegian | Students in the intervention group participated in a 7-week abridged Mindfulness-Based Stress Programme (MBSR). This was similar to that developed by Kabat-Zinn [42], but shorter in duration (reduced from 8 to 7 weeks), and less intense (reduced from 2.5-hr sessions to 1.5-hr sessions and from 45 to 20–30 min of recommended home-based mindfulness practice), with a full day of mindfulness practice retained at week seven. | Outcomes were subjective well-being, and dispositional mindfulness and coping assessed using the Five Facet Mindfulness Questionnaire and the Ways of Coping Checklist. At six-year follow-up, students receiving mindfulness training reported increased well-being. Furthermore, they reported greater increases in the trajectory of dis- positional mindfulness and problem-focused coping along with greater decreases in the trajectory of avoidance-focused coping. Increases in problem-focused coping predicted increases in well-being. These effects were found despite relatively low levels of adherence to formal mindfulness practice. | | *PLoS One*, *13*(4), e0196053. |
| 2017 | Netherlands | 148 medical residents across all discipline (intervention group, n = 80; control group, n =68) (n = 148) | | 31.2 years | Female (130), Male (18) | Dutch | MBSR consisted of eight weekly 2.5-h sessions and one 6-h silent day. | The primary outcome was the emotional exhaustion subscale of the Dutch version of the Maslach Burnout Inventory–Human Service Survey. Secondary outcomes included the depersonalization and reduced personal accomplishment subscales of burnout, worry, work–home interference, mindfulness skills, self- compassion, positive mental health, empathy and medical errors. Assessment took place at baseline and post- intervention approximately 3 months later. No significant difference in emotional exhaustion was found between the two groups. However, the MBSR group reported significantly greater improvements than the control group in personal accomplishment (p = 0.028, d = 0.24), worry (p = 0.036, d = 0.23), mindfulness skills (p = 0.010, d = 0.33), self-compassion (p = 0.010, d = 0.35) and perspective-taking (empathy) (p = 0.025, d = 0.33). | | *J Gen Intern Med*, *33*(4), 429-436. |
| 2017 | UK | 29 undergraduate medical students (n = 29) | | 24.4 years | Female (60.1%), Male (39.9%) | Not reported | The WMS wellbeing and personal development programme was . The programme comprises lectures, group work and self-directed material for further study. Group work occurs in stable groups with a trained facilitator. Each session (1-2 hours in length) includes both a brief guided mindfulness exercise and a group task focusing on personal and professional development topics. | This was a mixed methods study. In the context of an overall decline in wellbeing and limited engagement with mindfulness practice, increases in mindfulness were protective against this decline in all students (p<001). A small minority of students thought the course was a waste of time. Their attitudes influenced engagement by their peers. | | *Health Education*, *118*(4), 304-319. |
| 2017 | Netherlands | 19 residents from medical, surgical, and primary care disciplines (n = 19) | | 32 years | Female (14), Male (5) | Dutch | MBSR | Residents indicated that the MBSR training increased their awareness and self-reflection at work, and they were more accepting toward themselves and toward their limitations. Furthermore, they mentioned being more resilient and better at setting priorities and limits. They improved their self-care and work–life balance. | | *Acad Med*, *93*(9), 1335-1340. |
| *2017 | USA | 105 medical students (n = 105) | | Approximately 25 years | Female (54), Male (51) | Not reported | The intervention was based on the Stress Management and Resilience Training (SMART) program previously found to improve stress, anxiety, and overall QOL among volunteers (readers can refer to the reference for the specifics of the protocol).  Sood A, Prasad K, Schroeder D, Varkey P. Stress management and resilience training among department of medicine faculty: a pilot randomized clinical trial. J Gen Intern Med 2010;26:858-61. | Validated instruments were used to examine the effects on burnout, quality of life (QOL), stress, resilience, happiness, and empathy. Paired analysis was conducted to explore changes from baseline. Required longitudinal mindfulness- based stress management course tested did not lead to measurable improvements in medical student well-being or empathy. | | *J Gen Intern Med*, *32*(12), 1309-1314. |
| 2017 | USA | 188 medical residents from all specialties (n = 188) | | Not reported | Not reported | Not reported | Residents were asked to attend a 60-minute didactic featuring both the concepts and science behind well-being. Then they attended 15-minute booster sessions during protected didactic time each week for a 12-week curriculum. | No direct results were reported, however the authors reported that it is feasible to implement a low-cost, peer-led wellness curriculum during residency. | | *MedEdPORTAL*, *13*, 10651. |
| 2017 | Canada | 15 medical residents from all specialties (n = 15) | | 30.6 years | Female (14), Male (1) | Not reported | Arts-based visual literacy and mindfulness practice (Art of Seeing) – Refer to article for protocol. | Participants underwent assessments using three validated psychometric scales—Compassion Scale, Interpersonal Reactivity Index (IRI), and Mindfulness Scale—on the first and last days of the program. Fourteen participants engaged in semi-structured interviews exploring the perceived impacts of the program on their empathic clinical practice. In comparison to a control group, program participants showed improvement in the Mindfulness Scale domains related to self-confidence and communication. While most psychometric measures didn't indicate significant differences between groups during the program, qualitative analysis of interview data revealed positive impacts on participants' perceived empathy toward colleagues and patients, as well as on personal and professional well-being. | | *Med Humanit*, *43*(3), 192-198. |
| 2017 | Netherlands | 167 students (intervention group, n = 83; control group, n =84) (n = 167) | | Any individual younger than 18 years of age was not allowed to participate. | Not reported | Dutch | MBSR | Follow-up period was 20 months. MBSR appeared feasible and acceptable to medical clerkship students and resulted in a small to moderate improvement of mental health compared to the control group. No significant effect on physician empathy observed for the intervention group. | | *Acad Med*, *92*(7), 1012-1021. |
| 2017 | Australia | 44 Intern doctors completing an emergency department rotation in a major Australian hospital (intervention group, n = 23; control group, n =21) (n = 44) | | 22 – 48 years | Female (64%), Male (36%) | Not reported | The intervention was a mix mindfulness education and practice. Material was adapted from well-validated psycho- logical treatment programs (Mindfulness-Based Stress Reduction, Mindfulness-Based Cognitive Therapy, and Acceptance and Commitment Therapy). Adaptations to this material were necessary so that it was applicable for a non- clinical population. The 10 sessions focused on the following themes: (1) Introducing mindfulness, (2) Everyday awareness and automatic pilot, (3) Barriers to being mindful, (4) Mindfulness of breathing theory and activities, (5) Staying present at work and daily like, (6) Letting go of sensations and emotions, (7) The nature of thoughts, (8) Self- care, (9) Applying what has been taught, and (10) Review | Significant reduction in stress and burnout was observed for participants in the intervention group. | | *Med Teach*, *39*(4), 409-414. |
| 2017 | New Zealand | 83 medical students (number of students allocated to individual groups is not stated) (n = 83)  Participants were gender block-randomized to mindfulness or control groups. | | 18 – 37 years | Female (54.2%), Male (45.8%) | Not reported | The intervention group underwent a mindfulness induction process. The mindfulness induction consisted of information about mindfulness and experiential exercises designed to increase state mindfulness. The details of these exercised can be obtained from Erisman, S., & Roemer, L. (2010). A preliminary investigation of the effects of experimentally induced mindfulness on emotional responding to film clips. Emotion, 10(1), 72. | Mindfulness induction showed some promise in enhancing compassionate responses and behaviour among medical students. | | *Mindfulness*, *8*, 276-285. |
| *2016 | Germany | 182 medical and dental students (2 intervention groups: 1. MediMind (n=66), 2. Autogenic (n=73; control group, n = 43) | | 23.4 years | Female (84%), Male (16%) | German | MedMind and Autogenic study protocols are described in detail in the following article: Kuhlmann SM, Bürger A, Esser G, Hammerle F. A mindfulness-based stress prevention training for medical students (MediMind): study protocol for a randomized controlled trial. Trials. 2015;16(1):40. | Due to the high and selective dropout rates, the results cannot be generalized and further research is necessary. | | *BMC Med Educ*, *16*(1), 316. |
| 2016 | USA | 33 pediatric residents at the University of Chicago (n = 33) | | Not reported | Not reported | Not reported | Participants used a free smartphone application: Headspace to complete a 10-day program in mindfulness meditation and completed surveys at the end of the program. Each session was comprised of a 10-minute recording, which was a combination of educational material and a short-guided meditation. Some of the sessions were completed in a group setting as parts of scheduled resident education conferences, but residents completed most on their own. | Participants completed surveys before and after the study, covering demographic details, perceptions of mindfulness, and its application. Additionally, validated questionnaires—the abbreviated Maslach Burnout Inventory (aMBI) and the Mindful Attention Awareness Scale—were used. Post-intervention, a higher percentage of residents viewed mindfulness as a valuable patient intervention, and a statistically significant increase occurred in those intending to discuss mindfulness as a therapeutic option. Residents also reported a high sense of personal accomplishment. | | *Pediatr Ann*, *45*(10), e373-e376. |
| 2016 | USA | 26 medical students (n = 26) | | Not reported | Female (54%), Male (46%) | Not reported | Reflective practice involved participants setting learning goals at the start of the rotation. Daily, they wrote a reflective paragraph, analyzing performance, experiences, and outlining plans for applying acquired knowledge. Reflection covered diverse topics, including challenging cases, proficiency in new exam techniques, reactions to clinical situations, and decision-making for patients. Weekly, participants submitted reflections for written feedback from the rotation director. Feedback aimed to help students identify missed opportunities and guide them in developing specific, actionable plans based on their reflections. | A greater sense of mindfulness and focus on self-improvement were major themes that emerge from students’ descriptions of the role of daily reflections in their learning. | | *Perspect Med Educ*, *5*(5), 285-291. |
| 2016 | UK | 228 medical students (n = 228) | | 21-40 years | Female (125), Male (103) | Not reported | MBSR course but condensed it in terms of time (reduced from 8 weeks to 7 weeks; 1 to 2 h per week). | The MBSR course was associated with high levels of satisfaction and positive feedback amongst the participants. | | *BMC Med Educ*, *16*(1), 209. |
| 2016 | Norway | 144 medical students (n = 144) | | 25 years | Not reported | Norwegian | MBSR | All participants reported increased attention and awareness of psychological and bodily phenomena. The majority also reported a shift in their attitudes towards their experiences in terms of decreased reactivity, increased curiosity, affect tolerance, patience and self-acceptance, and improved relational qualities. The experience of mindfulness was mediated by subjective intention and the interpretation of mindfulness training. | | *Mindfulness (N Y)*, *7*, 838-850. |
| 2016 | New Zealand | 275 medical students (intervention group, n = 133; control group, n = 142) (n = 275) | | 25 years | Female (145), Male (130) | Not reported | Medical students, trained as peer leaders, delivered the intervention by providing support, teaching mindfulness meditation, and organizing weekly peer-led mindfulness sessions. They encouraged intervention participants to engage in individual mindfulness practice at home using a provided mindfulness CD. Regular email updates about the location of weekly sessions were sent. Participants were prompted to seek support from peer leaders after sessions or independently. Additionally, peer leaders arranged two social events—a bowling outing and a cupcake party—for intervention participants. | Primary measures included depression (PHQ-9) and anxiety (GAD-7) scores. Secondary measures were quality of life, resilience (15-item resilience scale), academic self-concept, and motivation to learn, assessed at baseline and 6 months. Improvements in mental health were seen in the intervention group, the difference between the intervention and nonintervention groups did not reach statistical significance. | | *Teach Learn Med*, *28*(3), 293-302. |
| 2016 | USA | 52 medical students (intervention group, n = 28; control group, n = 24) (n = 52) | | 23.5 years | Female (63%), Male (37%) | 65.4% Caucasian, 15.4% Asian, 7.7% biracial and multiracial, 5.8% as Black or African American, and 5.8% Other | The mind–body program was an 11-week, 2-hour skills training group that focused on introducing, practicing, and processing mind–body skills such as biofeedback, guided imagery, relaxation, several forms of meditation (e.g., mindfulness), breathing exercises, and autogenic training. | Students in the mind–body group showed a modest improvement in all distress tolerance subscales over time whereas the control group showed less consistent changes. Students in the mind–body group qualitatively reported an improved ability to tolerate affective distress. Overall, improvements in psychological symptoms were associated with improvements in distress tolerance in the mind–body group but not in the control group. | | *Teach Learn Med*, *28*(2), 219-228. |
| 2015 | Malaysia | 75 medical students (intervention group, n = 37; control group, n =38) (n = 75) | | 22.2 years | Female (84), Male (51) | 70 (52 %) were Chinese, 57 (42 %) Malays, and 8 (6 %) Indians | A brief (four-session, 2 h per week) group Mindfulness-based Cognitive Therapy (b-GMBCT/Mindful-Gym). | Participants were administered four self-report questionnaires twice: (a) 1 week prior to the commencement of the program, and (b) at the end of the program (1 week after the last session). The following outcome variables were measured pre-and post-intervention: mindfulness, perceived stress, and general psychological distress. Participants reported significant reductions in perceived stress, general psychological distress, as well as increase in mindfulness with medium effect sizes after attending the program. | | *Adv Health Sci Educ Theory Pract*, *20*(5), 1115-1134. |
| 2015 | USA | Participants (n = 44) included medical and physician-scientist (MD/PhD) students of which 36 (82%) completed the workshop | | Not reported | Female (29), Male (7) | Not reported | Mind–Body Medicine (MBM) workshop was adopted. The workshop was based on an 11-week course developed by the Center for Mind–Body Medicine in Washington, DC. The adapted workshop for medical students consisted of four weekly 1.5-hour small group sessions and home practice of mind–body skills in between group meetings. In addition, students were also asked to choose (and monitor) a weekly self-care goal. | MBM allowed students to deal with the stress and emotional challenges of medical school, and helped increase self-care behaviors, such as exercise, sleep, and engaging in social support. | | *Explore (NY)*, *11*(3), 186-192. |
| 2015 | Malaysia | 75 medical students (intervention group, n = 37; control group, n = 38) (n=75) | | 21 years | Female (57), Male (18) | Malay (40), Chinese (28), Indian (7) | A 5-week mindfulness-based stress management (MBSM) program which was adapted from and based on the principles of the 8-week MBSR and MBCT programs. | The intervention group exhibited significant reduction in perceived stress, mental distress; and increased levels of mindfulness and self-efficacy, compared to control group. | | *Adv Health Sci Educ Theory Pract*, *20*(5), 1115-1134. |
| 2015 | Norway | 288 medical students (intervention group, n = 144; control group, n = 144) (n=288) | | 21 years | Female (219), Male (69) | Norwegian | MBSR | The study examined how baseline personality factors (neuroticism, conscientiousness, and extroversion) and mindfulness at baseline influenced mental distress, study stress, and subjective well-being post-intervention. Higher neuroticism scores correlated with an amplified intervention effect on mental distress and subjective well-being. For those with higher conscientiousness scores, mindfulness training had an increased impact on reducing study stress. Overall, the training shielded students from the control group's rise in mental distress and study stress and decline in subjective well-being. | | *Mindfulness (N Y)*, *6*(2), 281-289. |
| 2015 | Malaysia | 139 medical students (intervention group, n = 82; control group, n =57) (n=139) | | 22.4 years | Female (61.2%), | Malay (48.5%) | A 5-week (MBSM) program which was adapted from and based on the principles of the 8-week MBSR and MBCT programs. | Intervention group reported significantly greater improvements in depressive symptoms, anxiety, general psychiatric symptoms, perceived stress, subjective happiness, and satisfaction with life compared to the control group. | | *International Journal of Cognitive Therapy*, *8*(4). |
