# Supplementary material for: Progressive muscle relaxation in pandemic times: bolstering medical student resilience through IPRMP and Gagne's model
Source: Front Psychol. 2024 Mar 13;15:1240791. doi: 10.3389/fpsyg.2024.1240791 (PMC10966386; doi:10.3389/fpsyg.2024.1240791)
Supplement: Supplementary file 2 [file Data_Sheet_1.pdf]

Supplementary Material

Enhancing Medical Student Resilience During Pandemics: Implementing Progressive Muscle Relaxation (PMR) through Gagne's Nine Events-Based Approach and the Introduction of the Integrative Psychological Resilience Model in Medical Practice (IPRMP) – A Pioneering Investigation

Bhavana Nair[1,$], PhD; **Nerissa Naidoo**[1,$], PhD; **Sara Khan**[1], BSc; **Balamohan Shivani**[1,£], BTech; **Shirin Jannati**[1], HSC; and **Yajnavalka Banerjee**[1,2,$,*], PhD, PGDME

[1]College of Medicine and Health Sciences, Mohammed Bin Rashid University of Medicine and Health Sciences (MBRU), Dubai, United Arab Emirates.

[2]Centre for Medical Education, University of Dundee, Dundee, United Kingdom.

[£]**Current address:** Lund University, Box 117, 221 00 Lund, Sweden.

[$]The authors would like to inform the journal that the authors agree **B. Nair**, **N. Naidoo**, and **Y. Banerjee** have all completed the intellectual and other work typical of the first author.

***Correspondence:**
Yajnavalka Banerjee,
Department of Basic Medical Sciences, MBRU,
Academic Medical Centre, Building 14,
Dubai Health Care City (DHCC),
Dubai 505055,
United Arab Emirates (AE).
Emails: yaj.banerjee@gmail.com ; YBanerjee@dundee.ac.uk;
Twitter: @YajB_PhDPGDME

1 Supplementary Data: Progressive Muscle Relaxation Script

Progressive muscle relaxation is an exercise that relaxes your mind and body by progressively tensing and relaxation muscle groups throughout your entire body. You will tense each muscle group vigorously, but without straining, and then release the tension and feel the muscle relax. You will tense each muscle for about 5 seconds. Throughout this exercise you may visualize the muscles tensing and a wave of relaxation flowing over them as you release that tension. It is important that you are aware of your breathing throughout the exercise.

Now let's begin. Make yourself absolutely comfortable - it is best if you do this in a sitting position with your feet firmly placed on the ground without your footwear. You may also lie down if you wish. Allow your attention to focus only on your body. If you begin to notice your mind wandering, try and bring it back to the muscle you are working on. You may want to close your eyes or if you choose to keep them open, just soften your gaze. You may keep hands on your lap with your palms

placed one on top of the other so that the positive energy that you generate during the session remains within your body.

Take a deep breath through your abdomen, hold for a few seconds, and exhale slowly. As you breathe, notice your stomach rising and your lungs filling with air. As you exhale, imagine the tension in your body being released and flowing out of your body. And again inhale..... and exhale. Feel your body already relaxing.

You will be asked in this session to tense certain muscle group but without straining, and then to release the tension and let your muscle relax. The intention is to help you become aware of the difference between tension and relaxation. If you have any pain or discomfort in any of the targeted muscle groups feel free to skip that step.

Take a deep breath through your abdomen, hold for a few seconds, and exhale slowly. Again, as you breathe notice your stomach rising and your lungs filling with air. As you exhale, imagine the tension in your body being released and flowing out of your body. And again inhale..... and exhale. Feel your body starting to relax.

Now let's begin the exercise.

Focus on your feet that are firmly placed on the ground if you are sitting. Slowly, curl your toes towards your body and hold the position to the count of 5. Feel the tension around your toes before you slowly release and bring them back to position.

(Pause for 10 seconds)

Next, tighten your thighs by pressing your knees together, as if you are holding an imaginary pillow between them. Hold for 5 seconds...and feel the tension around your thighs and hips before you slowly release the muscle and feel your thighs relaxing.

(Pause for 10 seconds)

Now tighten the muscles in your stomach by sucking it in. Hold for 5 seconds, and feel the tension around your stomach. Now slowly release.

(Pause for 10 seconds)

Tighten your chest by taking a deep breath in, hold for about 5 seconds, and exhale through your mouth, blowing out all the tension and become aware of the way your chest feels. It feels as though a weight has been taken off your chest.

(Pause for 10 seconds)

Now tense your back muscles by thrusting your chest forward, making your shoulder blades move towards each other. Feel the tension in your upper back and lower back to the count of 5. Now slowly release the tension from your back and try to relax your body as much as you can.

(Pause for 10 seconds)

Next, lift your shoulders up as if they could touch your ears to the count of 5. Feel the stress around your shoulders, neck and ears. Relax your shoulder muscles and feel the tension leave your shoulders and your neck.

(Pause for 10 seconds)

Now clench your fists tightly as if you were holding onto something very tightly. Feel the buildup of tension in your fist that radiates all the way up your arm to the count of 5. Slowly open your palm and feel the relaxation around your hand.

Take a deep breath in, now slowly exhale. You feel a sense of relaxation and calmness starting to build up in your entire body. Enjoy this feeling of peace and relaxation as your body relaxes.

(Pause for 10 seconds)

Now gently pull your head back as if you were to look at the ceiling to the count of 5. Feel the stretch at the back of your neck. Slowly bring your head back to position, feeling the tension melting away from the back of your neck.

(Pause for 10 seconds)

I want you to now smile as widely as you can, and feel the tension around your lips and cheeks. Hold the smile to the count of 5. Now slowly release, appreciating the softness returning to your face.

(Pause for 10 seconds)

Next, tense your eye muscles by squeezing your eyes tightly to the count of 5. Feel the tension around your eyes, your nose and your upper cheek. Slowly release the tension and feel the difference.

(Pause for 10 seconds)

Tighten the muscles in your forehead by raising your eyebrows as high as you can to the count of 5. Release your eyebrows and feel the difference. Since there is a lot of tension usually in and around the face, I want you to bring your eyebrows together as you would do if you were to frown and hold the position to the count of 5. Feel the tension in and around your forehead and your eyes, now slowly release the muscle and feel the tension leave the area.

Take a deep breath in and allow your whole body to relax and as you breathe out, releasing all the tension from your body and becoming aware of how relaxed your body feels in this moment.

Now imagine a wave of relaxation slowly spreading through your body beginning at your head and going all the way down to your feet. And as you do this moving any part of the body that still seems to be tense, releasing all the residual tension that may still be in your body.

I want you to stay with this feeling of relaxation for the next one minute, observing the natural breath that comes in and goes out of your nose. If any thought comes to your mind, allowing it to drift away like a cloud in the sky, without holding on to it.

Breathe in.. and breathe out..... Staying in the moment feeling relaxed, calm and at peace.

Whenever you are ready, rub your hands together to generate some heat and place it over your eyes and feel the energy flow down your body. Slowly open your eyes, and ground yourself by becoming aware of your surroundings.

(Adapted from The Anxiety & Phobia Workbook, by Edmund J.)
